# Chalcogen bonding-directed photoresponsive helix from azo-fused H-bonded arylamide foldamers

**DOI:** 10.1039/d5sc10108e

**Published:** 2026-05-05

**Authors:** Chuan-Zhi Liu, Chi Zhang, Hua-Shuo Zheng, Liu-Xin Luo, Shuang-Shuang Hou, Song-Hao Guo, Meng-Ge Zhang, Xin-Yao Peng, Liang Xu, Bin Zhai

**Affiliations:** a Engineering Research Centre for Optoelectronic Functional Materials of Henan Province, College of Chemistry and Chemical Engineering, Shangqiu Normal University Shangqiu Henan 476000 China liuchuanzhiuniv@163.com liuchuanzhi@sqnu.edu.cn zhaibin_1978@163.com; b School of Chemistry and Chemical Engineering, State Key Laboratory Incubation Base for Green Processing of Chemical Engineering, Shihezi University Shihezi Xinjiang 832003 China xuliang4423@shzu.edu.cn

## Abstract

In this study, a series of azo-fused H-bonded arylamide foldamers was synthesized. Molecules with extended backbones exhibited pronounced photoresponsive behavior in both dichloromethane and dimethyl sulfoxide. In low-polarity dichloromethane, intramolecular three-center hydrogen bonding remained stable, promoting the formation of well-defined S-shaped (*E*) and helical (*Z*) conformations. In contrast, disruption of the intramolecular three-center hydrogen bonding induces a structural transition to linear (*E*) and U-shaped (*Z*) configurations in highly polar dimethyl sulfoxide. With respect to the foldamer featuring benzoselenadiazole termini, the orthogonal interplay between intramolecular hydrogen bonding and intermolecular Se⋯N interactions synergistically drove the assembly of linear and helical secondary architectures, which was attributed to the inherent properties of the hydrogen-bonded arylamide foldamer combined with enhanced dual intermolecular Se⋯N chalcogen bonding. Furthermore, reversible structural interconversion was achieved through alternating irradiation at 365 nm and 600 nm. The conformational dynamics and structural transformations were rigorously characterized and computationally validated using nuclear magnetic resonance (NMR) and UV-vis spectroscopy, as well as density functional theory (DFT) calculations, and molecular dynamics (MD) simulations. This work presents an innovative supramolecular helical self-assembly directed by noncovalent interactions, wherein the resulting helices demonstrate distinctive photoresponsive properties.

## Abstract 2

The self-assembly of a photoresponsive supramolecular helix orthogonally guided by dual intermolecular Se⋯N chalcogen bonding and intramolecular hydrogen bonding.

## Graphical abstract

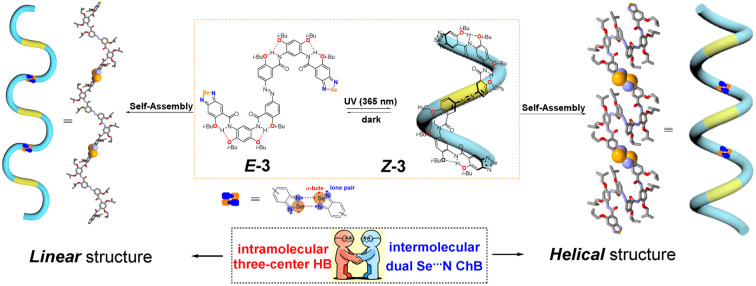

## Introduction

Significant efforts have been directed toward the development of diverse artificial secondary structures to elucidate the principles by which noncovalent interactions govern molecular conformational organization. These endeavors aim not only to construct novel ordered architectures for applications in molecular recognition and self-assembly but also to identify lead scaffolds with specific biological activities. Among these artificial systems, helices represent one of the most structurally rich, versatile, and functionally diverse classes of structures. To date, a wide variety of artificial helical architectures have been reported worldwide, ranging from polymer-based helices constructed *via* covalent linkages^1–3^ to supramolecular helices formed through noncovalent interactions such as solvophobic effects,^4^ donor–acceptor interactions,^5–13^ and metal-coordination bonds.^14–16^ Notably, hydrogen bonding (HB),^5–8^ halogen bonding (XB),^9,10^ and chalcogen bonding (ChB)^11–13^ have emerged as the most extensively studied driving forces for helical formation. Unlike dipole–dipole (or dipole–induced-dipole) interactions from HB, ChB represents a subclass of σ-hole interactions. Such interactions arise from attractive forces between an electrophilic chalcogen atom (S, Se, Te) and a Lewis base, typically an electron-rich atom or molecule. In contrast to XB, where halogen atoms feature a single σ-hole, divalent chalcogen atoms in particular possess two mutually perpendicular σ-holes, corresponding to the two lone pairs on the chalcogen center. This unique structural feature renders ChB significantly more flexible than XB.^17,18^ The overall interaction energy of ChB comprises several components: electrostatic interactions, n → σ* charge transfer, and dispersion forces—the latter being especially important for heavier chalcogens such as Se and Te due to their larger polarizabilities.^19–21^

Despite significant advances in supramolecular chemistry, the integration of stimuli-responsive functionalities into helical supramolecular architectures remains a formidable challenge, with limited success reported to date.^22–26^ The rational design of stimuli-responsive supramolecule helices demands simultaneous control over two intertwined parameters: (1) the kinetic accessibility of conformational switching pathways, and (2) the stability and reversibility of dynamic responses.^27,28^ However, most current approaches rely on weak noncovalent interactions to stabilize helices, which inevitably compromises their responsiveness to external triggers. Consequently, achieving reversible helical transitions without structural collapse remains a critical bottleneck in the field, as evidenced by the scarcity of robust examples in the literature. Therefore, constructing supramolecular helical structures with stimuli-responsive functionalities and investigating conformational transitions involving unwinding–rewinding dynamics during helical stimulation–response processes are critical for deciphering the dynamic encapsulation and release mechanisms of native peptide frameworks. Such investigations not only provide molecular-level insights into the structural plasticity governing biomolecular interactions but also advance our fundamental understanding of their mechanistic roles across diverse biological processes.^29^

Hydrogen-bonded foldamers constitute a class of artificially designed molecules that adopt well-defined conformations stabilized by intramolecular hydrogen bonding networks, typically constructed from aromatic amide^5–8,10,30–36^ or peptidomimetic backbones.^4,6,37–40^ Through systematic elongation of repeating units, their supramolecular architectures undergo a conformational evolution from crescent-shaped motifs to larger macrocyclic or helical assemblies, driven by cumulative π-stacking and HB interactions. Owing to their preorganized cavities and dynamic conformational adaptability,^5–8,31,35,36^ these systems exhibit remarkable utility in molecular/ionic recognition and transmembrane transport applications. Notably, Yuan and coworkers pioneered a series of photoresponsive hydrogen-bonded azo-macrocycles—a novel kind of covalent macrocycles derived from light-switchable foldamer precursors. By integrating azobenzene moieties into the hydrogen-bonded framework, these constructs demonstrate reversible guest encapsulation behavior under photochemical control, thereby opening new avenues for stimuli-responsive supramolecular systems in controlled release and related fields.^35,41,42^

**Scheme 1:**
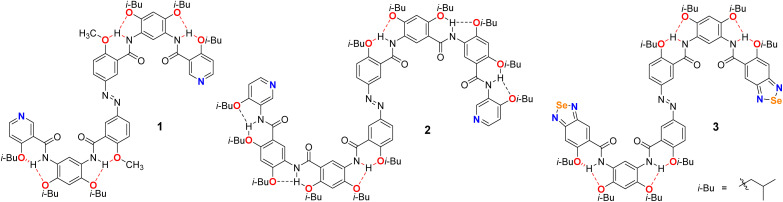
The structures of compounds 1–3.

Based on the exceptional spatiotemporal precision, noninvasive operational modality, highly selective stimulus responsiveness, and ecologically benign attributes inherent to light-responsive systems,^35,41–43^ the photoresponsive azobenzene moiety was covalently integrated into H-bonded arylamide foldamers. The design yielded a novel class of azo-fused H-bonded arylamide foldamers (H-bonded-Ph-N=N-Ph-H-bonded: 1, 2, and 3) (Scheme 1), enabling the construction of a photoresponsive supramolecular assembly system with precisely tunable, light-mediated dynamic behaviors. Compounds 1 and 2 were designed around azobenzene scaffolds, each functionalized with pyridine groups at both termini. The aromatic cores were connected through amide linkages to form well-defined backbones. Furthermore, the incorporation of isobutoxy groups on aromatic rings not only established a robust structural framework conducive to three-center hydrogen bonding but also significantly enhanced molecular crystallinity. Compound 1, featuring a relatively shorter backbone, was initially employed as a model system to investigate the fundamental structural features and photoresponsive behavior of this foldamer family. In comparison, the longer compound 2, characterized by a more pronounced spatial configuration, was utilized to further explore the solvent-dependent and photoresponsive characteristics inherent to this class of azo-fused H-bonded arylamide foldamers. Compound 3 shared a structural framework similar to that of compound 1 but incorporated a benzoselenadiazole moiety at the terminal position. Notably, benzoselenadiazole was strategically introduced as the core heterocyclic motif to mediate ChB interactions, functioning simultaneously as a dual-role electron donor and acceptor without the need for supplementary guest molecules. This intrinsic bifunctionality significantly enhanced the structural robustness and assembly efficiency of the resulting supramolecular architecture.^13,44,45^ Based on compound 3, ChB-driven helical supramolecular assembly was explored, and its photoresponsive properties were evaluated.

## Results and discussion

**Fig. 1:**
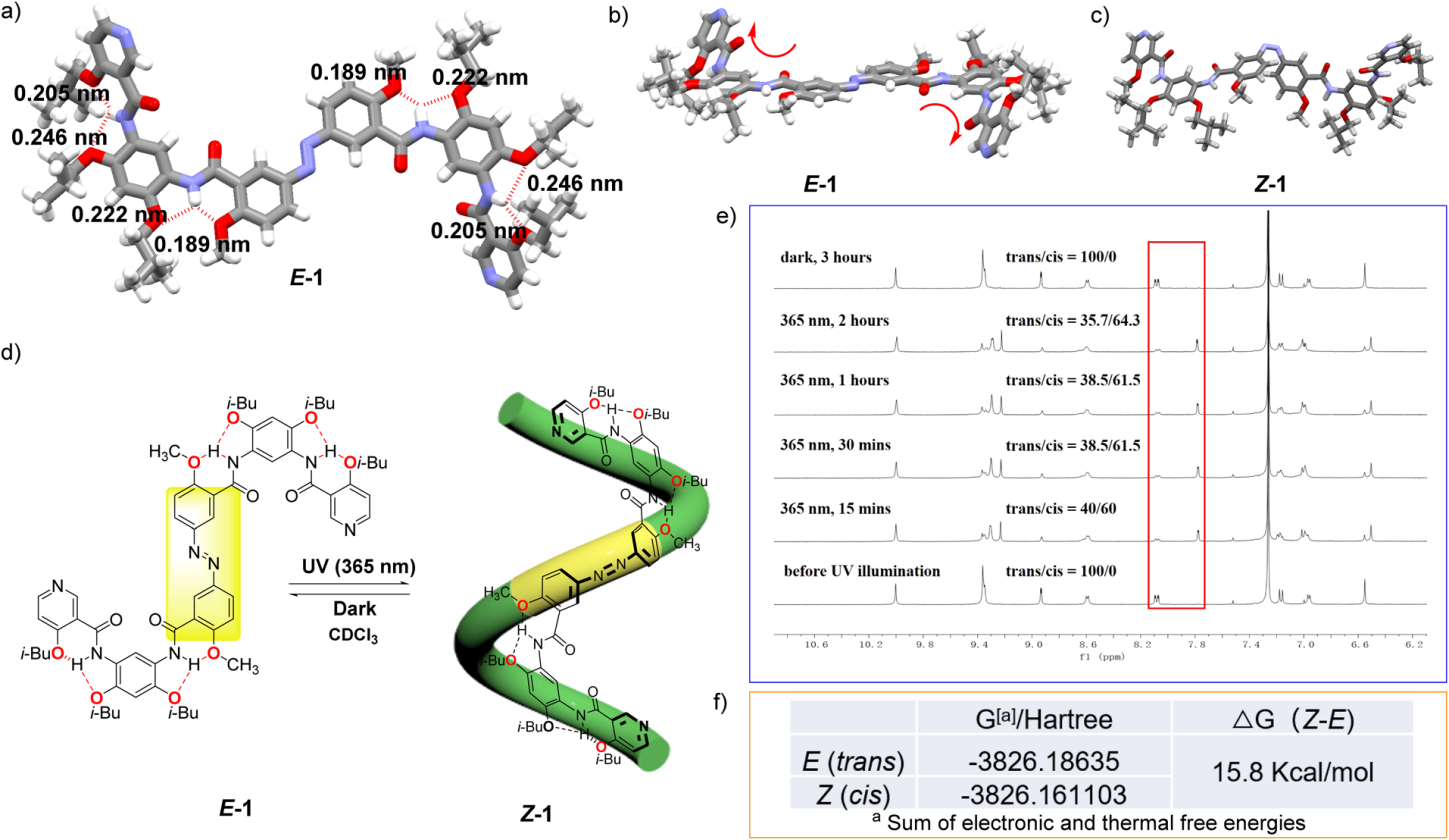
(a) Crystal structure of the *trans*-configuration of compound 1, with the hydrogen bond length data indicated. (b) Side view of the crystal structure of the *E* configuration of compound 1. (c) *Z* configuration of compound 1 simulated by a computer. (d) The photoresponsive interconversion between the *E* and *Z* configurations of compound 1 (irradiation with 365 nm UV light and subsequent dark storage), wherein the *Z* conformation adopts a helical conformation. (e) The ^1^H NMR spectrum of compound 1 exhibit distinct changes in response to light exposure and dark storage duration, reflecting the interconversion between the *E* and *Z* configurations. The relative ratio of these two configurations can be quantitatively determined based on ^1^H NMR (in CDCl_3_, 3.0 mM). (f) Δ*G* values of the *E* and *Z* configurations of compound 1. (The geometric structures of compound 1 were optimized at M06-2X-D3(0)/def-TZVP level of theory with Gaussian 16.)

The absolute configuration of compound 1 was determined by X-ray diffraction analysis. A single crystal suitable for analysis was obtained through the slow evaporation of a dichloromethane solution, revealing that the entire molecule adopts a stable *E* conformation, with all aromatic rings being approximately coplanar. However, the terminal pyridine rings display slight deviations from planarity, folding in opposite directions relative to the molecular plane. The molecular structure features four sets of pairwise symmetric three-center hydrogen bonds. Notably, the two sets located in close proximity to the azo group exhibit greater strength, as evidenced by shorter N⋯H distances (0.189 nm and 0.222 nm), which significantly contribute to the observed coplanarity of the adjacent aromatic systems. In contrast, the two sets of hydrogen bonds adjacent to the pyridine moieties are weaker, with longer N⋯H distances (0.246 nm and 0.205 nm), a feature attributed to the nonplanar distortion of the terminal pyridine groups that compromises optimal hydrogen bond geometry (Fig. 1a and b).

The photoresponsive properties of compound 1 were subsequently investigated. Upon alternating exposure to light and dark conditions, *E* configuration can be reversibly converted into the *Z* configuration. The signal changes in the ^1^H NMR spectra revealed that the *E* configuration of compound 1 transformed into a *Z* configuration upon UV irradiation (365 nm) (Fig. 1e). Moreover, the presence of intramolecular three-center HBs promoted the formation of a helical structure (Fig. 1d). Indirect evidence for the persistence of intramolecular HBs in solution was provided by ROE NMR, which showed no significant cross-peaks between the amide N–H protons and adjacent aromatic ring protons (Fig. S36 and S37, SI). After 15 minutes of UV irradiation, the ratio of the *E* configuration to the *Z* configuration reached 40 : 60. Extending the irradiation time to 30 minutes resulted in a ratio of 38.5 : 61.5, while further prolongation had minimal impact on the isomer distribution (60 min: 38.5 : 61.5; 120 min: 35.7 : 64.3). ^1^H NMR-based photoisomerization experiments revealed that for compound 1, the photostationary state (PSS)^46–48^ was essentially reached within 15 min upon irradiation at 365 nm, and only a slight increase in the *Z* configuration content was observed after 120 min of irradiation. After the sample was transferred to the dark, the *Z* configuration was reversibly transformed into the *E* configuration within 3 hours. The dark-state isomerization revealed that the PSS of compound 1 under dark was dominated by the *E* configuration, demonstrating its considerably higher stability relative to the *Z* configuration. This observation was corroborated by additional density functional theory (DFT) calculations, which revealed a Gibbs free energy difference (Δ*G*) of 15.8 kcal mol^−1^ between the *E* and *Z* conformations (Fig. 1c and f). Collectively, these results illustrate that azo units can be effectively combined with noncovalent interactions to construct a photoresponsive helical structure. This strategy enables the modulation of intermolecular interactions and provides a supplementary design perspective for the construction of light-driven functional supramolecular materials.

**Fig. 2:**
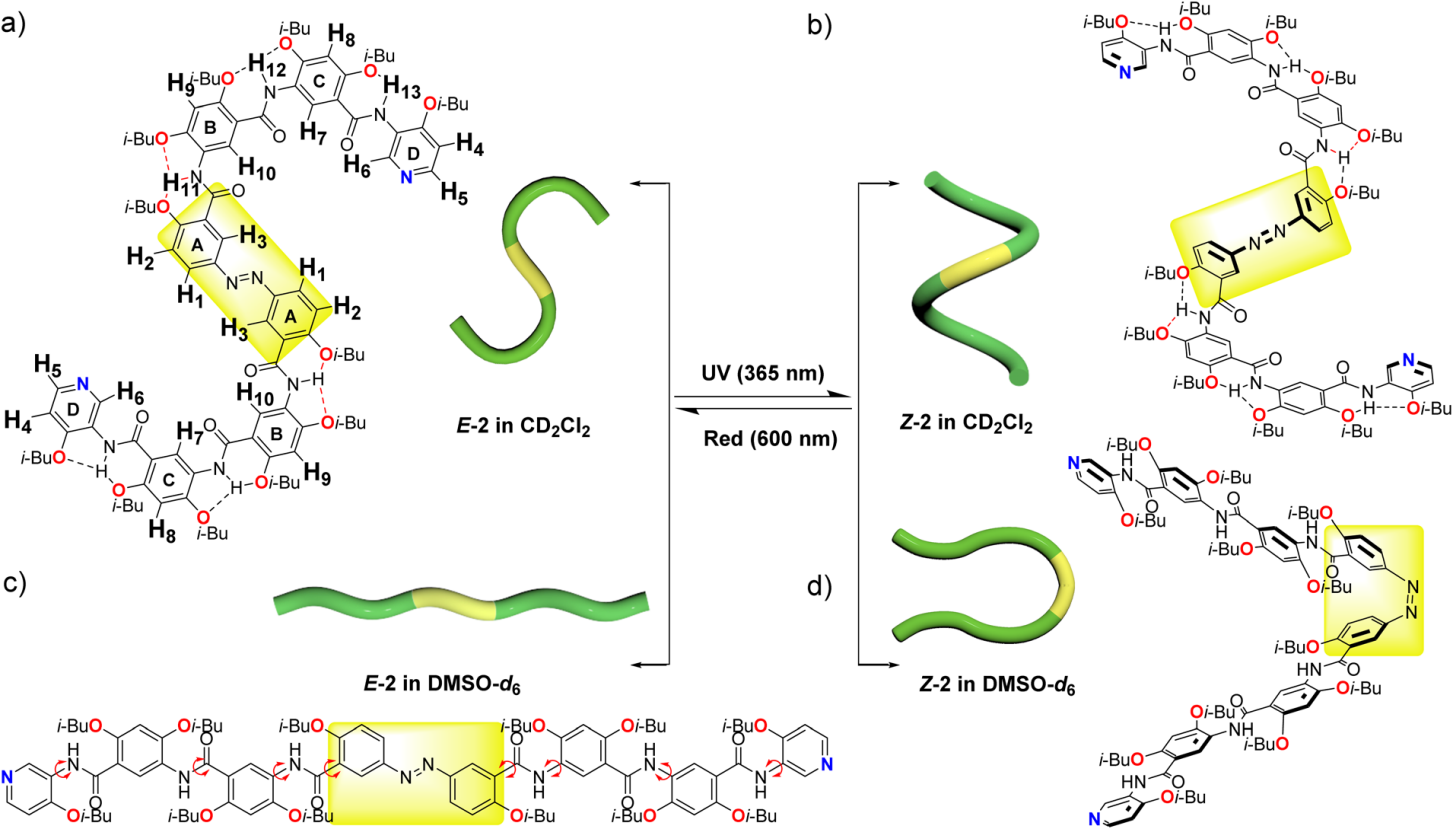
(a) Molecular structure and schematic diagram of compound 2 in the *E* configuration in CD_2_Cl_2_, featuring intramolecular three-center hydrogen bonding (all the hydrogen atoms in the molecule were systematically numbered and highlighted). (b) The molecular structure and schematic diagram of compound 2 in the *Z* configuration in CD_2_Cl_2_ (5.0 mM), featuring intramolecular three-center hydrogen bonding. (c) Molecular structure and schematic diagram of compound 2 in the *E* configuration in DMSO-*d*_6_. (d) Molecular structure and schematic diagram of compound 2 in the *Z* configuration in DMSO-*d*_6_ (5.0 mM).

**Fig. 3:**
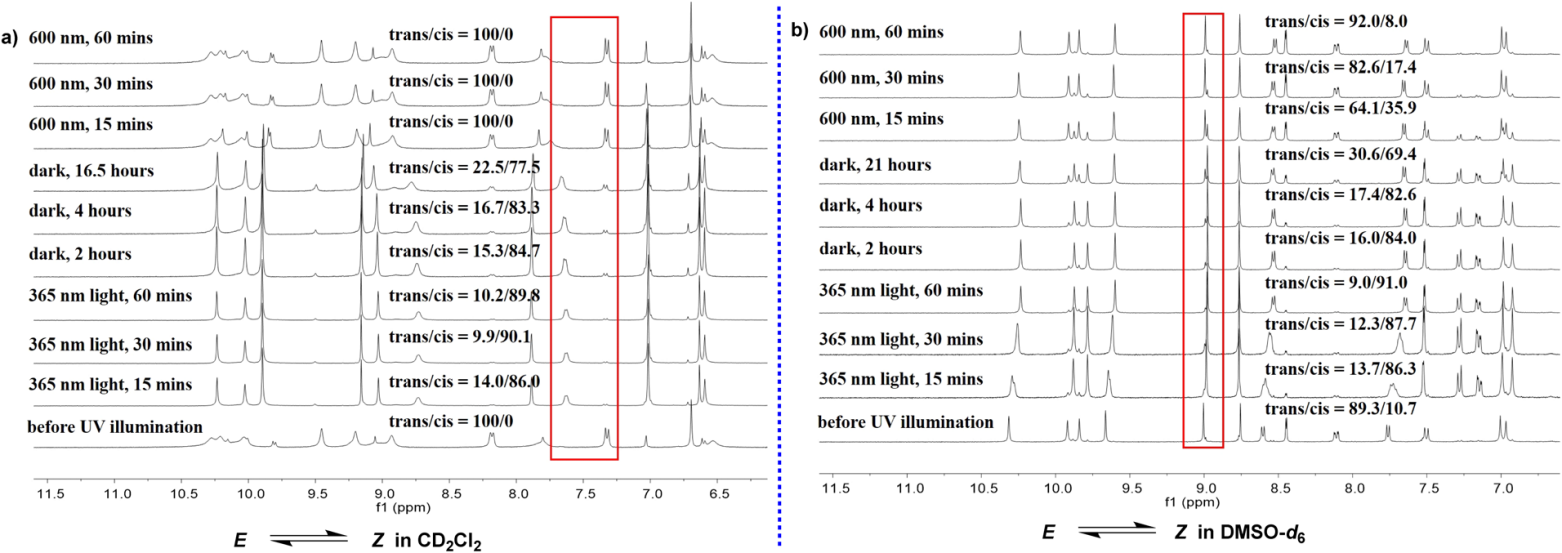
The ^1^H NMR spectrum of compound 2 shows distinct changes in response to light exposure and duration of dark storage, reflecting the interconversion between the *E* and *Z* configurations. The relative ratio of these two configurations can be quantitatively determined based on ^1^H NMR in CD_2_Cl_2_ (a) (5.0 mM) and DMSO-*d*_6_ (b) (5.0 mM).

Following the preliminary photoresponse studies on model molecular compound 1, compound 2 was further investigated, which features an extended arylamide backbone. Owing to the distinctive behavior of intramolecular hydrogen bonds, H-bonded arylamide foldamers can adopt either crescent or linear conformations.^49^ In this work, the conformational interconversion of compound 2 was modulated by tuning two key factors: the solvent environment and light irradiation (Fig. 2). First, the photoresponsive properties of compound 2 were systematically examined in deuterated dichloromethane (DCM-*d*_2_) and deuterated dimethyl sulfoxide (DMSO-*d*_6_) using one-dimensional (1D) and two-dimensional (2D) ^1^H NMR. DCM-*d*_2_ was selected as the primary solvent—replacing the CDCl_3_ used for compound 1—due to the enhanced solubility and stability of compound 2 in this medium. In DCM-*d*_2_, compound 2 initially adopted a stable *E* conformation. Upon irradiation with 365 nm UV light for 15 minutes, the *E*/*Z* ratio shifted to 14 : 86 (Fig. 2a, b, 3a and S38a, SI). Extending the irradiation time to 30 minutes further altered the ratio to 9.9 : 90.1, whereas prolonged exposure (60 minutes) resulted in no significant improvement (10.2 : 89.8), indicating that the PSS have been reached (Table 1). Notably, owing to the discontinuous nature of the ^1^H NMR measurements, potential conformational changes may have occurred between sample acquisitions. Compared with compound 1, which exhibited a maximum *Z* population of 64.3% under UV irradiation, compound 2 achieved a significantly higher *Z* content of up to 90.1%. Moreover, the *Z* conformation of compound 2 demonstrated notable kinetic stability in the dark. After 2 hours in darkness, the *Z*/*E* ratio remained at 84.7 : 15.3, and only gradual relaxation was observed over time (83.3 : 16.7 for 4 hours and 77.5 : 22.5 for 16.5 hours), suggesting slow thermal reversion. These results indicated the low efficiency of the dark-mediated *Z* → *E* conversion. In contrast, irradiation with 600 nm red light dramatically accelerated the recovery process, resulting in complete reversion to the *E* conformation within 15 minutes (Fig. 3a, Table 1 and Fig. S38a, SI). 600 nm light was chosen to selectively drive *Z* → *E* isomerization, based on the red-shifted n → π* absorption of the *Z* conformation and the mild, non-damaging nature of long-wavelength excitation.^50^ In DMSO-*d*_6_, irradiation with 365 nm UV light for 60 minutes yielded a high *Z* fraction of 91%, with improved peak resolution compared with that observed in DCM-*d*_2_. Under dark conditions, the *Z* conformation gradually reverted to the *E* form, decreasing to 69.4% after 21 hours. Compared with exposure to DCM-*d*_2_, exposure to 600 nm red light promoted this reversion, albeit with a lower efficiency. After 60 minutes of red light irradiation, the *E* content increased to 92% (Fig. 2c, d, 3b and S38b, SI).

**Table 1:** *E* ⇄ *Z* photoisomerization properties of compound 2 and 3

	Photoconversion^a^ (%)		Quantum yield^b^ (*Φ*, %)		Rate constant^c^ (*k*_photo_, s^−1^)		Half-life^c^ (*t*_1/2_)	
	*E* (PSS)	*Z* (PSS)	*E* → *Z*	*Z* → *E*	*E* → *Z*	*Z* → *E*	*E* → *Z*	*Z* → *E*
2	100^d^	90.1^d^	5.09^h^	9.31^h^	3.3 × 10^−3^	>4.62 × 10^−3^^m^	3.5 min	<2.5 min^m^
	92.0^e^	87.7^e^	3.07^i^	7.24^i^	2.20 × 10^−3^	6.10 × 10^−4^	5.3 min	19.0 min
3	100^f^	72.2^f^	1.78^j^	3.21^j^	3.90 × 10^−4^	1.55 × 10^−5^^l^	29.6 min	12.4 h^l^
	100^g^	66.1^g^	0.68^k^	5.94^k^	3.15 × 10^−4^	1.01 × 10^−3^	36.7 min	11.4 min

a The percentages of *E* and *Z* configurations at photostationary state (PSS) were calculated from the ^1^H NMR data (Fig. 3a, b and 7a, b).

b The quantum yields were determined in different selected solvents. The sample concentrations of compound 2 and 3 were all 4.4 × 10^−6^ mol L^−1^. The wavelength of the light for the *E* → *Z* conversion was 365 nm. The wavelength of the light for the *Z* → *E* conversion was 600 nm.

c The *E* ⇄ *Z* isomerization kinetic rate constants (*k*_photo_) and half-life (*t*_1/2_) were calculated based on the ^1^H NMR data of the photoisomerization of compound 2 and 3 (Fig. 3a, b and 7a, b). The specific calculation method can be found in Section 15 of SI.

d CD_2_Cl_2_.

e DMSO-*d*_6_.

f CDCl_3_.

g CDCl_3_/DMSO-*d*_6_ (3 : 1 V/V).

h CH_2_Cl_2_.

i DMSO (Fig. S56a and S56b, SI).

j CHCl_3_.

k CHCl_3_/DMSO (3 : 1 V/V) (Fig. S56c and S56d, SI).

l Complete *Z* → *E* isomerization was attained within 6 hours under dark conditions, and the thermal relaxation rate constant (*k*_thermal_) for *Z* → *E* isomerization can be derived.

m Complete *Z* → *E* isomerization was attained within just 15 minutes under 600 nm irradiation, precluding the higher *k*_photo_ and shorter *t*_1/2_.

**Fig. 4:**
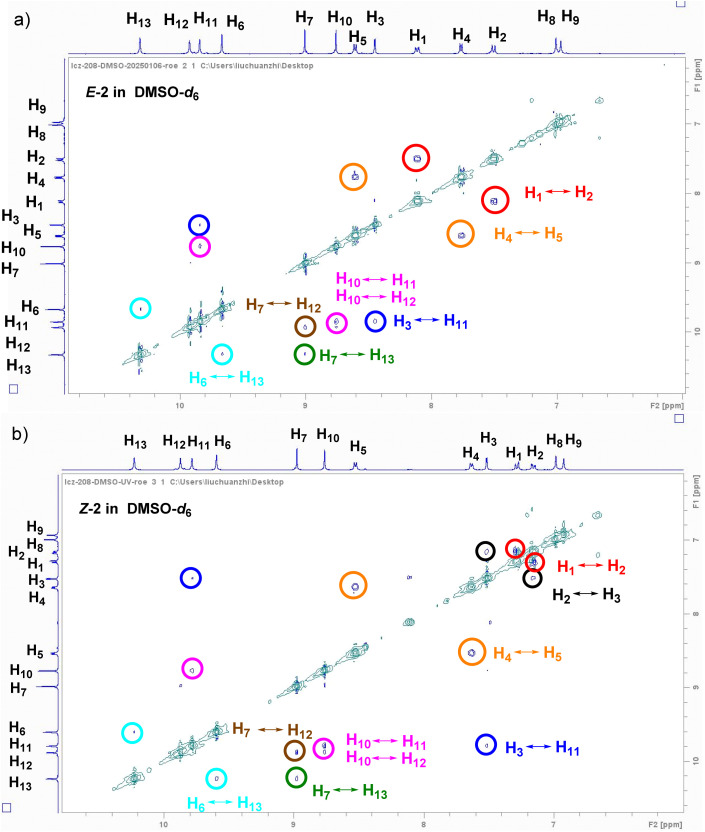
2D ROE spectra of the *E* (a) and *Z* (b) configurations of compound 2 in DMSO-*d*_6_ (5.0 mM). (The signals corresponding to the same group of hydrogen atoms are indicated by circles of identical color.)

Afterward, 2D ROE and NOE NMR were employed to elucidate the behavior of intramolecular hydrogen bonds in both the *E* and *Z* configurations in DCM-*d*_2_. In the ROE spectra of the *E* configuration, the resolution of the 1D ^1^H NMR spectrum was partially compromised by intermolecular stacking, while the correlation signals of H_1_ and H_2_ from the azo fragment remained clearly discernible (Fig. S39, SI). In addition, no other significant cross-peaks were detected, supporting the formation of an S-shaped conformation (Fig. 2a). Following UV-induced isomerization to the *Z* configuration, the ROE and NOE spectra similarly revealed an absence of correlation signal between the amide hydrogen atoms and aromatic hydrogen atoms, indicating the presence of intramolecular three-center hydrogen bonding. Consequently, the entire molecule adopted a helical structure (Fig. 2b and S40, SI). Unlike dichloromethane, DMSO belongs to a category of highly polar and hydrogen-bonding competitive solvents.^51^ Its high solubility diminished the intermolecular stacking effect, thereby enhancing the resolution of the 1D ^1^H NMR of compound 2. Additionally, the solvent effect disrupted the intramolecular three-center hydrogen bonding pattern, facilitating free rotation of the amide bonds in the *E* configuration and leading to a linear conformation of the molecule (Fig. 2c). The ROE spectrum of the *E* configuration in DMSO-*d*_6_ revealed multiple sets of correlation signals, categorized by colored circles for clarity. These correlation signals involved not only adjacent hydrogen atoms within the same aromatic ring, such as H_1_ and H_2_ (red circle) and H_3_ and H_4_ (orange circle) but also H_10_ and H_11_/H_12_ (pink circle), H_3_ and H_11_ (blue circle), H_7_ and H_12_ (brown circle), H_7_ and H_13_ (green circle), and H_6_ and H_13_ (light blue circle), resulting from amide bond rotation after the disruption of three-center hydrogen bonding (Fig. 4a). The ROE spectra of the *Z* configuration revealed analogous correlation signals, with a notable distinction: the emergence of an additional set of correlation signals between H_2_ and H_3_ (black circle) (Fig. 4b). This phenomenon can be ascribed to the diminished distances of hydrogen atoms engendered by the *Z* structural arrangement. Integration of key cross-peaks of *E* and *Z* configurations of compound 2 in DMSO-*d*_6_ was shown in Table S1. By optimizing the solvent and light wavelength in conjunction with the 2D NMR approach, four distinct molecular morphologies of compound 2 were elucidated (S-shaped, helical, linear, and U-shaped structures) (Fig. 2). Furthermore, reversible photoinduced transformations between the S-shaped and helical structures, as well as the linear and U-shaped configurations, were successfully achieved. These findings elucidate a molecular-level mechanical system and provide valuable insights for the development of functional materials responsive to both solvents and light.

**Fig. 5:**
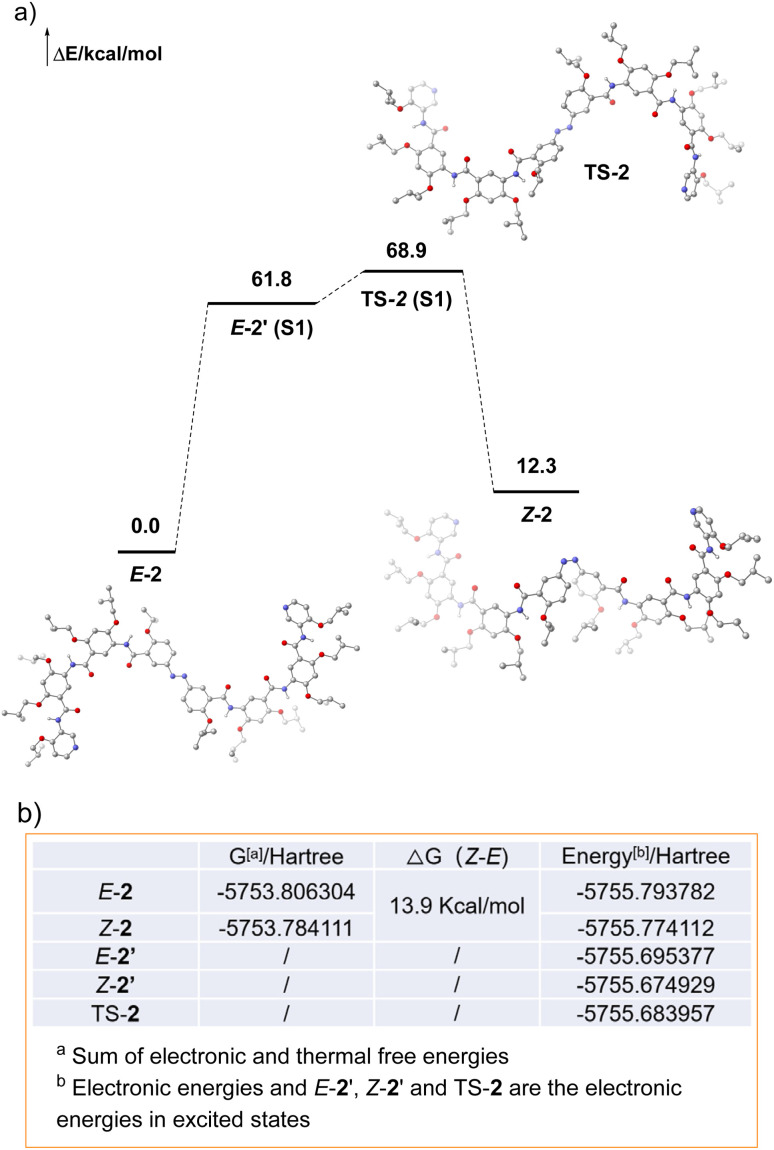
(a) Schematic potential energy profile for the *E* → *Z* photoisomerization in the ground (S_0_) and excited (S_1_) states. (b) Calculated free energies (*G*) and electronic energies (energy) of *E*/*Z* conformations in DCM. (The geometric structure of compound 2 was optimized at B3LYP-D3(BJ)/6-311G** level of theory using IEFPCM solvation model in dichloromethane. The vertical electronic excitation energies of the *E*, *Z* conformations and transition states were calculated using TD-DFT, and five singlet excited states were considered at B3LYP-D3(BJ)/6-311G** level of theory with IEFPCM solvation model in dichloromethane.^52^)

Furthermore, DFT and time-dependent density functional theory (TD-DFT)-based theoretical modeling were employed to investigate the influence of solvents and light irradiation on the conformational behavior of compound 2, complementing experimental observations obtained beyond NMR. In dichloromethane, the ground-state (S_0_) potential energy surface (PES) of compound 2 demonstrated that the *E* conformation was the thermodynamically predominant conformation, which was set as the energy reference (0.0 kcal mol^−1^). The *Z* conformation possessed an energy of 12.3 kcal mol^−1^, corresponding to the electronic potential energy difference (Δ*E*) between the two geometric isomers and directly indicating the relative instability of the *Z* configuration at the intrinsic electronic level. Upon photoexcitation to the first excited state (S_1_), the *E* conformation was populated to its S_1_ counterpart (*E*-2′, 61.8 kcal mol^−1^) upon light irradiation. Subsequent isomerization proceeded *via* surmounting a moderate energy barrier of 7.1 kcal mol^−1^ to reach the S_1_ transition state TS-2 (68.9 kcal mol^−1^), followed by relaxation to the ground-state *Z*-2 (S_0_) to accomplish efficient *E*-to-*Z* photoisomerization (Fig. 5a). This photoinduced process, coupled with the ground-state thermodynamic bias, constitutes the core mechanism underlying the molecular photoresponsive behavior: the *E* conformation was thermodynamically favored in the S_0_ state, whereas light irradiation triggers its conversion to the *Z* conformation, enabling reversible photocontrol. Further analysis of the computed free energy data revealed that the free energy difference (Δ*G*(*Z*–*E*)) between the *Z* and *E* conformations in DCM was 13.9 kcal mol^−1^. This value integrated both electronic energy and thermal free energy contributions (including translational, rotational, and vibrational entropic terms), thereby offering a more accurate depiction of the thermodynamic behavior under realistic chemical conditions. The minor discrepancy between Δ*G* (13.9 kcal mol^−1^) and Δ*E* (12.3 kcal mol^−1^) underscored the non-negligible impact of thermal corrections on molecular stability (Fig. 5b). Specifically, in DCM, the *Z* conformation was not only electronically less stable but also significantly destabilized in terms of total free energy—consistent with the PES results and collectively validating the absolute thermodynamic dominance of the *E* conformation in the ground state. Additionally, we have also performed density DFT calculations to determine the Δ*G* and Δ*E* associated with the photoisomerization of compound 2 in DMSO (Fig. S60, S61 and Table S9, SI). Collectively, these computational results not only corroborate the previous experimental findings but also provide mechanistic insight into the four distinct conformational states of compound 2 under varying solvent and photoirradiation conditions.

**Fig. 6:**
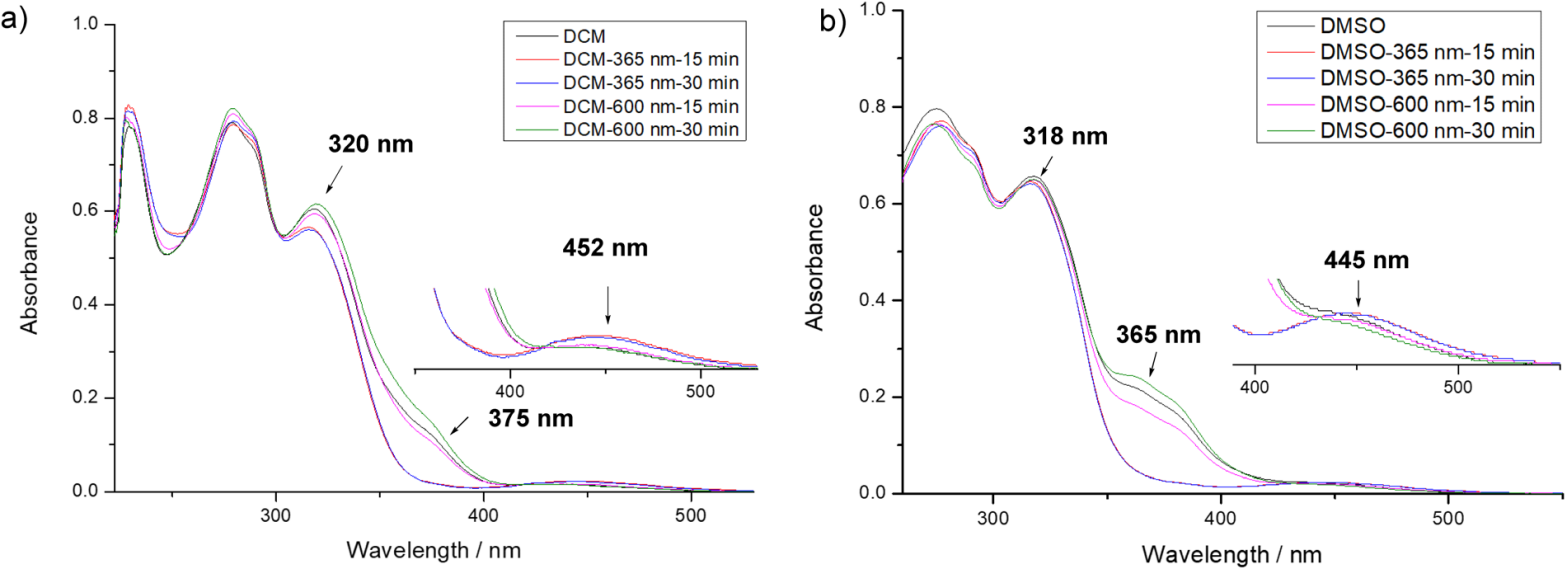
UV-vis absorption spectra of compound 2 under various irradiation durations at 365 nm and 600 nm. (a) The spectra when dichloromethane was used as the solvent. (b) The spectra when DMSO was used as the solvent. (The regions at 400 nm and 500 nm have been specifically magnified for enhanced clarity. The sample concentrations of compound 2 in DCM and DMSO were all 1.1 × 10^−6^ mol L^−1^.)

To gain more insight into the mutual conversion of the *E* and *Z* configurations, UV-vis spectroscopy experiments were performed to monitor the photoresponsive behavior in both DCM and DMSO. In DCM, upon increasing the irradiation time at 365 nm from 15 to 30 minutes, the absorption intensity at wavelengths of 320 nm and 375 nm significantly decreased, while a concomitant increase was observed at approximately 452 nm. However, when the light was changed to 600 nm red for irradiation, the absorption peaks at the two previously reduced positions returned to their initial state. A similar trend was observed in DMSO, albeit with slight shifts in the absorption maxima (Fig. 6). Upon 365 nm UV irradiation, the azo linkages underwent *E*-to-*Z* isomerization. The distorted *Z* conformation disrupted the native hydrogen-bonding network and π–π stacking interactions, resulting in diminished absorption intensity in the UV region. Meanwhile, the *Z* conformation exhibited a distinct n → π* transition, giving rise to a prominent increase in absorbance within the 400–500 nm range.^53^ UV-vis spectroscopy experiments, supported by robust data, further substantiated the significant property of photoinduced isomerization between the *E* and *Z* configurations of compound 2.

An alternating irradiation protocol with 365 nm UV and 600 nm red light was employed to investigate the reversible *E* ⇄ *Z* isomerization of compound 2. After more than 30 repeated irradiation cycles (over 53 hours of UV and 53 hours of red light exposure in total), the sample retained its initial NMR purity in both DCM-*d*_2_ and DMSO-*d*_6_ (Fig. S41–S47, SI). These results confirmed the favorable fatigue resistance and structural stability of compound 2 under alternating light irradiation, supporting its potential application in optically driven functional materials. While azo-fused H-bonded arylamide foldamer 2 exhibits both solvent- and photoresponsive conformational switching, its functionality remains confined to unimolecular configurational transformations, with no capacity for further intermolecular assembly. Compound 3 was systematically investigated in subsequent research, which incorporates ChB-based donor–acceptor terminal segments designed to enable intermolecular assembly under photostimulation. Compounds 1 and 2 adopt a *Z* configuration with a helical conformation upon exposure to 365 nm UV light in chloroform or DCM. Although compound 3 shares structural similarities with compounds 1 and 2, its molecular length restricts the unimolecular helix to a relatively short segment (Fig. 9a). Theoretically, intermolecular Se⋯N noncovalent interactions are anticipated to guide the further assembly of monomer molecules, thereby facilitating the formation of long-range ordered supramolecular helices.

**Fig. 7:**
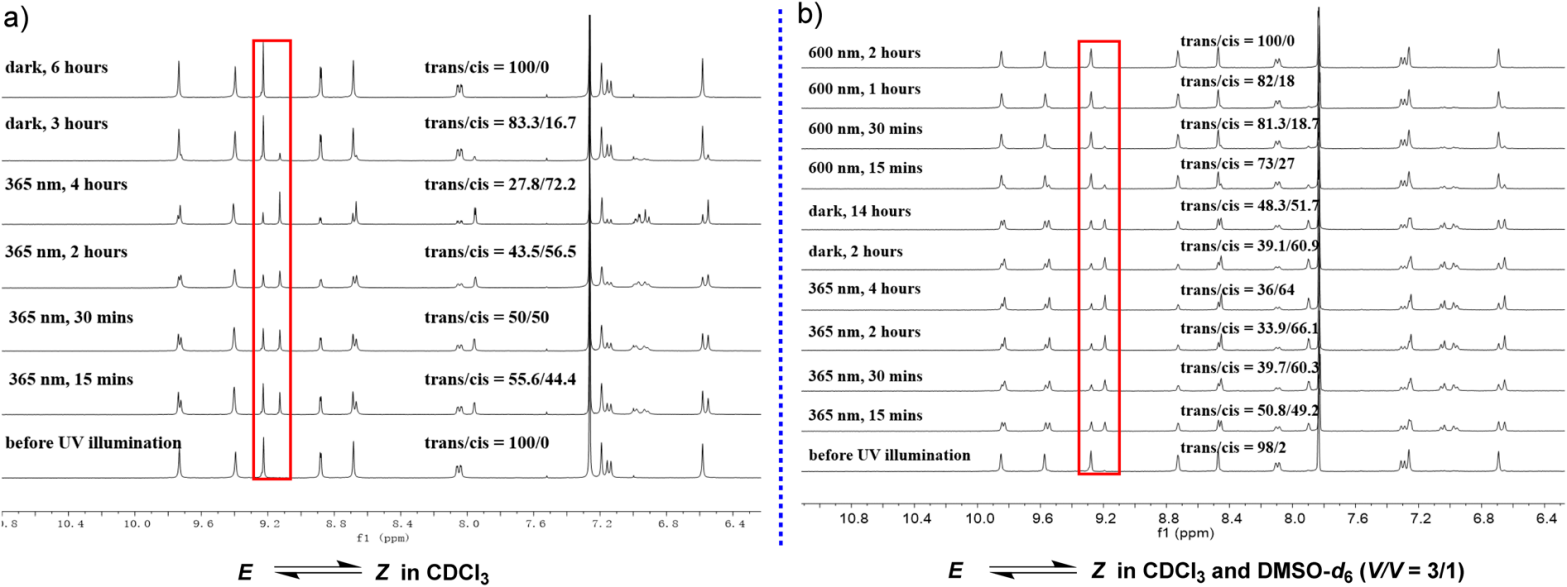
The ^1^H NMR spectrum of compound 3 shows distinct changes in response to light exposure and duration of dark storage, reflecting the interconversion between the *E* and *Z* configurations. The relative ratio of these two configurations can be quantitatively determined based on ^1^H NMR in CDCl_3_ (5.0 mM) (a) and a mixed solvent of CDCl_3_/DMSO-*d*_6_ at a volume ratio of 3/1 (b) (5.0 mM).

Following the established experimental approach, the photoresponse of compound 3 in chloroform was initially investigated using ^1^H NMR. Chloroform was selected as the solvent because of the limited solubility of compound 3 in DCM. Prior to UV irradiation, the compound existed exclusively in the *E* configuration. Upon exposure to 365 nm UV light for 15 minutes, the *E* to *Z* ratio was determined to be 55.6 : 44.4. After an additional 30 minutes of irradiation, this ratio reached a PSS of approximately 50 : 50, with further irradiation up to 4 hours resulting in a modest increase to 27.8 : 72.2. Under dark conditions, the ratio gradually reverted to 83.3 : 16.7 within 3 hours, with complete thermal reversion to the *E* configuration observed after 6 hours (Fig. 7a, 9a and Table 1). In a mixed solvent system of CDCl_3_/DMSO-*d*_6_ (3 : 1 V/V), as the sample exhibited poor solubility in pure DMSO-*d*_6_, irradiation at 365 nm for 30 minutes yielded a *Z* configuration content of 60.3%. Extending the irradiation duration to 2 hours increased the percentage of *Z* type compounds slightly to 66.1%, with no further significant change observed upon extending the duration to 4 hours (Fig. 7b, 9a and Table 1). Under dark storage conditions, a gradual reversion from the *Z* to the *E* configuration was confirmed, albeit substantially slower than in pure CDCl_3_. Specifically, after 14 hours in the dark, the *E* configuration accounted for only 48.3% of the mixture. Notably, irradiation with 600 nm red light effectively accelerated the reversion to the *E* configuration, achieving complete conversion within 2 hours—a behavior consistent with that observed for compound 2 under identical solvent conditions. Nevertheless, a pronounced difference in the *Z* configuration fraction at the PSS was observed between compounds 2 and 3 (Table 1), which originated from the distinct conformational constraints imposed by their oligomeric chain lengths. In compound 2, the extended backbone imposes increased steric hindrance around the azo unit, limiting its rotational freedom and thus favoring a higher *Z* configuration population at the PSS. By contrast, the shorter compound 3 features enhanced flexibility of its arylamide backbone, enabling more efficient *Z* → *E* isomerization. This trend was retained in solvents of both low and high polarity, highlighting that oligomer length and conformational restriction are decisive factors in modulating the PSS composition of photoresponsive foldamers.

**Fig. 8:**
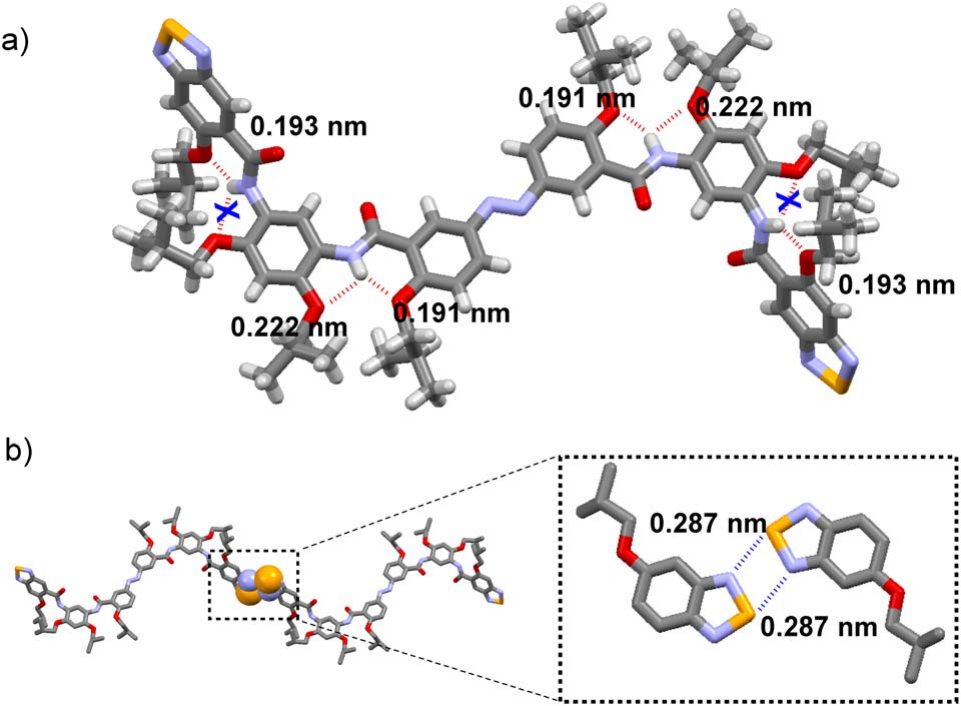
(a) Crystal structure of compound 3. The bond length data of intramolecular three-center hydrogen bonds. (b) The Se⋯N interaction mechanism between molecules and the corresponding bond lengths (H atoms were omitted for clarity for (b)).

**Fig. 9:**
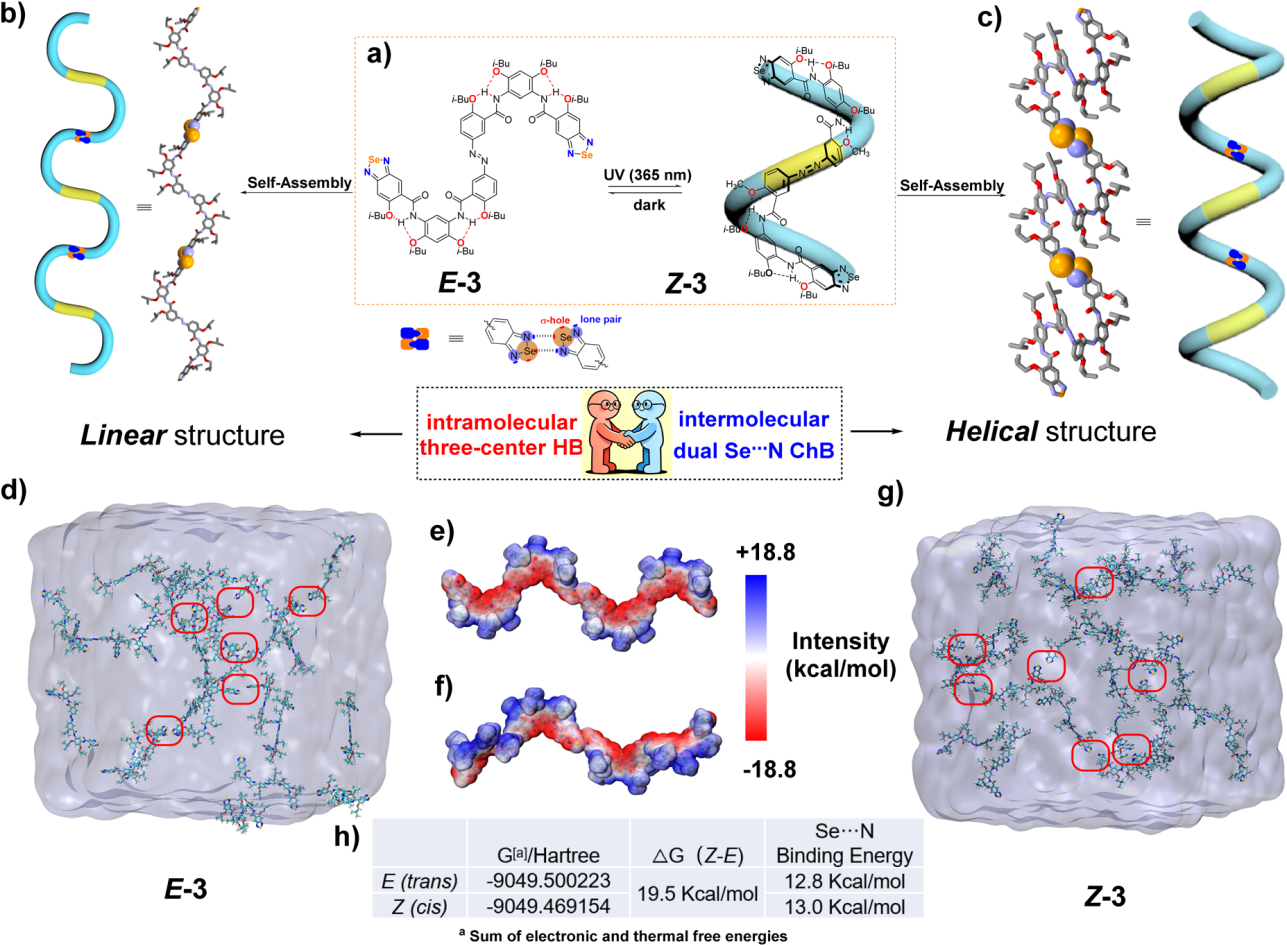
(a) Photoresponsive interconversion between the *E* and *Z* configurations of compound 3, along with a schematic representation of the helical structure in the *Z* configuration. (b) Planar curved self-assembled structure in the crystal structure induced by intermolecular Se⋯N interactions in the *E* configuration. (c) Computer-simulated optimized supramolecular helix induced by intermolecular Se⋯N interactions in the *Z* configuration. (d) Self-assembly behavior guided by Se⋯N noncovalent interactions between *E* configuration in chloroform through molecular dynamics (MD) simulations. (e) and (f) The electrostatic potential and Gibbs free energy of the *E* and *Z* configurations of compound 3, as well as the bond energy of the Se⋯N ChB. (The obtained intermediates were fully optimized at the DFT level using the M062X-D3 and B3LYP functional.) (g) Self-assembly behavior guided by Se⋯N noncovalent interactions between the *Z* configuration in chloroform through molecular dynamics (MD) simulations. (h) Δ*G* values of the *E* and *Z* configurations of compound 3, as well as Se⋯N bond energy. (H atoms are omitted for clarity for (b) and (c). The intermolecular Se⋯N interactions are indicated within the red circle for (d) and (g). The geometric structures of compound 3 were optimized at M06-2X-D3(0)/def-TZVP level of theory with Gaussian 16. MD simulations: the molecules were optimized at the B3LYP level using the 6-31+G(d,p) basis set with Gaussian 16 program, where the solvent effects were corrected by applying the SDM method. The RESP charges were further optimized alongside the wave function using Multiwfn.)

UV-vis spectroscopy was subsequently performed to further characterize the photoresponsive behavior associated with the *Z* ⇄ *E* isomerization of compound 3 (Fig. S48, SI). And the experimental spectra were compared with TD-DFT calculations (Fig. S59, SI). Both UV-vis spectroscopic measurements and TD-DFT calculations demonstrated that the absorption characteristics of compound 3 are not significantly sensitive to the *E*/*Z* isomerization, in contrast to the behavior observed for compound 2.

Kinetic analysis based on ^1^H NMR revealed distinct isomerization rates and relative stabilities. Compounds 2 and 3 consistently exhibited larger *k*_photo_ for *Z* → *E* isomerization than for *E* → *Z* photoisomerization in both low polar (CD_2_Cl_2_, CDCl_3_) and polar (DMSO-*d*_6_, CDCl_3_/DMSO-*d*_6_ = 3 : 1, V/V) solvents. In nonpolar environments, compound 2 exhibited the fastest response kinetics, reflecting a lower activation barrier for *E* → *Z* conversion in apolar media, which was in agreement with TSs energy barrier of compound 2 (Fig. 5). In contrast, compound 2 displayed markedly faster photoswitching kinetics than compound 3, with the *Z* → *E* photoconversion reaching full completion within minutes (Tables 1, S4 and S7, SI). Since a longer *t*_1/2_ or a lower *k*_photo_ indicated greater resistance to isomerization, this data definitively confirmed that the *E*-configuration was the thermodynamically stable configuration. The enrichment of the *Z*-configuration at the PSS was therefore a direct consequence of kinetic selection *via* photoexcitation, rather than a reflection of thermal stability. On the other hand, quantum yield measurements provided further insights into the photochemical conversion efficiency. For both compounds 2 and 3, the quantum yields of *Z* → *E* photoisomerization were generally higher than the corresponding *E* → *Z* photoisomerization (compounds 2, *Φ*: 9.31%, 7.24% for *Z* → *E*, 5.09%, 3.07% for *E* → *Z*; compounds 3, *Φ*: 3.21%, 5.94% for *Z* → *E*, 1.78%, 0.68% for *E* → *Z*) (Fig. S57 and S58, SI), a typical feature of azobenzene-based photoswitches. This discrepancy primarily arose from the thermodynamic favorability of *Z* → *E* isomerization, as the *E*-configuration was thermodynamically more stable than the *Z*-configuration, leading to a lower activation energy barrier for the reverse process. Additionally, the *Z*-configuration exhibited fewer non-radiative deactivation pathways upon photoexcitation compared to the *E*-configuration, thereby enabling more efficient conversion to the *E* configuration. This characteristic was critical for maintaining the PSS of the two compounds under continuous illumination, ensuring the establishment of a stable isomer equilibrium.^48,53^ Overall, compound 2 exhibited higher quantum yields than compound 3 across all conditions. This observation can be rationalized by the heavy-atom effect introduced by Se in compound 3, which might result in a fluorescence quenching by increased probability of intersystem crossing, and consequently lowers its quantum yield.^54–56^

The single-crystal structure of compound 3 was successfully obtained by low evaporation of a chloroform solution, enabling unambiguous elucidation of its intermolecular packing and arrangement in the *E* configuration. Surprisingly, not all the hydrogen atoms on the intramolecular amide nitrogen participated in the formation of three-center hydrogen bonding. Instead, the three-center hydrogen bonding adjacent to the benzoselenadiazole terminal was sacrificed to facilitate a nearly planar zigzag arrangement between molecules, driven by dual Se⋯N interactions (bond length: 2.87 Å and 2.99 Å; the sum of the van der Waals radius of Se and N: 3.45 Å) (Fig. 8 and 9b). Owing to the inherent instability of the *Z* configuration, its structural characterization *via* single-crystal X-ray diffraction proved challenging. As a result, its assembly in the solid-state was explored through X-ray powder diffraction analysis (Fig. S53 and S54, SI). A solid sample was obtained by gradual solvent evaporation from a DCM solution under continuous UV irradiation (365 nm). Concurrently, the helical structure guided by the Se⋯N ChB was simulated based on the *E* configuration of the intermolecular ChB bond length and the calculation of the optimized *Z* monomer structure (Fig. 9c). The PXRD pattern derived from this simulation was compared with the actual measured pattern. The refinement yielded a weighted profile *R*-factor (*R*_wp_) of 4.19%, indicating a good fit between the simulated and experimental diffraction patterns. All major diffraction peaks in the experimental pattern aligned precisely with those calculated from the model, confirming the accuracy of the unit cell parameters and crystal lattice structure. The relative intensities of the peaks are well-reproduced, with minor deviations observed in the difference plot attributed to minor structural disorder and preferred orientation effects inherent to powder samples. These results demonstrated that the proposed packing model accurately reflected the long-range molecular arrangement in the solid state. Furthermore, ^1^H NMR spectra acquired from the re-dissolved sample afforded a *Z*-to-*E* ratio of 55 : 45 (Fig. S55, SI), thereby confirming that the *Z* configuration dominates in the as-prepared powder.

The topology of the *E* and *Z* configurations in the solid-state was confirmed, and the organization of monomers in the solution phase became the focus of our subsequent investigations. First, the presence of intramolecular three-center hydrogen bonding in both the *E* and *Z* configurations was verified in a CDCl_3_ solution through ROE experiments. Additionally, photoirradiation experiments conducted in mixed CDCl_3_/DMSO-*d*_6_ (3 : 1 V/V) verified the coexistence of both linear and U-shaped conformations (Fig. S49–S52, SI). Afterward, the self-assembly behavior of *E* and *Z* monomers in chloroform was investigated using molecular dynamics (MD) simulations. In a closed system containing *E* monomers and chloroform, the monomers gradually aggregated over time because of intermolecular Se⋯N interactions, with multiple interaction sites emerging after 20 ns. A similar trend was observed in the system with *Z* monomers, demonstrating that both configurations exhibit a propensity for spontaneous assembly in solution, primarily driven by intermolecular ChB (Fig. 9d and g). Furthermore, electrostatic potential (ESP) analysis and DFT calculations were performed to evaluate the intermolecular binding energies and elucidate the electrostatic characteristics of the *Z* and *E* configurations (Fig. 9e and f). The simulation results indicated that both the *Z* and *E* configurations exhibited distinct electrostatic states characterized by favorable electrostatic attraction in intermolecular interactions. DFT calculations indicated that the energy of the *Z* configuration is greater than that of the *E* configuration, with a Δ*G* value of 19.5 kcal mol^−1^. Notably, both configurations exhibit strong intermolecular binding energies, with values of 13.0 kcal mol^−1^ (*Z*) and 12.8 kcal mol^−1^ (*E*). Notably, these findings were obtained under the constraint of using chloroform as the solvent. In order to further confirm the existence of ChB, geometric optimization of the dimers formed by the *Z* and *E* conformations of compound 3 was performed. Subsequently, an analysis of the weak interaction (IGMH analysis) between the two conformations was conducted. As shown in the Fig. S62, clear weak Se⋯N ChB interactions existed in both *Z* and *E* states of the dimer structures.

**Fig. 10:**
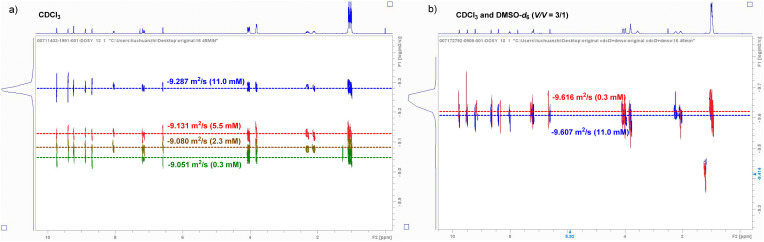
^1^H-DOSY NMR spectra of compound 3 at varying concentrations in CDCl_3_ (a) and in a mixed solvent of CDCl_3_/DMSO-*d*_6_ at a volume ratio of 3/1 (b).

Simultaneously, DOSY NMR was employed to indirectly elucidate the intermolecular Se⋯N interactions, focusing on a relatively stable *E* configuration. Variable concentration ^1^H DOSY diffusion experiments in chloroform confirmed the presence of several discrete species of different sizes, which were caused by monomer aggregation induced by intermolecular interactions. Under a series of concentration gradients, the *D* value exhibited a regular variation with changes in concentration. Specifically, a sample concentration of 11.0 mM corresponded to a log *D* value (diffusion coefficient value) of −9.287 m^2^ s^−1^, whereas concentrations of 5.5 mM, 2.3 mM, and 0.3 mM corresponded to log *D* values of −9.131 m^2^ s^−1^, −9.080 m^2^ s^−1^, and −9.051 m^2^ s^−1^, respectively (Fig. 10a). These data indicate that aggregation becomes more pronounced with increasing concentration. Conversely, as the concentration decreased, the intermolecular interactions progressively weakened. Notably, an eightfold difference at concentrations of 2.3 mM and 0.3 mM resulted in only a 0.029 change in the log *D* value, indicating that intermolecular interactions become exceedingly weak under these conditions. Nevertheless, when the solvent was composed of a CDCl_3_/DMSO-*d*_6_ mixture (3 : 1 V/V), the results from the DOSY experiment were notably different. The diffusion coefficients (log *D*) for both the 11.0 mM and 0.3 mM samples displayed minimal variation, indicating that intermolecular interactions within the CDCl_3_/DMSO-*d*_6_ solvent system were negligible (Fig. 10b). The DOSY experimental results demonstrated the impact of the solvent on intermolecular interactions, thereby indirectly corroborating the presence of such interactions within the chloroform system. Investigations on compound 3 revealed that Se possessed a larger atomic radius and was more polarizable, which significantly facilitated the formation of Se⋯N ChB. Meanwhile, the unique structural feature of the benzoselenadiazole moiety favored the formation of doubly reinforced intermolecular Se⋯N interactions. Driven by the orthogonal cooperation of intermolecular three-center HB, linear and helical supramolecular topologies were formed *via* self-assembly.

## Conclusions

In conclusion, a series of novel azo-fused H-bonded arylamide foldamers was successfully synthesized. This class of foldamers demonstrates remarkable photoresponsive characteristics, thereby facilitating reversible interconversion between the *E* and *Z* configurations. Under varying solvent and light environmental conditions, S-shaped, helix-shaped, linear-shaped, and U-shaped configurations were observed, resulting from the synergistic effects of the solvent-responsive behavior of intramolecular three-center hydrogen bonding and the intrinsic photoresponsive characteristics of azo moieties. Furthermore, these foldamers exhibited excellent mechanical robustness and photostability under repeated interconversions between the *E* and *Z* configurations. Notably, the incorporation of benzoselenadiazole termini endowed the monomer with enhanced self-assembly ability, facilitating the formation of enhanced dual Se⋯N ChB-driven supramolecular helices exhibiting photoresponsive features. Experimental results demonstrate that the synergistic integration of noncovalent interactions (including those between HBs and ChBs) with azobenzene-based photoresponsive units enabled the construction of helical supramolecular assemblies that respond dually to solvent polarity and light irradiation. This system achieved reversible unwinding-rewinding conformational transitions of H-bonded arylamide foldamers under specific solvent conditions and light stimuli, and the dynamic modulatory effects of intramolecular HBs and intermolecular ChBs on monomer assembly behaviors under external stimuli were thoroughly investigated.

Compared with the photoresponsive polymeric helical structures reported in previous literature, the helical architectures constructed in this work exhibit the following distinct characteristics: synthetic accessibility of the monomers, dual responsiveness to solvent polarity and light irradiation, versatile conformational transformability, synergistic assembly behavior mediated by non-covalent interactions (HB and ChB), as well as excellent photochemical stability and mechanical robustness. This study focused exclusively on the synergistic effects of HBs and ChB and did not address other potential noncovalent interactions (*e.g.*, hydrophobic effects, π–π stacking, and van der Waals forces). Although the H-bonded foldamers with a *Z* configuration exhibit lower thermodynamic stability and undergoes slow thermal isomerization to the *E*-form under dark conditions, this work successfully established a photoresponsive supramolecular helical architecture model. These findings provide novel insights for designing stimuli-responsive soft materials.

## Author contributions

CZL and BZ conceptualized the ideas and supervised the investigation. CZ, HSZ, SSH, SHG, MGZ and XYP performed the experiments and collected the data. LX and LXL performed the work related to computational calculations. CZL analysed the data. CZL wrote the original draft. CZL and BZ finished the writing. All authors read and contributed to the revisions.

## Conflicts of interest

The authors declare no conflict of interest.

## Supplementary Material

SC-OLF-D5SC10108E-s001

SC-OLF-D5SC10108E-s002

## Data Availability

The data supporting this article have been included as part of the supplementary information (SI). Supplementary information: synthesis and characterization, additional ^1^H NMR, ROESY, NOESY, ^13^C NMR, ^77^Se NMR spectra, HR-MS and computational details. See DOI: https://doi.org/10.1039/d5sc10108e. CCDC 2503281 (1) and 2503282 (2) contain the supplementary crystallographic data for this paper.^57*a*,*b*^
