# Supplementary material for: Chalcogen bonding-directed photoresponsive helix from azo-fused H-bonded arylamide foldamers
Source: Chem Sci. 2026 May 5;17(24):11941–53. doi: 10.1039/d5sc10108e (PMC13158860; doi:10.1039/d5sc10108e)
Supplement: SC-017-D5SC10108E-s001 [file SC-017-D5SC10108E-s001.pdf]

Supplementary Information (SI) for Chemical Science.
This journal is © The Royal Society of Chemistry 2026

Supporting Information

Chalcogen Bonding Directed Photoresponsive Helix from Azo-Fused H-Bonded Arylamide Foldamers

Contents

1. General experimental 2
2. Procedure for the synthesis of compound **1**, **2**, **3** 2-7
3. ^{1}H NMR and ^{13}C NMR spectra of compound **1**, **2**, **3** 8-19
4. ^{77}Se spectra of compound **3** 20
5. HR-MS (ESI) of all new compounds 20-25
6. 2D ROESY spectrum of compound **1** 26
7. ^{1}H NMR for the isomerization of compound **2** 27
8. 2D ROESY for the isomerization of compound **2** in CD_2Cl_2 28
9. The reversible cyclic isomerization of compound **2**. 29-32
10. Gibbs free energy and Enthalpy pathway of compound **2** 33
11. The UV-vis absorption spectra of compound **3** 33
12. 2D ROESY for the isomerization of compound **3** 34-35
13. PXRD data of compound **3** 36-37
14. Quantum yield of compound **2** and **3** 38-39
15. Isomerization kinetic rate constants of compound **2** and **3** 40-42
16. Computational details 43-90
17. CIF report of compound **1** and **3** 91-97

General methods. All reagents were obtained from commercial suppliers and used without further purification unless otherwise noted. ^{1}H, ^{13}C and ^{77}Se NMR spectra were recorded with a 400 MHz or 100 MHz or 76.3 MHz spectrometer in the indicated solvents at 25 °C. Chemical shifts are expressed in parts per million (δ) using residual proton resonances of the deuterated solvents as the internal standards. The external standard method was used to calibrate the chemical displacement in ^{77}Se NMR spectra, and the external standard was pure liquid Me_2Se without deuterium reagent. Crystals were measured using Bruker D8 Venture-Metaljet diffractometer equipped with an PHOTON II area detector and HELIOS multilayer optics monochrommated Cu-K and Ga-K alpha radiation (lambda = 1.54184 and 1.34139 Å). Crystal structures were solved by direct method and refined by full-matrix least-squares methods based on F2 using SHELXL-2018 software. Crystals of **1** and **3** were grown by evaporating their solution in CH_2Cl_2 and DCM. All the crystal structure diagrams in the manuscript and SI were drawn by mercury software. CCDC (Nos. 2503281, 2503282) contains the related crystallographic data, which can be obtained free of charge from The Cambridge Crystallographic Data Centre via www.ccdc.cam.ac.uk/data_request/cif.

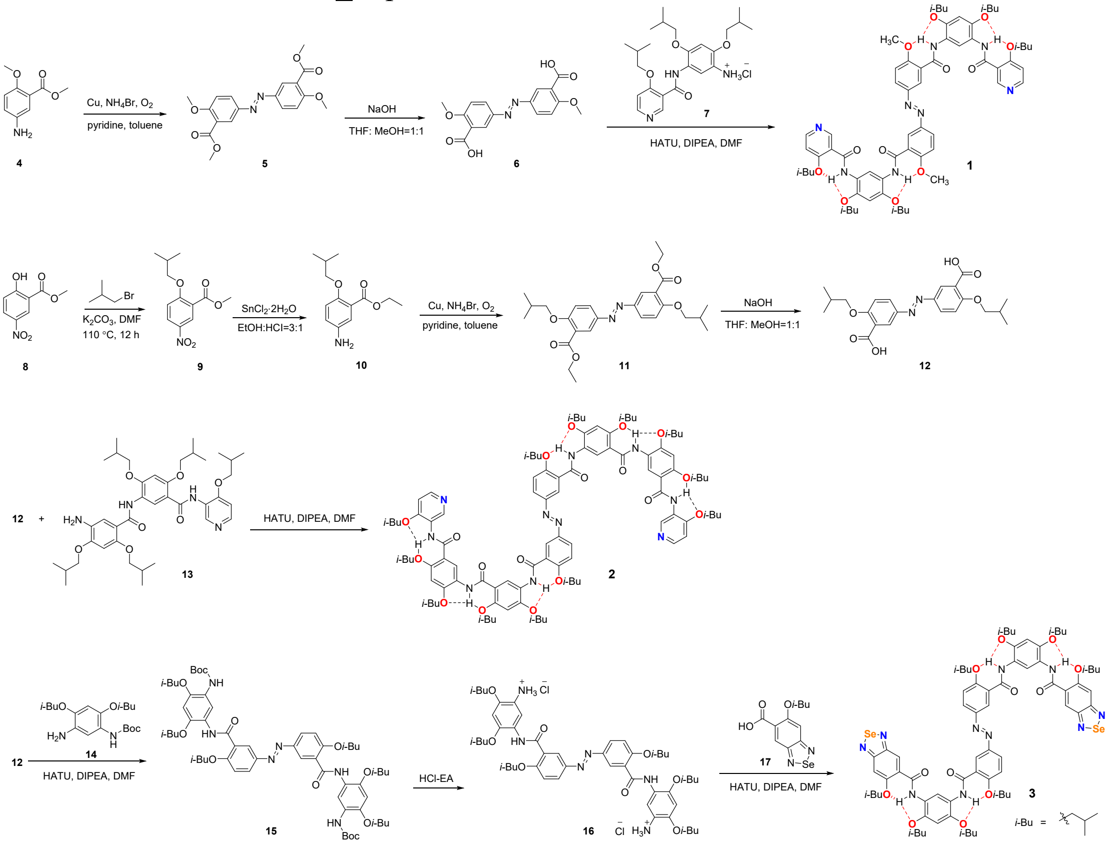

Compound 5. To a mixture of compound **4** (780 mg, 4.3 mol) in toluene (10 mL) was added Cu powder (546 mg, 8.6 mmol), NH_4Br (842 mg, 8.6 mmol), and Pyridine (1.0 mL). The mixture was heated to 110 °C under O_2 atmosphere and stirred at this temperature for 12 hs. Then the reaction was quenched with water (10 ml). The mixture was extracted with DCM (30*3). The combined organic phase was wash with fresh water for three times and dired with anhydrous sodium sulfate. The dried solution was filtered and concentrated to obtain the crude product. The crude product was further purified by column chromatography (petroleum ether and ethyl acetate, 5:1 to 3:1) to get the pure product (450 mg, 58%). ^{1}HNMR (400 MHz, $CDCl_3$): δ 8.40 (d, J = 2.8 Hz, 2H), 8.07 (dd, J = 8.8, 2.4 Hz, 2H), 7.11 (d, J = 8.8 Hz, 2H), 4.01 (s,

6H), 3.94 (s, 6H). ^{13}C NMR (100 MHz, $CDCl_3$): δ 166.03, 160.98, 145.77, 128.05, 126.36, 120.45, 112.20, 56.44, 52.26. HR-MS (ESI): Calcd for $C_{18}H_{18}N_2NaO_6$ $[M+Na]^+$: 381.1063. Found: 381.1064.

Compound 6. To a solution of compound **5** (430 mg, 1.2 mmol) in THF/MeOH (*V/V*:1/1, 30 mL) was added the MeOH solution of sodium hydroxide (0.17 g, 4.2 mmol). The mixture was heated to 70 °C and stirred for 2 hours. After the reaction was completed, the reaction solution was concentrated and redissolved in water (20 mL). The moxture was acidified with 1M hydrochloric acid solution to pH = 2, and a large amount of solid precipitates. It was then filtered, and the obtained solid was washed with water to obtain a yellow solid product (375 mg, 95%). ^{1}HNMR (400 MHz, DMSO-d_6): δ 12.98 (s, 2H), 8.16 (d, J = 2.8 Hz, 2H), 8.08 (dd, J = 8.8 Hz, 2.8 Hz, 2H), 7.34 (d, J = 8.8 Hz, 2H), 3.94 (s, 6H). ^{13}C NMR (100 MHz, DMSO-d_6): δ 167.12, 160.70, 145.42, 128.56, 124.45, 122.37, 113.59, 56.78. HR-MS (ESI): Calcd for $C_{16}H_{15}N_2O_6$ $[M+H]^+$: 331.0930. Found: 331.0932.

Compound 1. To a solution of compounds **6** (230 mg, 0.7 mmol) in DMF (30 mL) were added HATU (800 mg, 2.1 mmol) and DIPEA (270 mg, 2.1 mmol). The mixture was stirred at room temperature for 20 mins. Then compound **7** (652 mg, 1.4 mmol) was added. The mixture was warmed to 40 ^{o}C and stirred for 12 hours. Then ice-water (100 mL) was added to quench to reaction. The aqueous was extracted with DCM (3*50 mL). The combined organic phase was washed with saturated sodium chloride solution (100 mL) and water (100 mL) in sequence. Then the organic phase was dried with anhydrous sodium sulfate and concentrated to get crude product. The crude product was further purified by column chromatography (DCM and MeOH, 50:1 to 20:1) to get the pure product (330 mg, 41%). ^{1}HNMR (400 MHz, $CDCl_3$): δ 9.99 (s, 2H), 9.41-9.32 (m, 6H), 8.94 (d, J = 2.6 Hz, 2H), 8.57 (d, J = 5.9 Hz, 2H), 8.08 (dd, J = 8.8, 2.6 Hz, 2H), 7.16 (d, J = 9.0 Hz, 2H), 6.91 (d, J = 5.9 Hz, 2H), 6.55 (s, 2H), 4.12 (s, 6H), 4.03 (d, J = 6.9 Hz, 4H), 3.81 (dd, J = 9.7Hz, 6.8 Hz, 8H), 2.30-2.23 (m, 2H), 2.23-2.16 (m, 2H), 2.09 (m, J = 13.4, 6.7 Hz, 2H), 1.08 (dd, J = 14.6, 6.7 Hz, 24H), 1.00 (d, J = 6.7 Hz, 12H). ^{13}C NMR (100 MHz, $CDCl_3$): δ 162.60, 162.09, 161.65, 159.56, 158.87, 154.33, 153.30, 147.11, 146.47, 146.16, 128.15, 126.70, 123.09, 121.33, 120.10, 118.54, 117.29, 111.99, 107.46, 97.85, 75.98, 75.74, 75.51, 28.49, 28.32, 27.86, 19.34, 19.26, 19.21. HR-MS (ESI): Calcd for $C_{64}H_{80}N_8NaO_{12}$ $[M+Na]^+$: 1175.5793. Found: 1175.5753.

Compound 9. To a solution of compounds **8** (3.4 g, 17.3 mmol) in DMF (80 mL) was added K_2CO_3 (7.1 g, 34.5 mmol). The mixture was heated to 80 ^{o}C and stirred for 1 hour at this temperature. Then bromoisobutane (4.7 g, 34.5 mmol) was added by dropwise. The solution was heated to 110 ^{o}C and stirred for 12 hours at this temperature. Then water (100 mL) was added to quench to reaction. The aqueous was extracted with EA (3*100 mL). The combined organic phase was washed with saturated sodium chloride solution (300 mL) and water (300 mL) in sequence. Then the organic phase was dried with anhydrous sodium sulfate and concentrated to get crude product. The crude product was further purified by column chromatography (PE: EA, 5:1) to get the pure product (3.3 g, 76%). ^{1}HNMR (400 MHz, $CDCl_3$): δ 8.71 (d, J = 3.2 Hz, 1H), 8.33 (dd, J = 9.2 Hz, 2.8 Hz, 1H), 7.03 (d, J = 9.2 Hz, 1H), 3.94-3.91 (m, 5H), 2.23-2.16 (m, 1H), 1.09 (d, J = 6.8 Hz, 6H). ^{13}C NMR (100 MHz, $CDCl_3$): δ 164.71, 163.30, 140.37, 128.82, 127.90, 120.50, 112.64, 75.90, 52.43, 28.27, 19.03. HR-MS (ESI): Calcd for $C_{12}H_{16}NO_5$ $[M+H]^+$: 254.1028. Found: 254.1031.

Compound 10. To a solution of compounds **9** (1.53 g, 60.8 mmol) in EtOH/37% wt con. HCl (*V/V* 3:1，40 mL) were added $SnCl_2$ (4.2 g, 18.5 mmol). The mixture was heated to 90 ^{o}C and stirred for 4 hours at this temperature. Then the mixture was

poured into ice-water (200 mL). The pH was adjusted to 14 with 2 M NaOH in an ice bath. The aqueous was extracted with EA (3*100 mL). The combined organic phase was dried with anhydrous sodium sulfate and concentrated to get crude product. The crude product was further purified by column chromatography (PE: EA, 5:1). A yellow oily product was obtained (0.8 g, 56%). ^{1}HNMR (400 MHz, $CDCl_3$): δ 7.11 (s, 1H), 7.61 (s, 2H), 4.33 (q, J = 7.2 Hz, 2H), 3.69 (d, J = 6.4 Hz, 2H), 3.34 (s, 2H), 2.10-2.03 (m, 1H), 1.35 (t, J = 7.2 Hz, 3H), 1.01 (d, J = 6.8 Hz, 6H). ^{13}C NMR (100 MHz, $CDCl_3$): δ 166.85, 151.75, 139.53, 121.51, 120.10, 117.90, 115.38, 76.27, 60.81, 28.50, 19.26, 14.35. HR-MS (ESI): Calcd for $C_{13}H_{20}NO_3$ $[M+H]^+$: 238.1443. Found: 238.1448.

Compound 11. To a mixture of compound **10** (0.46 g, 2.0 mmol) in toluene (10 mL) was added Cu powder (0.19 g, 3.0 mmol), NH_4Br (0.26 g, 2.70 mmol), and Pyridine (1.0 mL). The mixture was heated to 110 ^{o}C under O_2 atmosphere and stirred at this temperature for 12 hs. Then the reaction was quenched with water (10 ml). The mixture was extracted with DCM (30*3). The combined organic phase was wash with fresh water for three times and dired with anhydrous sodium sulfate. The dried solution was filtered and concentrated to obtain the crude product. The crude product was further purified by column chromatography (petroleum ether and ethyl acetate, 5:1 to 3:1) to get the pure product (250 mg, 54%). ^{1}HNMR (400 MHz, $CDCl_3$): δ 8.38 (d, J = 2.4 Hz, 2H), 8.01 (dd, J = 8.8 Hz, 2.4 Hz, 2H), 7.05 (d, J = 8.8 Hz, 2H), 4.4 (q, J = 7.2 Hz, 4H), 3.89 (d, J = 6.4 Hz, 4H), 2.22-2.26 (m, 2H), 1.42 (t, J = 7.2 Hz, 6H), 1.09 (d, J = 6.8 Hz, 12H). ^{13}C NMR (100 MHz, $CDCl_3$): δ 166.08, 160.42, 145.64, 127.32, 126.66, 121.09, 112.93, 75.39, 61.10, 28.40, 19.21, 14.40. HR-MS (ESI): Calcd for $C_{26}H_{35}N_2O_6$ $[M+H]^+$: 471.2495. Found: 471.2501.

Compound 12. To a solution of compound **11** (0.61 g, 1.30mmol) in THF/MeOH (*V/V*:1/1, 40 mL) was added the MeOH solution of sodium hydroxide (0.17 g, 4.2 mmol). The mixture was heated to 70 °C and stirred for 2 hours. After the reaction was completed, the reaction solution was concentrated and redissolved in water (25 mL). The moxture was acidified with 1M hydrochloric acid solution to pH = 2, and a large amount of solid precipitates. It was then filtered, and the obtained solid was washed with water to obtain a yellow solid product (490 mg, 91%). ^{1}HNMR (400 MHz, DMSO-d_6): δ 12.98 (s, 2H), 8.16 (d, J = 2.8 Hz, 2H), 8.08 (dd, J = 8.8 Hz, 2.8 Hz, 2H), 7.34 (d, J = 8.8 Hz, 2H), 3.94 (s, 6H). ^{13}C NMR (100 MHz, DMSO-d_6): δ 167.28, 160.16, 145.29, 128.25, 124.55, 122.67, 114.21, 75.16, 28.26, 19.35. HR-MS (ESI): Calcd for $C_{22}H_{27}N_2O_6$ $[M+H]^+$: 415.1869. Found: 415.1871.

Compound 2. To a solution of compounds **6** (47.9 mg, 0.12 mmol) in DMF (20 mL) were added HATU (365 mg, 0.96 mmol) and DIPEA (167 mg, 0.96 mmol). The mixture was stirred at room temperature for 20 mins. Then compound **7** (200 mg, 0.29 mmol) was added. The mixture was warmed to 40 ^{o}C and stirred for 12 hours. The reaction was stirred at room temperature for 24 hours. Then ice-water (50 mL) was added to quench to reaction. The aqueous was extracted with DCM (3*50 mL). The combined organic phase was washed with saturated sodium chloride solution (100 mL) and water (100 mL) in sequence. Then the organic phase was dried with anhydrous sodium sulfate and concentrated to get crude product. The crude product was further purified by column chromatography (DCM and MeOH, 30:1) to get the pure product (130 mg, 61%). ^{1}HNMR (400 MHz, $CDCl_3$): δ 10.27 (s, 2H), 9.91 (s, 2H), 9.84 (s, 2H), 9.62 (s, 2H), 9.00 (s, 2H), 8.76 (s, 2H), 8.55 (d, J = 6.4 Hz, 2H), 8.45 (d, J = 2.4 Hz, 2H), 8.11 (dd, J = 8.8 Hz, 2.8 Hz, 2H), 7.69 (d, J = 6.8 Hz, 2H), 7.50 (d, J = 6.2 Hz, 2H), 7.00 (s, 2H), 6.97 (s, 2H), 4.26 (d, J = 6.8 Hz, 4H), 4.20-4.14 (m, 12H), 4.08-4.03 (m, 8H), 2.20-2.09 (m, 12H), 1.03-0.98 (m, 62H). ^{13}C NMR (100 MHz,

$CDCl_3$): δ 164.05, 163.23, 162.55, 158.93, 154.35, 152.43, 146.56, 126.80, 124.07, 123.05, 122.40, 122.29, 114.09, 97.96, 76.78, 76.66, 75.82, 75.64, 28.18, 28.00, 27.91, 27.82, 27.59, 18.99, 18.94, 18.89, 18.87, 18.50. HR-MS (ESI): Calcd for $C_{100}H_{135}N_{10}O_{18}$ $[M+H]^+$: 1764.9989. Found: 1764.9990.

Compound 15. To a solution of compounds **12** (549 mg, 1.3 mmol) in DMF (40 mL) were added HATU (2.52 g, 6.6 mmol) and DIPEA (1.71 g, 13.3 mmol). The mixture was stirred at room temperature for 20 mins. Then compound **14** (1.07 g, 13.3 mmol) was added. The mixture was stirred at rt for 48 hours. Then ice-water (100 mL) was added to quench to reaction. The aqueous was extracted with DCM (3*50 mL). The mixture was filtered to get crude product. The crude product was slurry in PE (15 mL) for 15 mins. Then the solid was filtered, and finally slurry in MeOH (15 mL) for 15 mins to get the pure product (1.25 g, 87%). ^{1}HNMR (400 MHz, $CDCl_3$): δ 9.71 (s, 2H), 8.97 (s, 2H), 8.86 (d, J = 2.4 Hz, 2H), 8.02 (dd, J = 8.4 Hz, 2.8 Hz, 2H), 7.13 (d, J = 8.8 Hz, 2H), 6.73 (s, 2H), 6.50 (s, 2H), 4.04 (d, J = 7.2 Hz, 4H), 3.77-3.74 (m, 8H), 2.28-2.05 (m, 6H), 1.54 (s, 18H), 1.07-0.99 (m, 36H). ^{13}C NMR (100 MHz, $CDCl_3$): δ 162.50, 158.43, 152.96, 146.93, 145.03, 144.73, 128.32, 126.07, 123.61, 121.31, 121.17, 114.59, 113.23, 98.46, 76.36, 76.26, 75.60, 28.43, 28.34, 28.02, 19.38, 19.30. HR-MS (ESI): Calcd for $C_{60}H_{86}N_6NaO_{12}$ $[M+Na]^+$: 1105.6201. Found: 1105.6194.

Compound 16. Compound **15** (1.0 g, 1.0 mmol) was added to hydrochloride gas in ethyl acetate (30 mL, 2.0 mM) and the suspension was stirred for 1 hour and then concentrated under reduce pressure to afford crude **16** (as hydrochloride salt). Then the crude product was slurry in solution of PE/EA (2:1, 20 mL) for 30 min. The mixture was filtered to get pure product as orange-yellow solid (810 mg, 92%). ^{1}HNMR (400 MHz, DMSO-d_6): δ 9.92 (s, 2H), 9.84 (s, 2H), 8.48 (d, J = 6.4 Hz, 2H), 8.40 (s, 2H), 8.12 (dd, J = 8.8 Hz, 2.8 Hz, 2H), 7.53 (d, J = 8.8 Hz, 2H), 6.97 (s, 2H), 4.18 (d, J = 6.8 Hz, 4H), 3.98 (d, J = 6.8 Hz, 4H), 3.92 (d, J = 6.4 Hz, 4H), 2.20-2.06 (m, 6H), 1.07-0.99 (m, 36H). ^{13}C NMR (100 MHz, DMSO-d_6) δ 162.69, 158.92, 149.91, 149.55, 146.21, 128.04, 125.45, 123.58, 120.29, 118.04, 115.11, 112.00, 99.45, 76.25, 75.67, 75.50, 28.29, 28.18, 27.91, 19.63, 19.44, 19.30. HR-MS (ESI): Calcd for $C_{50}H_{73}Cl_2N_6O_8$ $[M+H]^+$: 955.4867. Found: 955.4870.

Compound 3. To a solution of compounds **17** (159 mg, 0.53 mmol) in DMF (20 mL) were added HATU (271 mg, 0.71 mmol) and DIPEA (92 mg, 0.71 mmol). The mixture was protected with N_2 and stirred at room temperature for 20 mins. Then compound **16** (85 mg, 0.09 mmol) was added. The mixture was warmed to 40 ^{o}C and stirred for 12 hours. Then water (60 mL) was added to quench to reaction. The aqueous was extracted with DCM (3*50 mL). The combined organic phase was washed with saturated sodium chloride solution (100 mL) and water (100 mL) in sequence. Then the organic phase was dried with anhydrous sodium sulfate and concentrated to get crude product. The crude product was further purified by column chromatography (DCM and MeOH, 50:1) to get the pure product (85 mg, 66%). ^{1}HNMR (400 MHz, $CDCl_3$): δ 9.73 (s, 2H), 9.39 (s, 2H), 9.23 (s, 2H), 8.88 (d, J = 6.4 Hz, 2H), 8.68 (s, 2H), 8.05 (dd, J = 8.8 Hz, 2.8 Hz, 2H), 7.19 (s, 2H), 7.15 (d, J = 8.8 Hz, 2H), 6.58 (s, 2H), 4.08-4.02 (m, 8H), 3.82-3.80 (m, 8H), 2.36-2.24 (m, 4H), 2.15-2.06 (m, 4H), 1.09-1.00 (m, 48H). ^{13}C NMR (100 MHz, $CDCl_3$): δ 162.72, 161.84, 158.49, 157.68, 147.13, 146.95, 146.87, 130.42, 128.50, 127.34, 126.08, 123.45, 120.99, 120.30, 118.14, 113.26, 100.85, 98.56, 77.22, 76.39, 76.21, 75.97, 75.91, 28.34, 28.32, 28.04, 27.77, 19.38, 19.33, 19.30, 19.27. HR-MS (ESI): Calcd for $C_{72}H_{90}N_{10}NaO_{12}Se_2$ $[M+H]^+$: 1469.4968. Found: 1469.4943.

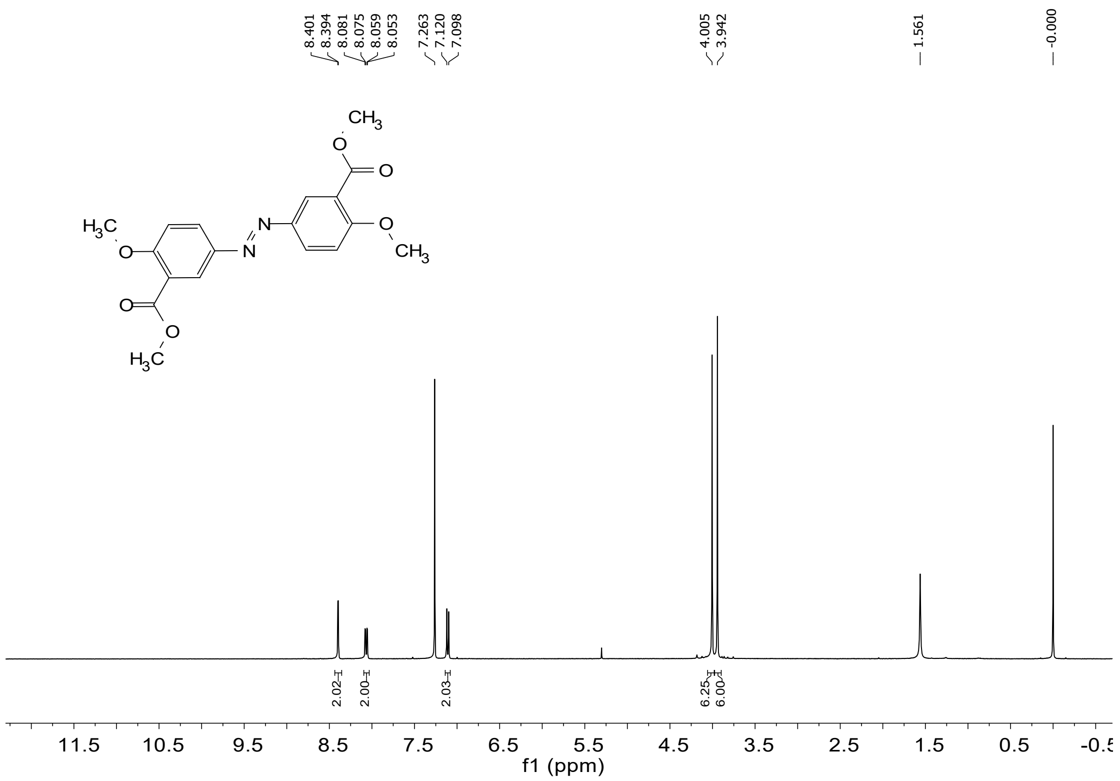

Figure S1. ^{1}H NMR (400 MHz, $CDCl_3$) spectrum of compound **5** at 25 °C.

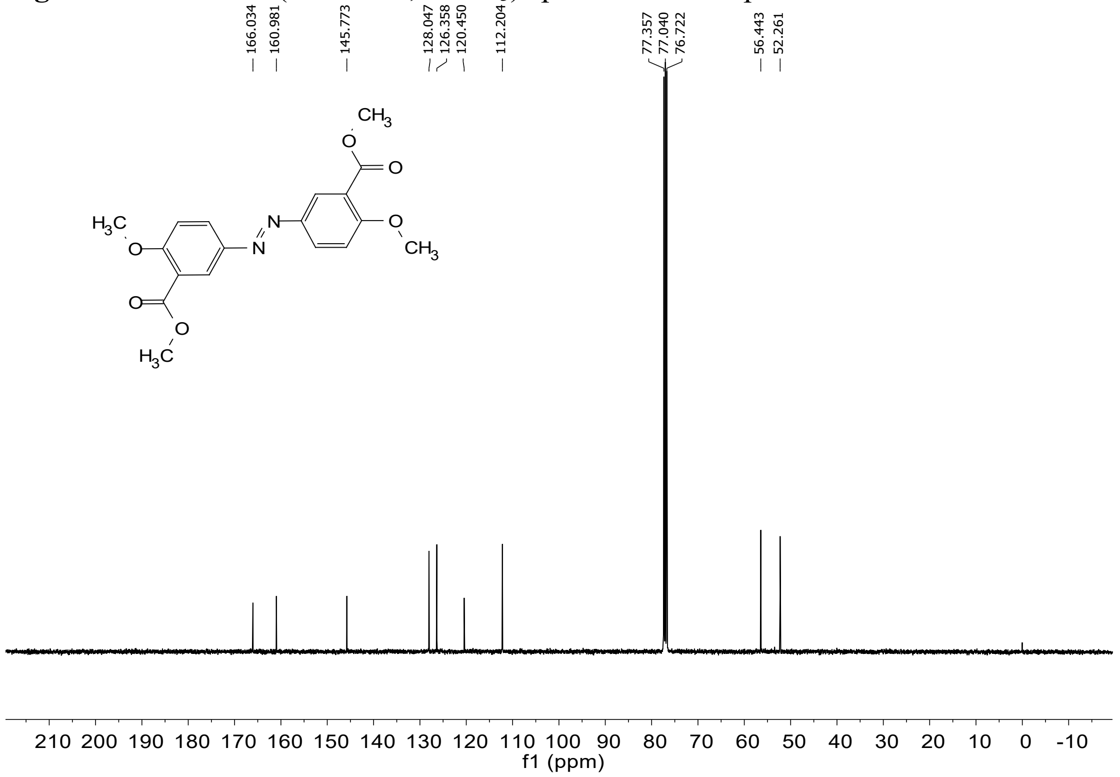

Figure S2. ^{13}C NMR (100 MHz, $CDCl_3$) spectrum of compound **5** at 25 °C.

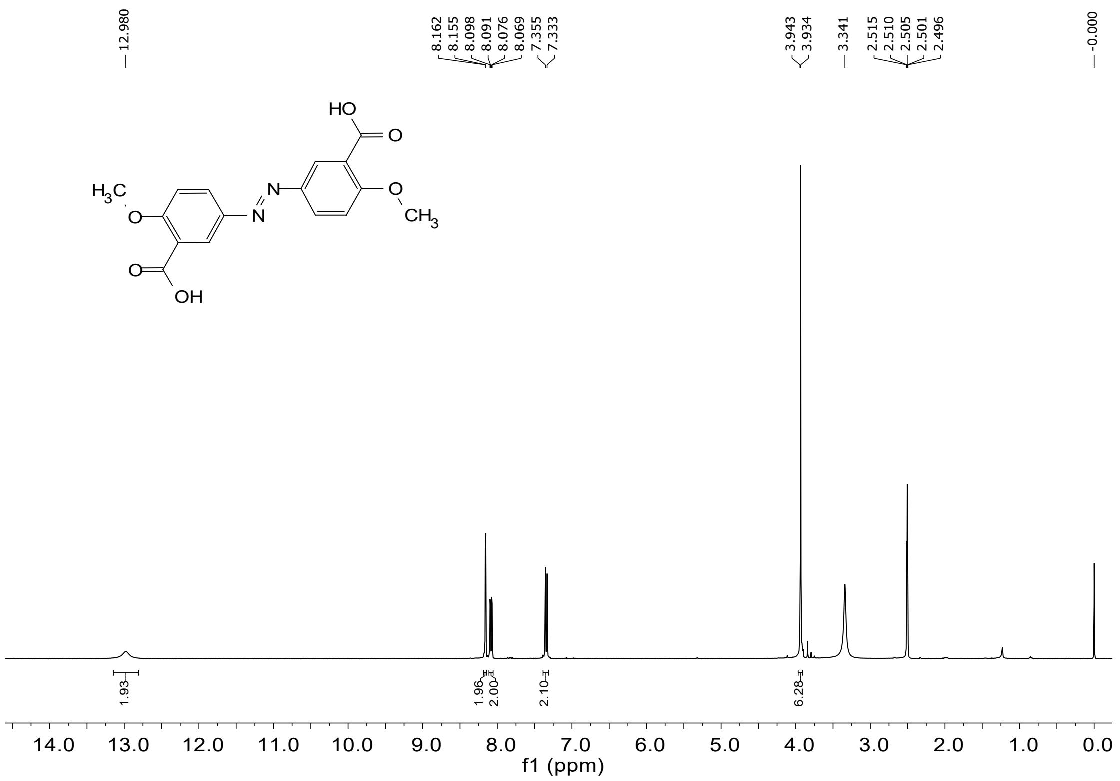

Figure S3. ^{1}H NMR (400 MHz, DMSO-d_6) spectrum of compound **6** at 25 °C.

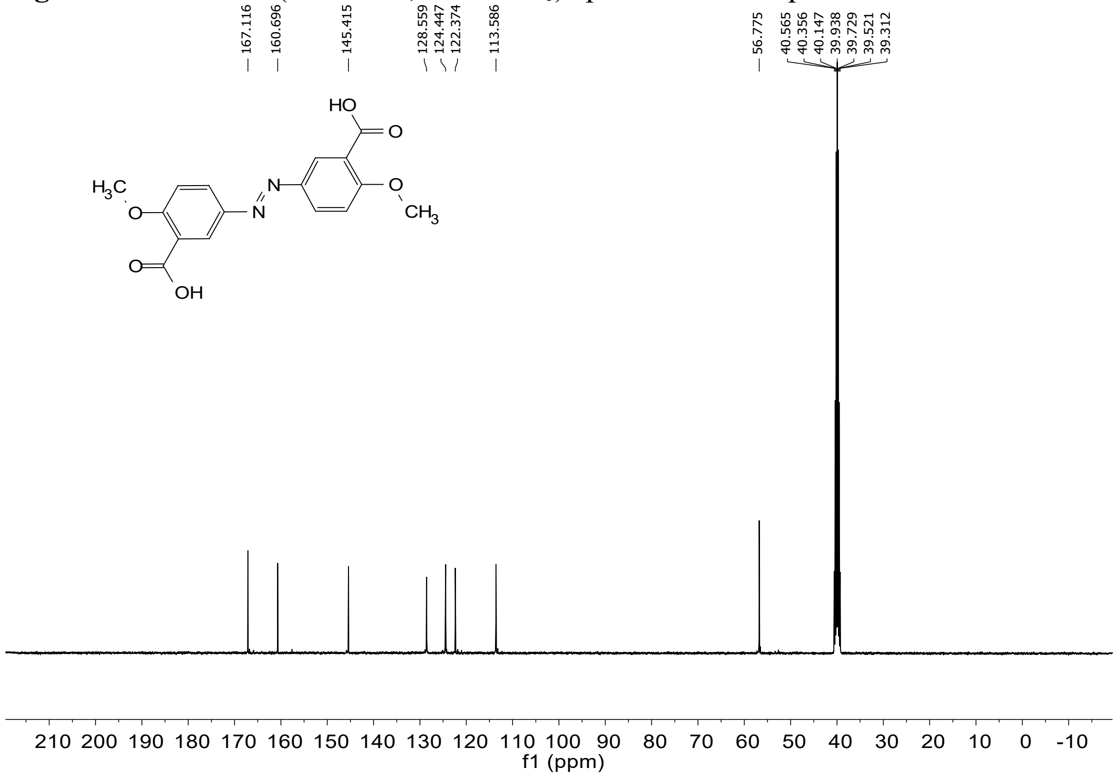

Figure S4. ^{13}C NMR (100 MHz, DMSO-d_6) spectrum of compound **6** at 25 °C.

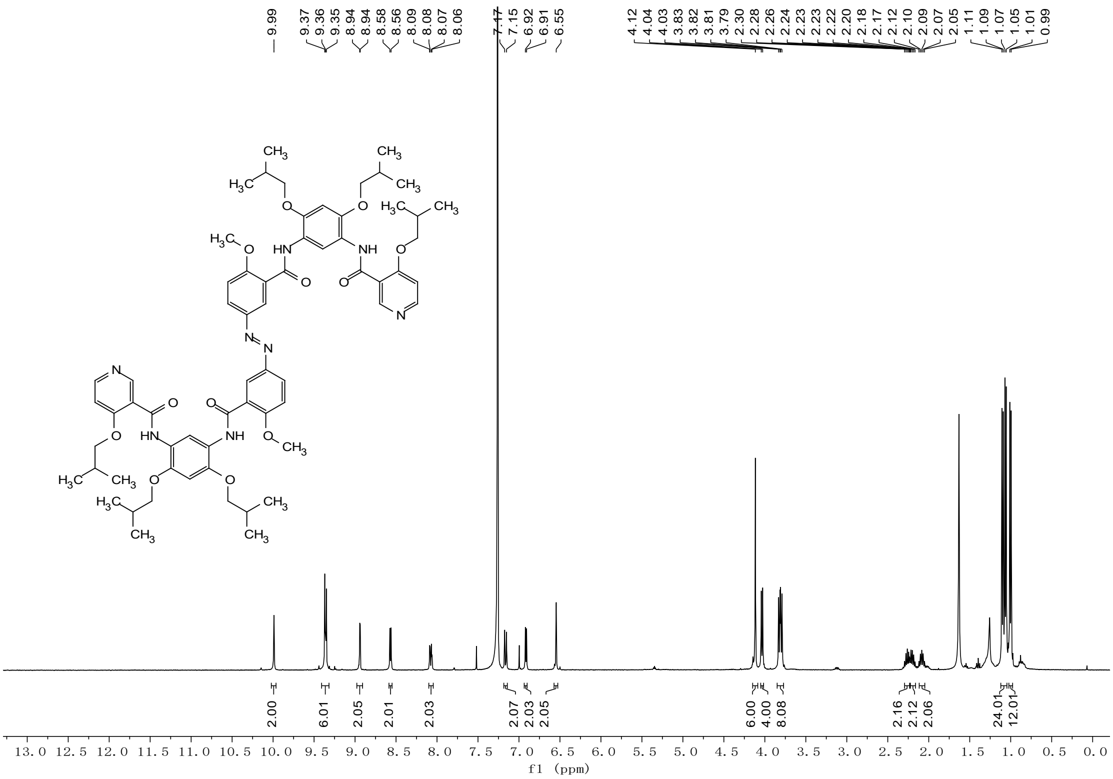

Figure S5. ^{1}H NMR (400 MHz, $CDCl_3$) spectrum of compound **1** at 25 °C.

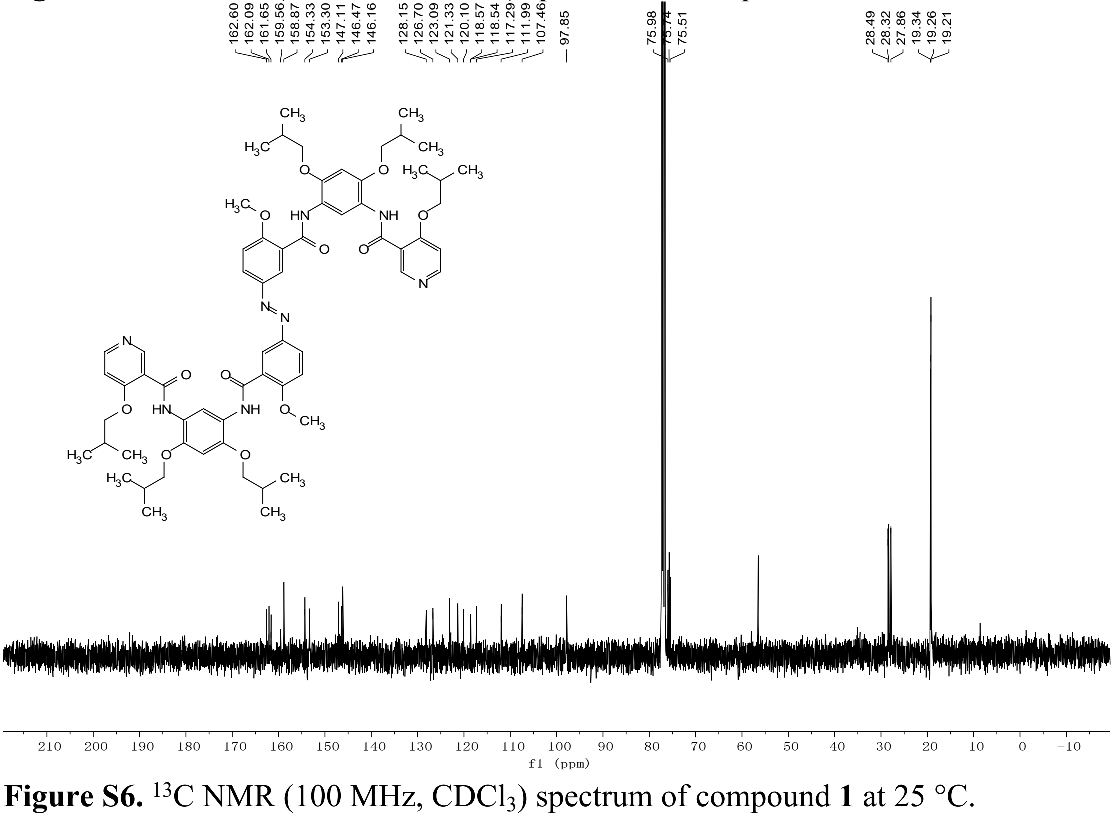

Figure S6. ^{13}C NMR (100 MHz, $CDCl_3$) spectrum of compound **1** at 25 °C.

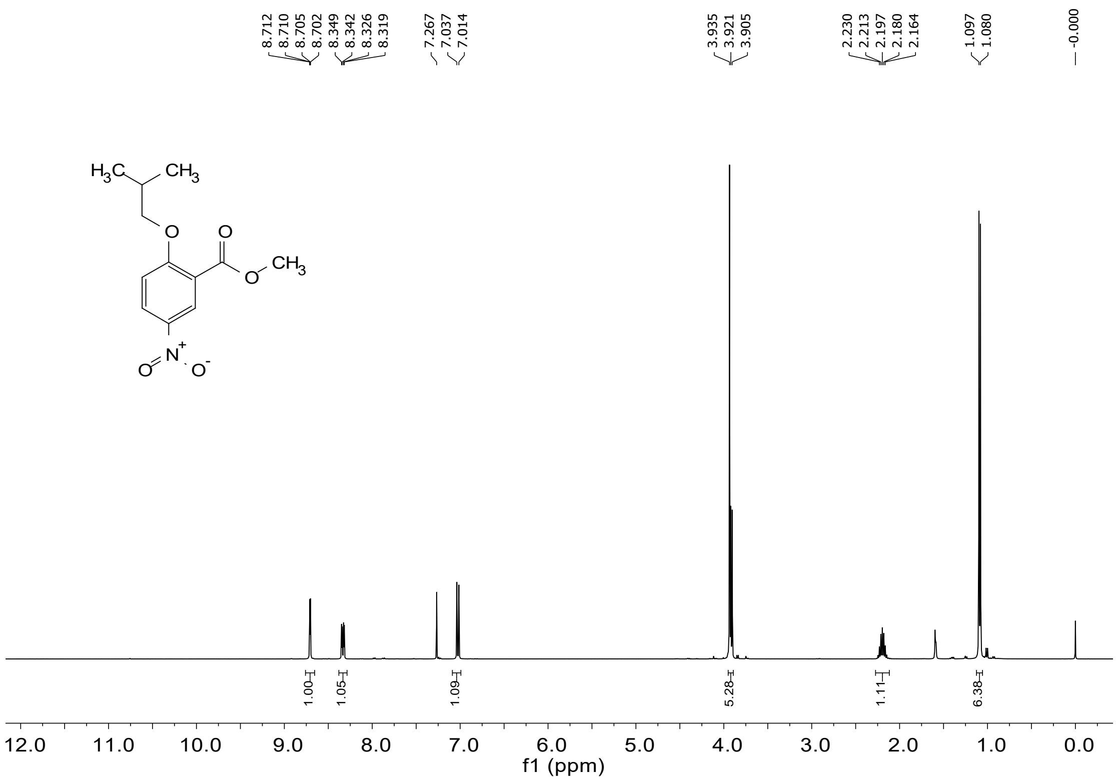

Figure S7. ^{1}H NMR (400 MHz, $CDCl_3$) spectrum of compound **9** at 25 °C.

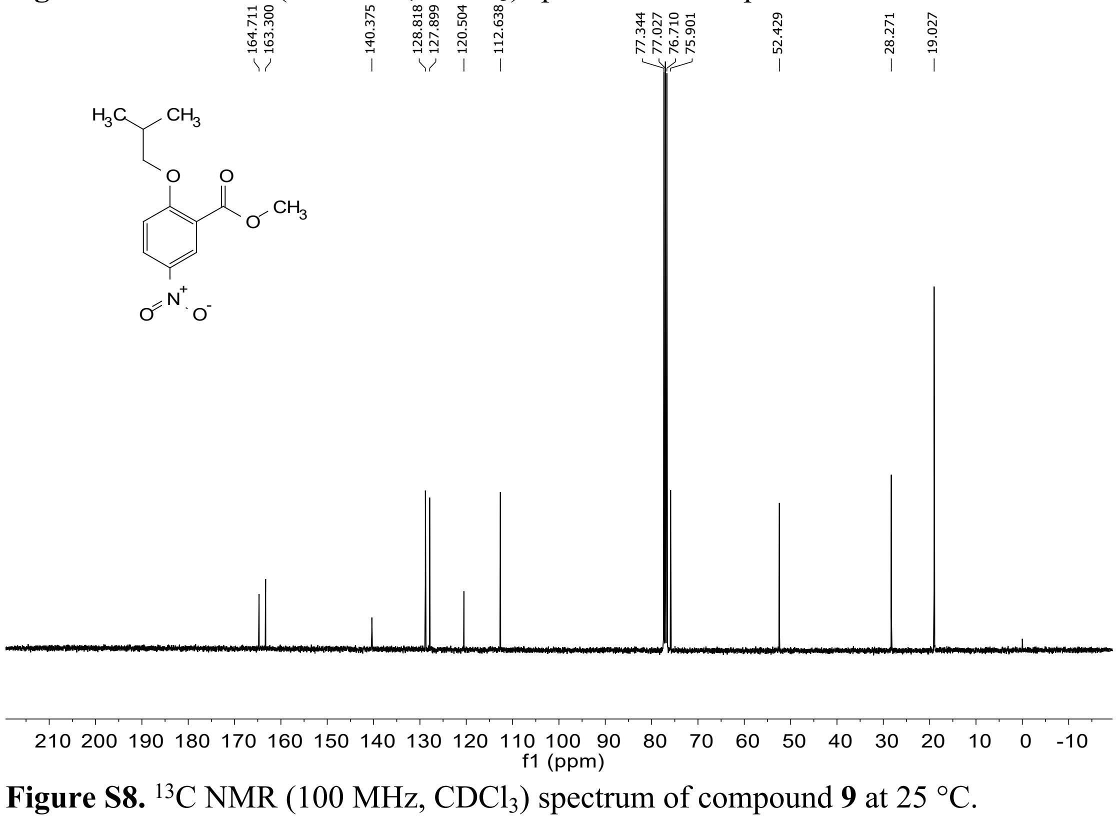

Figure S8. ^{13}C NMR (100 MHz, $CDCl_3$) spectrum of compound **9** at 25 °C.

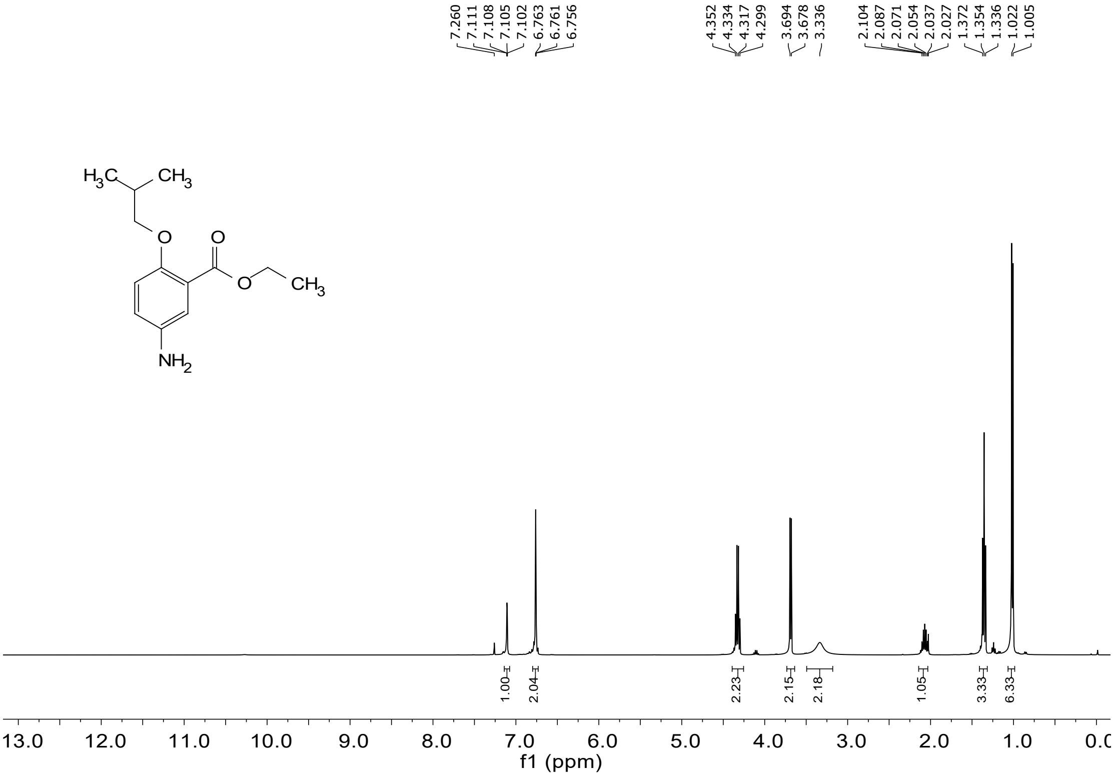

Figure S9. ^{1}H NMR (400 MHz, $CDCl_3$) spectrum of compound **10** at 25 °C.

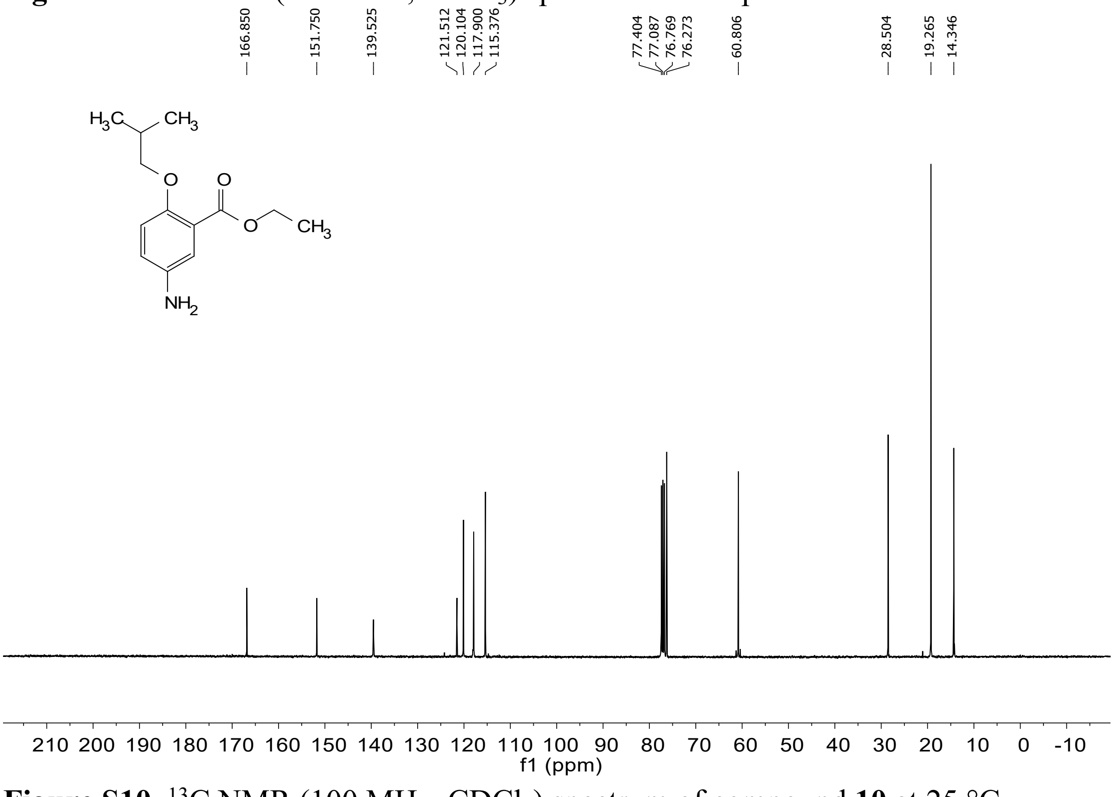

Figure S10. ^{13}C NMR (100 MHz, $CDCl_3$) spectrum of compound **10** at 25 °C.

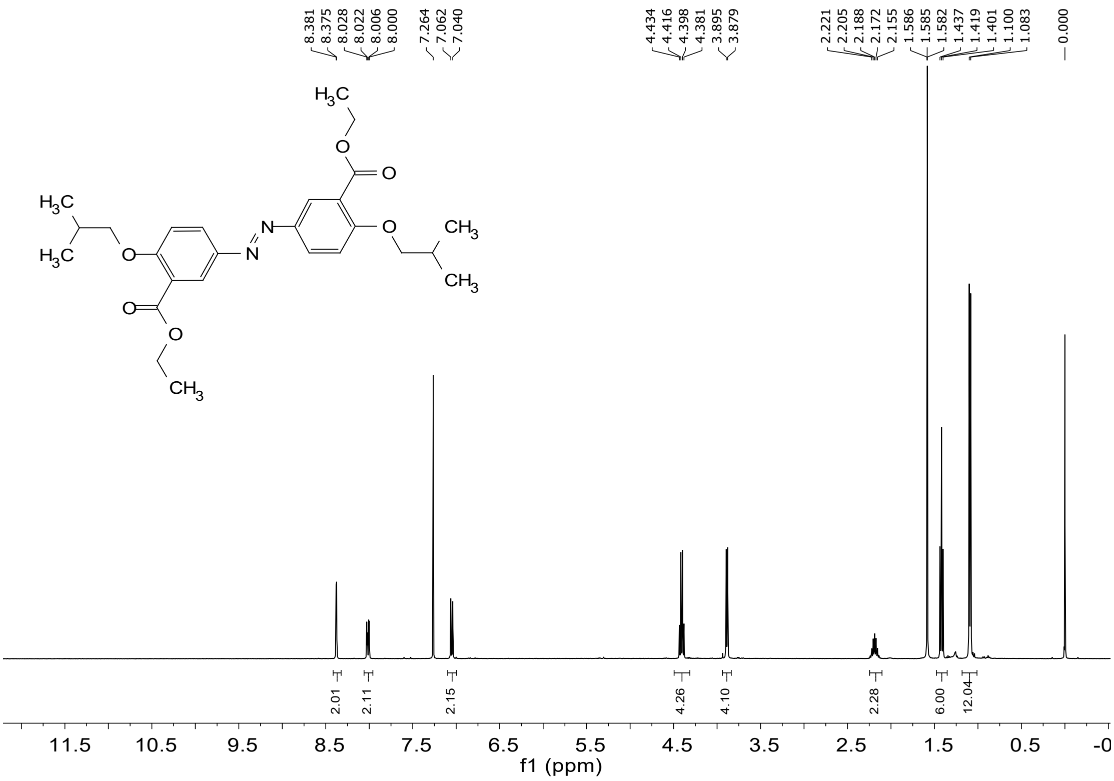

Figure S11. ^{1}H NMR (400 MHz, $CDCl_3$) spectrum of compound **11** at 25 °C.

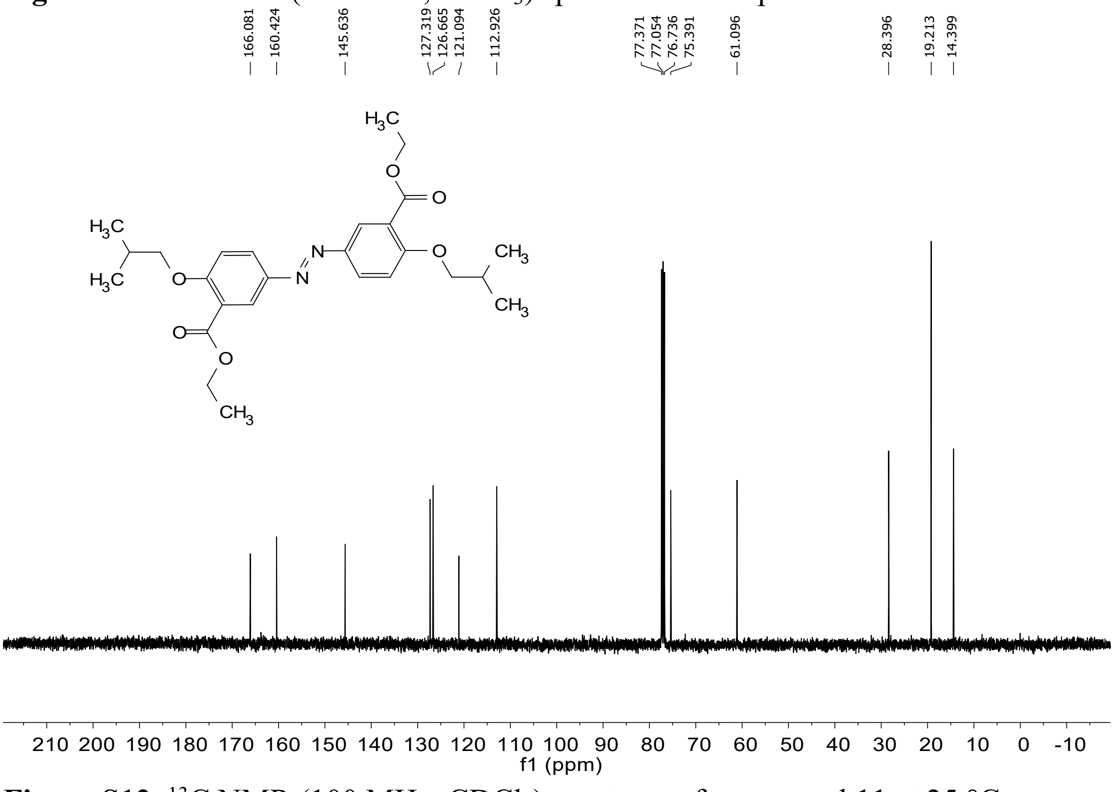

Figure S12. ^{13}C NMR (100 MHz, $CDCl_3$) spectrum of compound **11** at 25 °C.

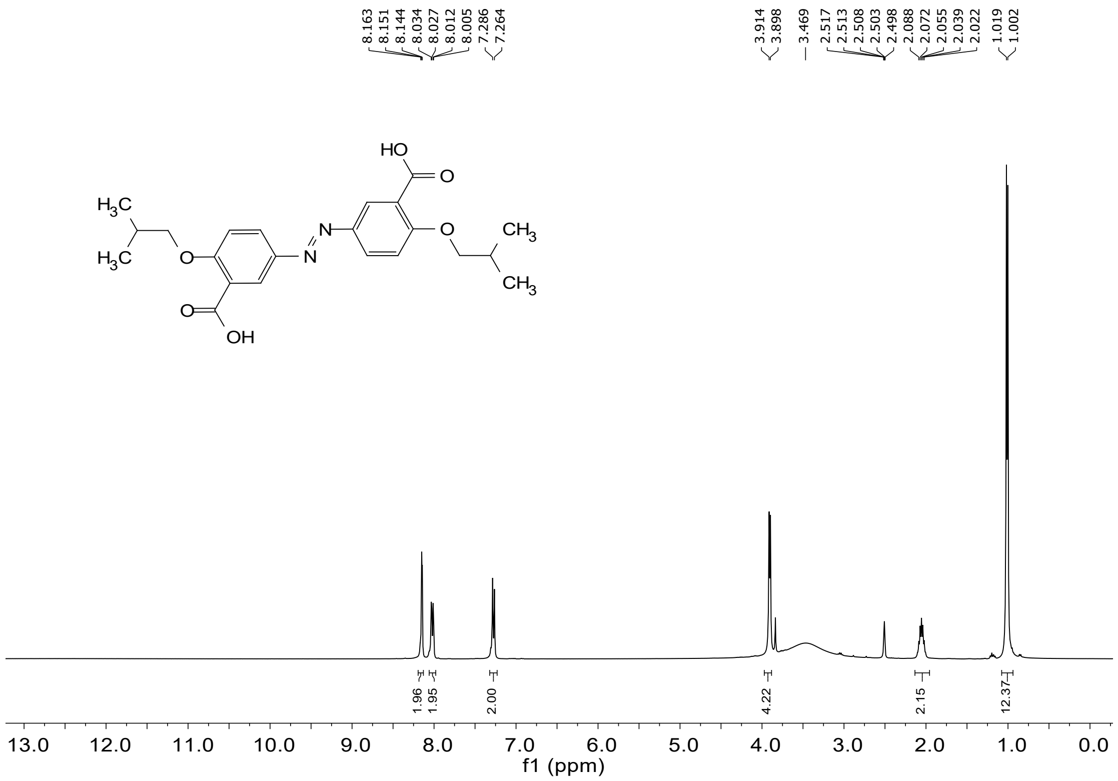

Figure S13. ^{1}H NMR (400 MHz, DMSO-d_6) spectrum of compound **12** at 25 °C.

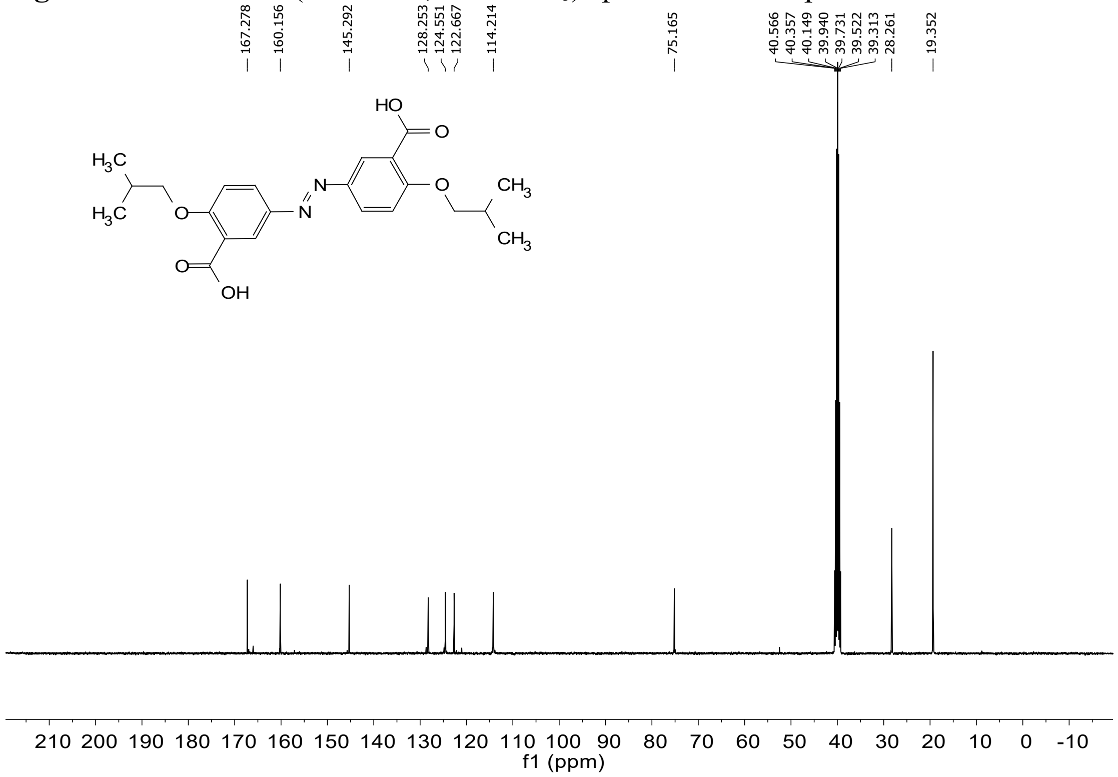

Figure S14. ^{13}C NMR (100 MHz, DMSO-d_6) spectrum of compound **12** at 25 °C.

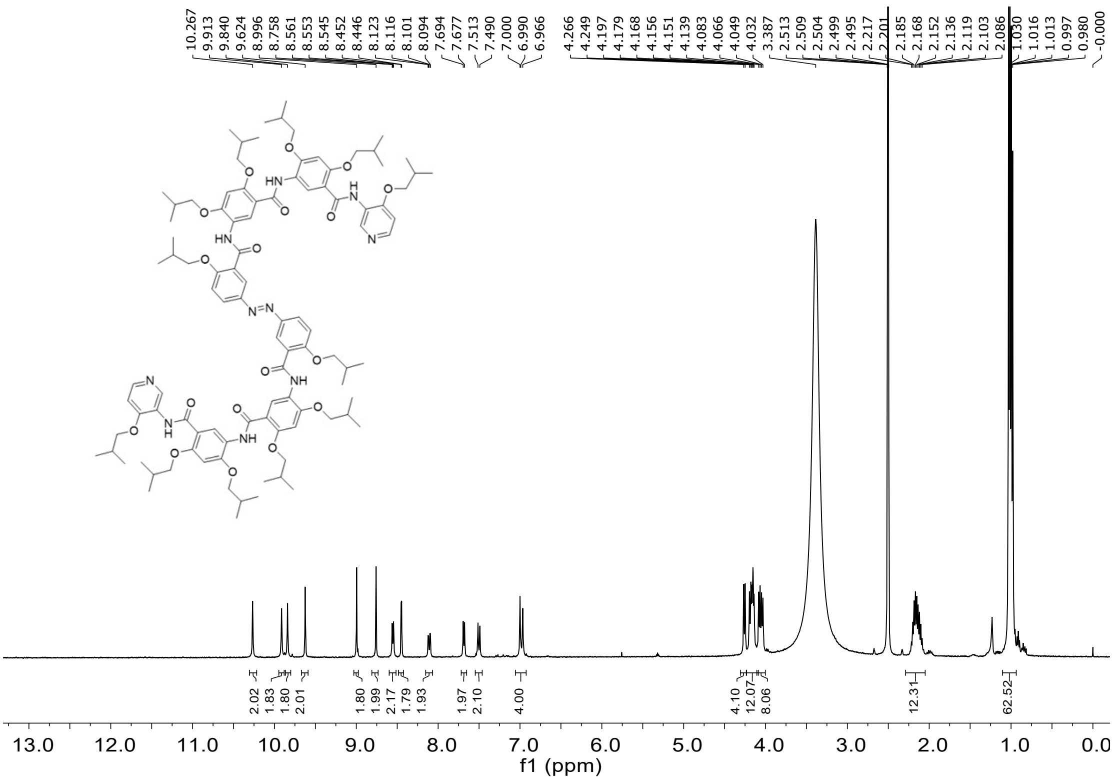

Figure S15. ^{1}H NMR (400 MHz, DMSO-d_6) spectrum of compound **2** at 25 °C.

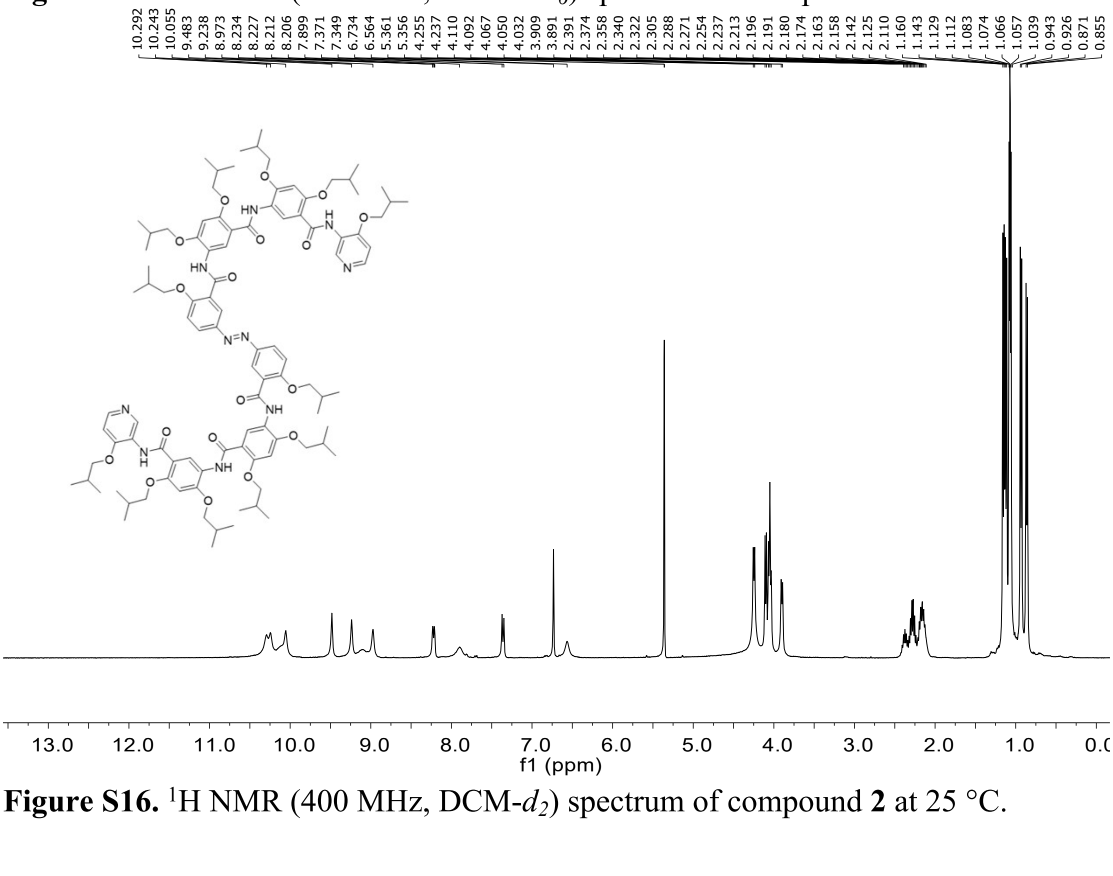

Figure S16. ^{1}H NMR (400 MHz, DCM-d_2) spectrum of compound **2** at 25 °C.

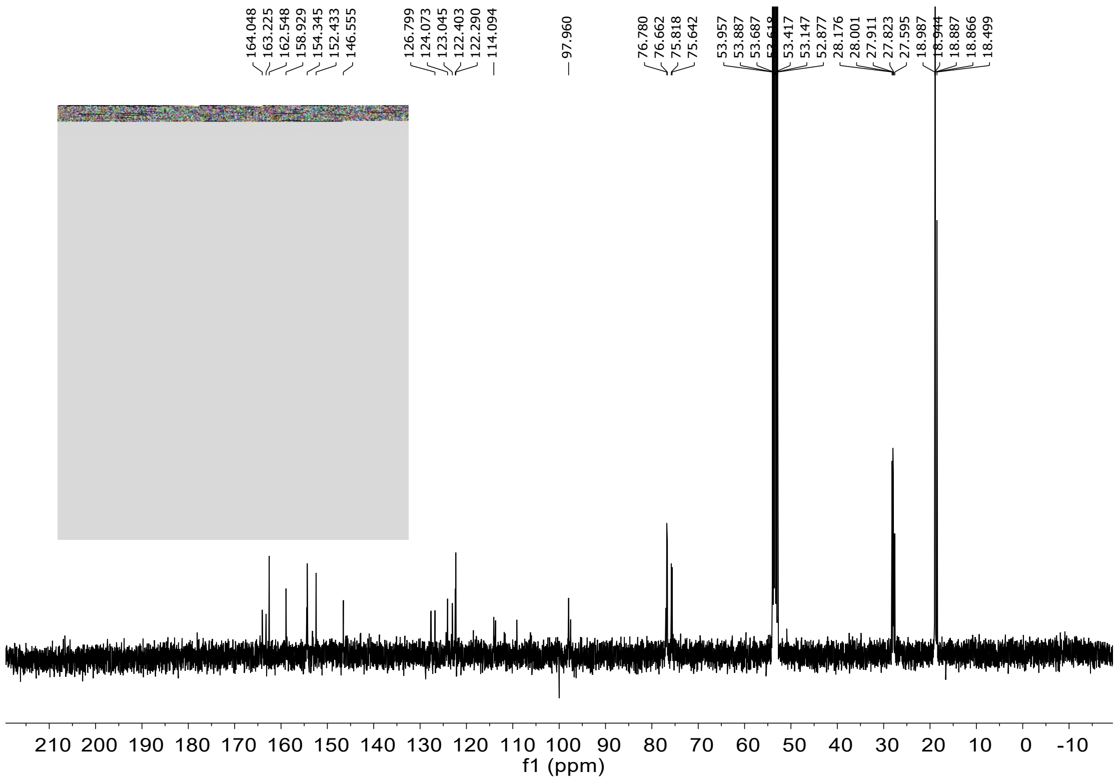

Figure S17. ^{13}C NMR (400 MHz, DCM-d_2) spectrum of compound **2** at 25 °C.

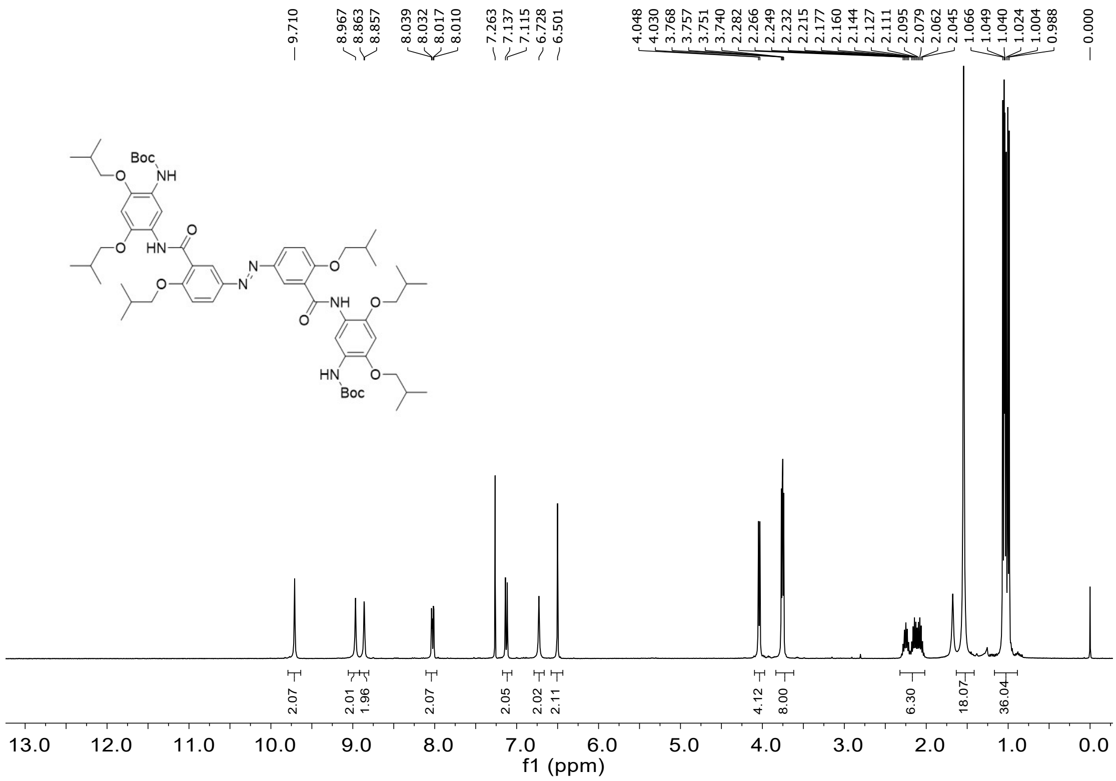

Figure S18. ^{1}H NMR (400 MHz, $CDCl_3$) spectrum of compound **15** at 25 °C.

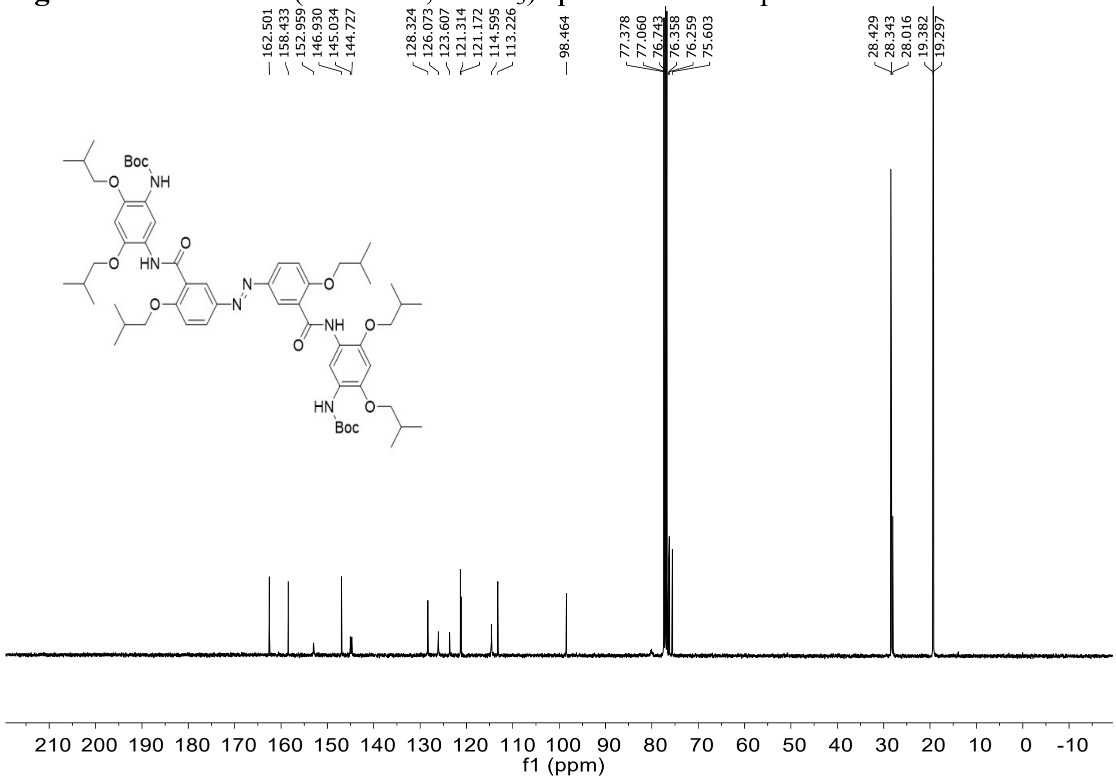

Figure S19. ^{13}C NMR (100 MHz, $CDCl_3$) spectrum of compound **15** at 25 °C.

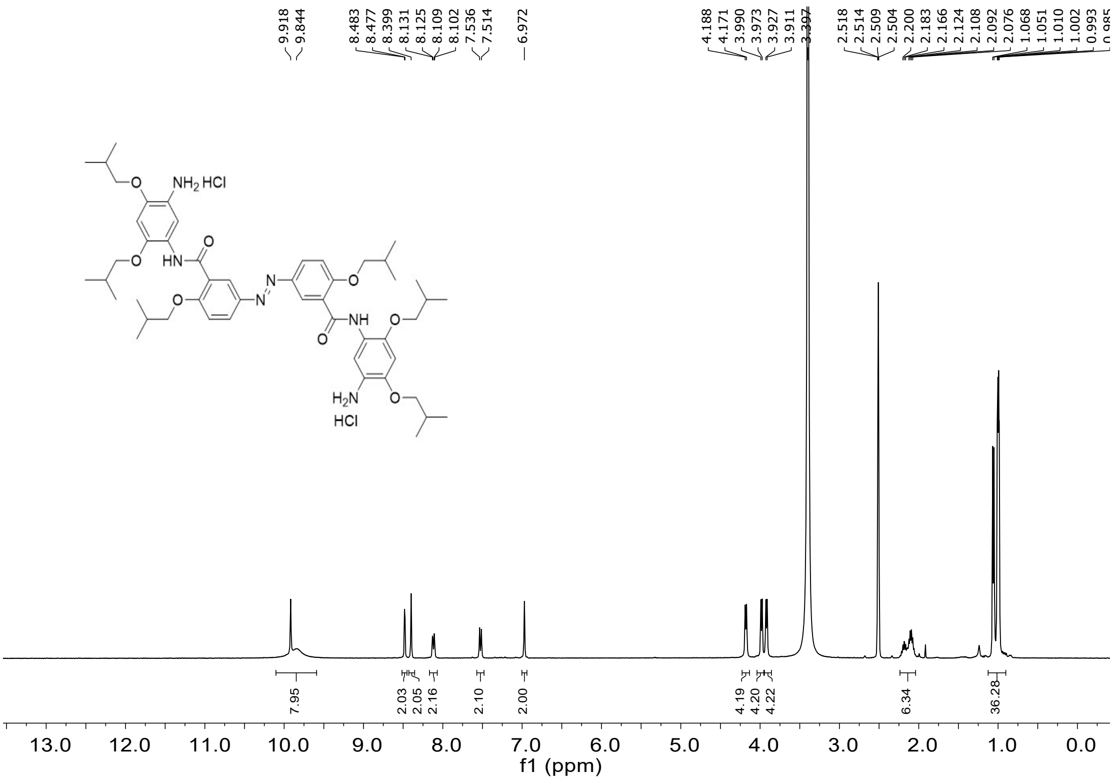

Figure S20. ^{1}H NMR (400 MHz, DMSO-d_6) spectrum of compound **16** at 25 °C.

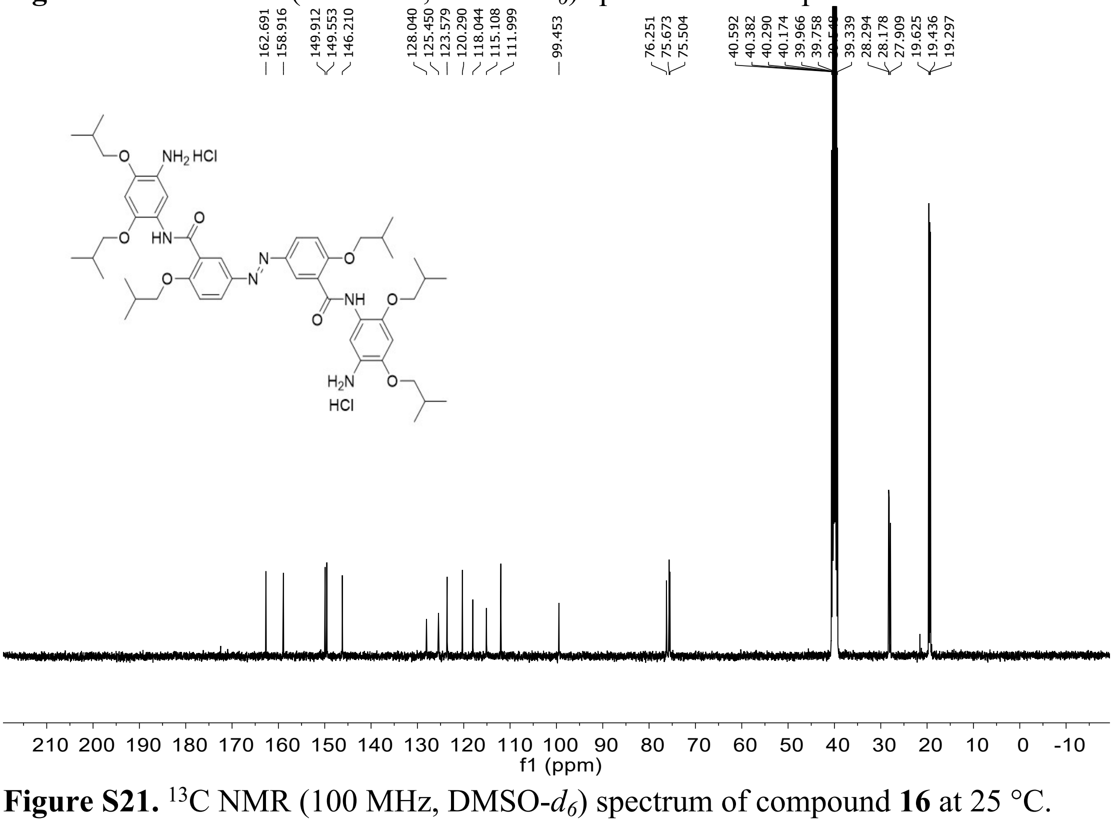

Figure S21. ^{13}C NMR (100 MHz, DMSO-d_6) spectrum of compound **16** at 25 °C.

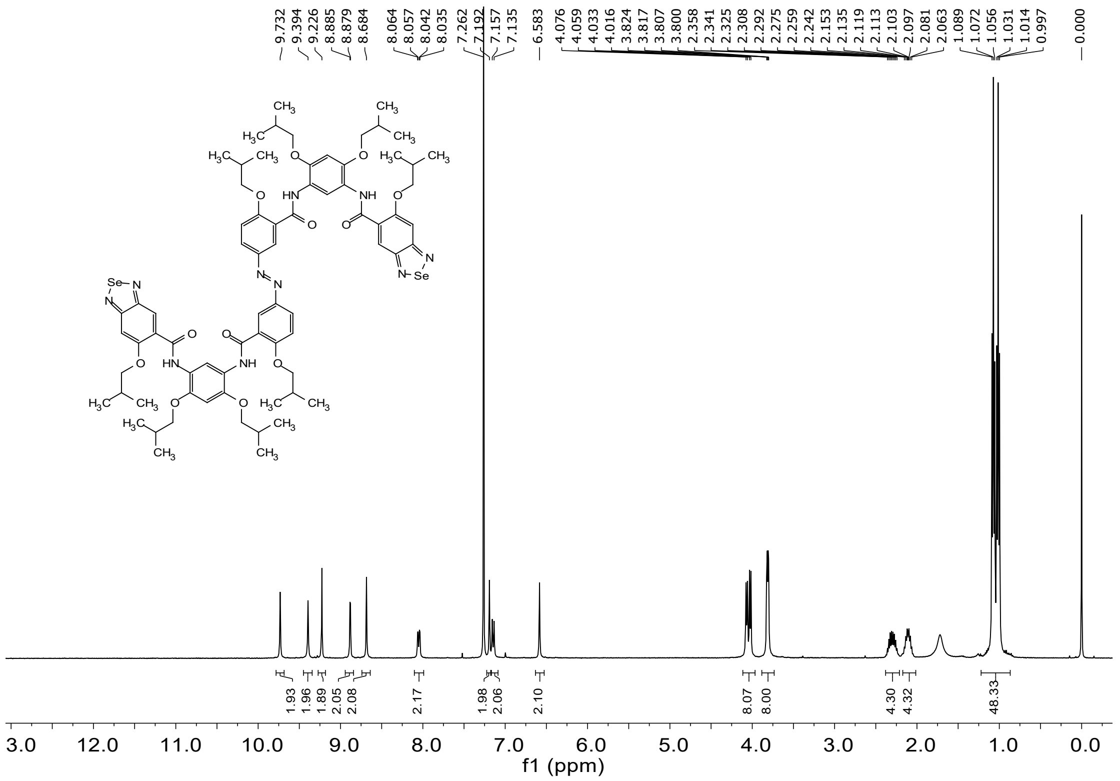

Figure S22. ^{1}H NMR (400 MHz, $CDCl_3$) spectrum of compound **3** at 25 °C.

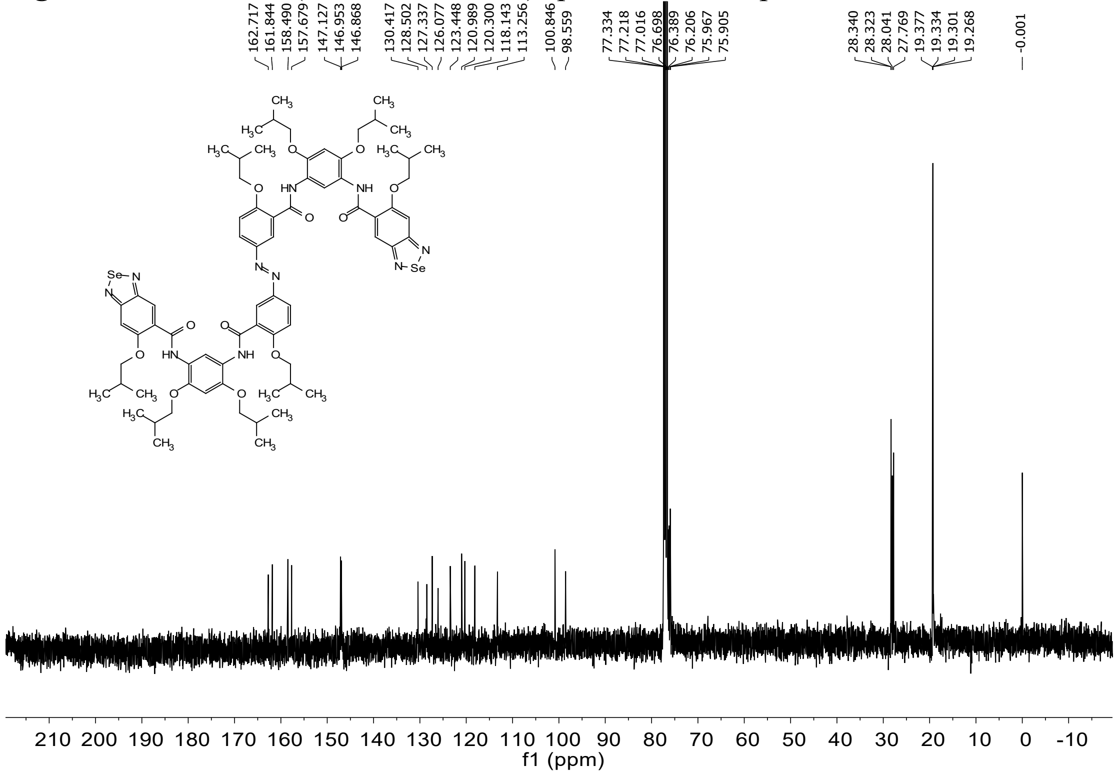

Figure S23. ^{13}C NMR (100 MHz, $CDCl_3$) spectrum of compound **3** at 25 °C.

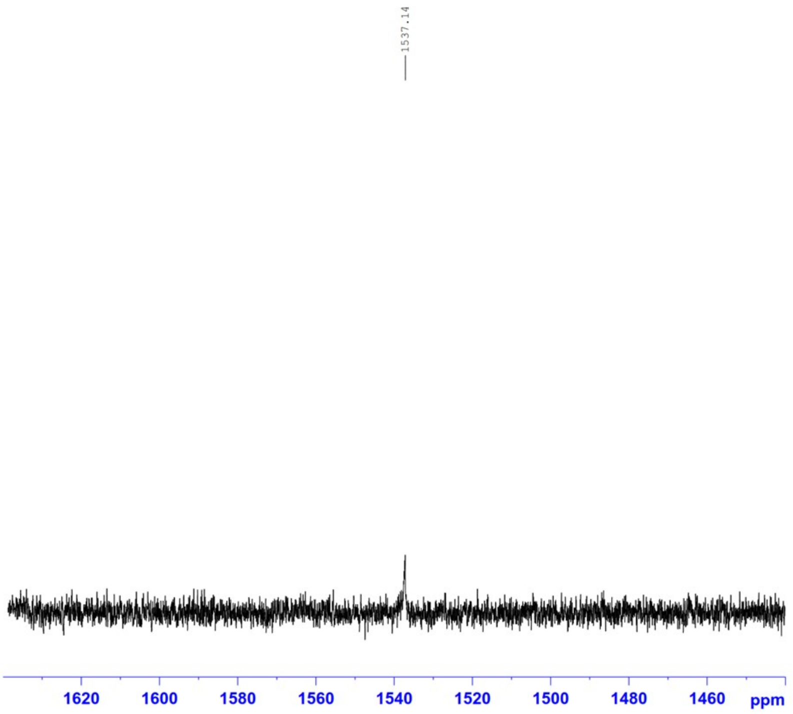

Figure S24. ^{77}Se NMR (76.3 MHz, $CDCl_3$) spectrum of compound **3** at 25 °C.

Single Mass Analysis
Tolerance = 5.0 mDa / DBE: min = -1.5, max = 50.0
Element prediction: Off
Number of isotope peaks used for i-FIT = 3

Monoisotopic Mass, Even Electron Ions
37 formula(e) evaluated with 1 results within limits (up to 50 best isotopic matches for each mass)
Elements Used:
C: 0-18 H: 0-18 N: 0-2 O: 0-6 Na: 0-1

20230501-1-3 49 (0.241)
1: TOF MS ES+

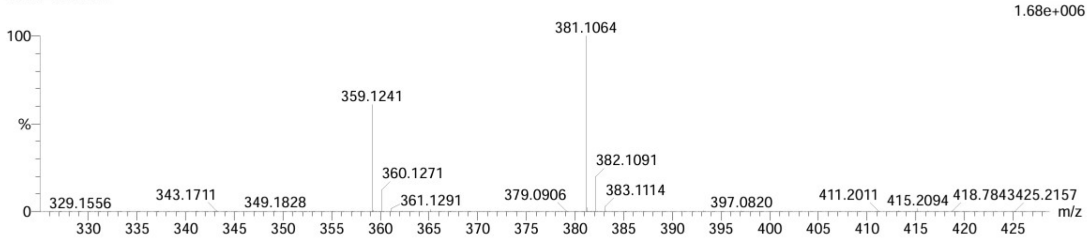

Minimum:				-1.5				
Maximum:		5.0	10.0	50.0				

Mass	Calc. Mass	mDa	PPM	DBE	i-FIT	Norm	Conf(%)	Formula
381.1064	381.1063	0.1	0.3	10.5	638.3	n/a	n/a	C18 H18 N2 O6 Na

Figure S25. HR-MS (ESI) of compound **5**.

Single Mass Analysis
Tolerance = 5.0 mDa / DBE: min = -1.5, max = 50.0
Element prediction: Off
Number of isotope peaks used for i-FIT = 3

Monoisotopic Mass, Even Electron Ions
17 formula(e) evaluated with 1 results within limits (up to 50 best isotopic matches for each mass)
Elements Used:
C: 0-16 H: 0-15 N: 0-2 O: 0-6
20230501-1-2 381 (1.644)
1: TOF MS ES+

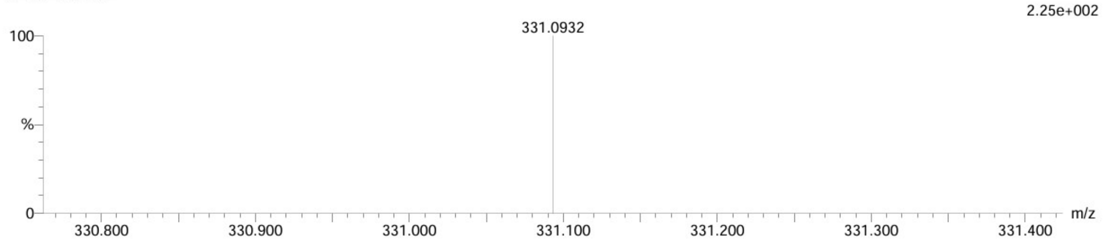

Minimum:				-1.5				
Maximum:		5.0	10.0	50.0				
Mass	Calc. Mass	mDa	PPM	DBE	i-FIT	Norm	Conf(%)	Formula
331.0932	331.0930	0.2	0.6	10.5	19.5	n/a	n/a	C16 H15 N2 O6

Figure S26. HR-MS (ESI) of compound **6**.

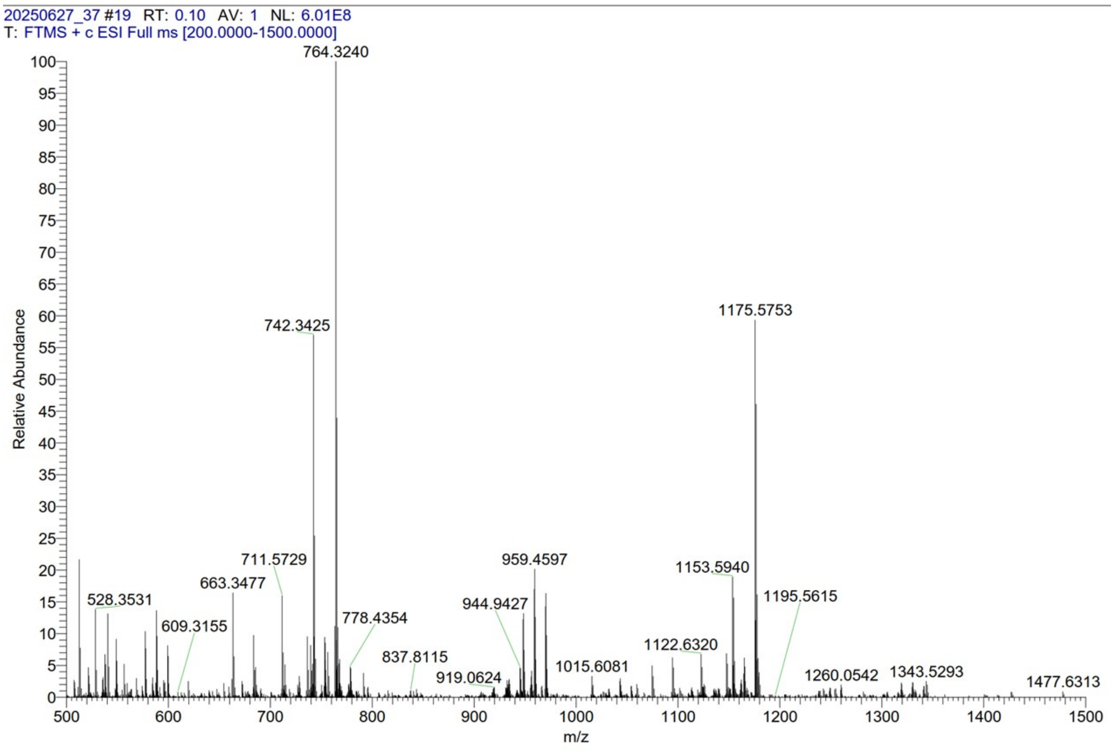

Figure S27. HR-MS (ESI) of compound **1**.

Single Mass Analysis
Tolerance = 5.0 mDa / DBE: min = -1.5, max = 50.0
Element prediction: Off
Number of isotope peaks used for i-FIT = 3

Monoisotopic Mass, Even Electron Ions
354 formula(e) evaluated with 1 results within limits (up to 50 best isotopic matches for each mass)
Elements Used:
C: 12-12 H: 16-16 N: 0-100 O: 0-100 Na: 0-2

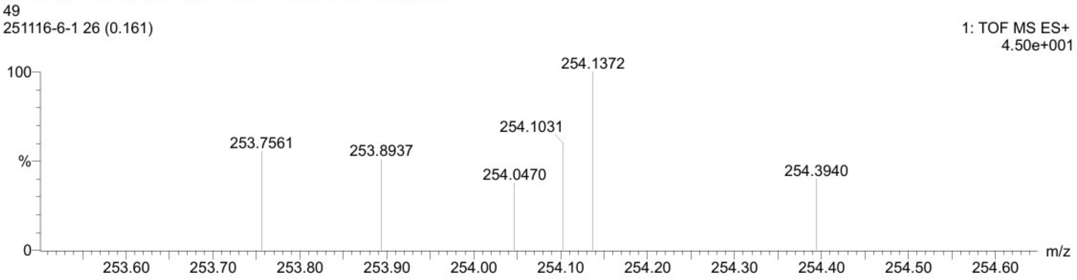

Minimum:				-1.5				
Maximum:		5.0	10.0	50.0				
Mass	Calc. Mass	mDa	PPM	DBE	i-FIT	Norm	Conf(%)	Formula
254.1031	254.1028	0.3	1.2	5.5	25.2	n/a	n/a	C12 H16 N O5

Figure S28. HR-MS (ESI) of compound **9**.

Single Mass Analysis
Tolerance = 5.0 mDa / DBE: min = -1.5, max = 50.0
Element prediction: Off
Number of isotope peaks used for i-FIT = 3

Monoisotopic Mass, Even Electron Ions
320 formula(e) evaluated with 1 results within limits (up to 50 best isotopic matches for each mass)
Elements Used:
C: 13-13 H: 20-20 N: 0-100 O: 0-100 Na: 0-2

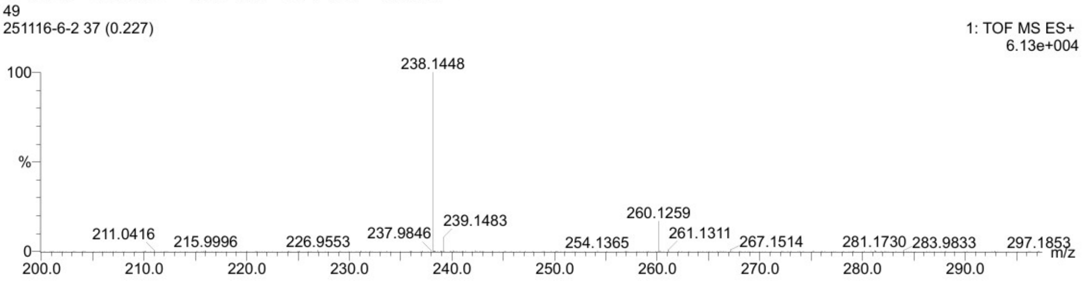

Minimum:				-1.5				
Maximum:		5.0	10.0	50.0				
Mass	Calc. Mass	mDa	PPM	DBE	i-FIT	Norm	Conf(%)	Formula
238.1448	238.1443	0.5	2.1	4.5	267.0	n/a	n/a	C13 H20 N O3

Figure S29. HR-MS (ESI) of compound **10**.

Single Mass Analysis
Tolerance = 5.0 mDa / DBE: min = -1.5, max = 50.0
Element prediction: Off
Number of isotope peaks used for i-FIT = 3

Monoisotopic Mass, Even Electron Ions
65 formula(e) evaluated with 1 results within limits (up to 50 best isotopic matches for each mass)
Elements Used:
C: 0-26 H: 0-35 N: 0-4 O: 0-6 Na: 0-1
20230728-2-lf-2-56 150 (0.605)
1: TOF MS ES+

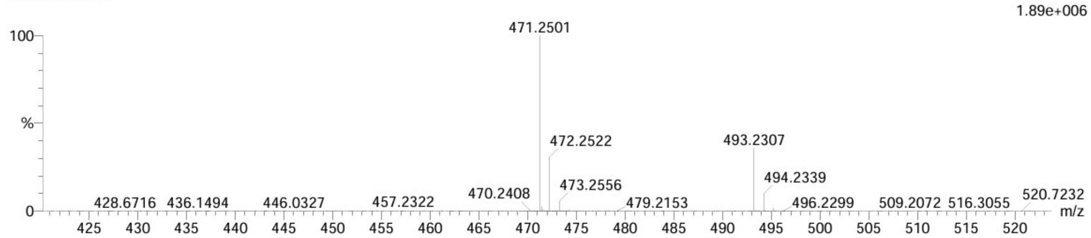

Minimum:				-1.5				
Maximum:		5.0	10.0	50.0				
Mass	Calc. Mass	mDa	PPM	DBE	i-FIT	Norm	Conf(%)	Formula
471.2501	471.2495	0.6	1.3	10.5	714.2	n/a	n/a	C26 H35 N2 O6

Figure S30. HR-MS (ESI) of compound **11**.

Single Mass Analysis
Tolerance = 5.0 mDa / DBE: min = -1.5, max = 50.0
Element prediction: Off
Number of isotope peaks used for i-FIT = 3

Monoisotopic Mass, Even Electron Ions
64 formula(e) evaluated with 1 results within limits (up to 50 best isotopic matches for each mass)
Elements Used:
C: 0-22 H: 0-27 N: 0-4 O: 0-6 Na: 0-1
20230728-2-lcg-1 102 (0.418)
1: TOF MS ES+

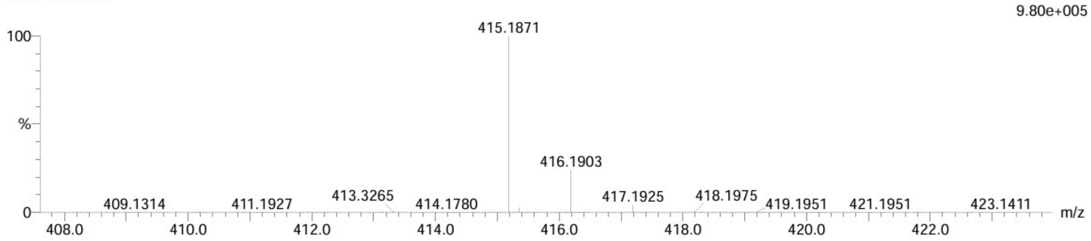

Minimum:				-1.5				
Maximum:		5.0	10.0	50.0				
Mass	Calc. Mass	mDa	PPM	DBE	i-FIT	Norm	Conf(%)	Formula
415.1871	415.1869	0.2	0.5	10.5	549.3	n/a	n/a	C22 H27 N2 O6

Figure S31. HR-MS (ESI) of compound **12**.

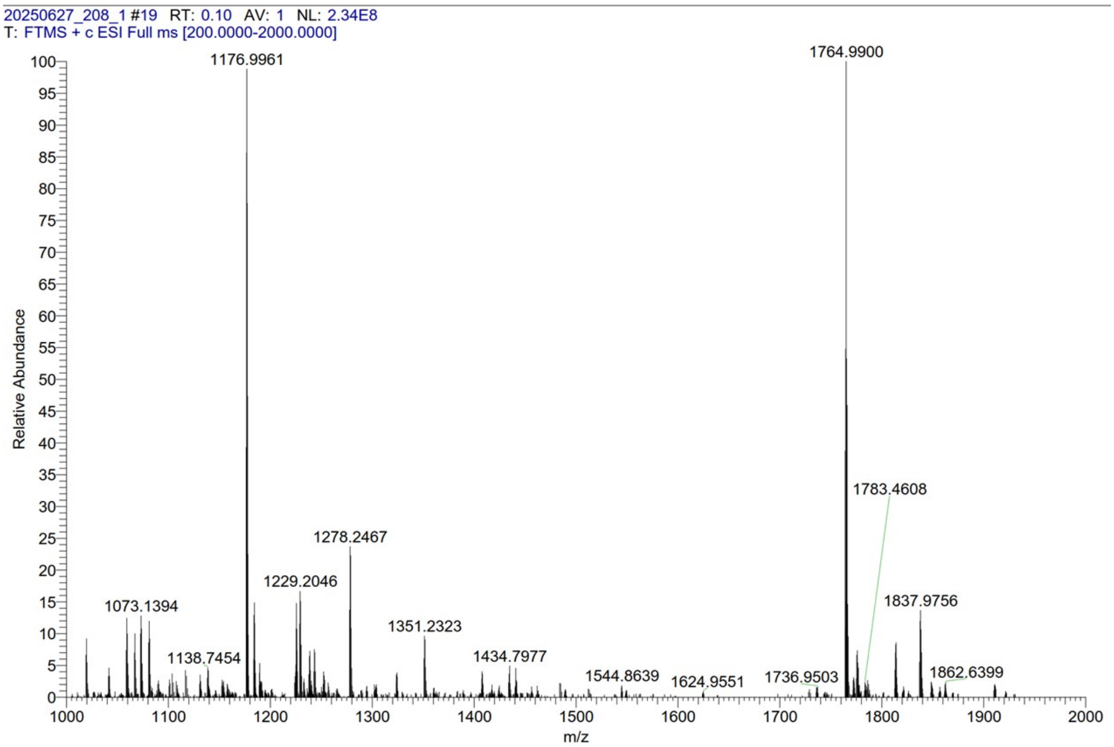

Figure S32. HR-MS (ESI) of compound **2**.

Tolerance = 5.0 PPM / DBE: min = -1.5, max = 50.0
Element prediction: Off
Number of isotope peaks used for i-FIT = 3

Monoisotopic Mass, Even Electron Ions
171 formula(e) evaluated with 1 results within limits (up to 50 best isotopic matches for each mass)
Elements Used:
C: 60-60 H: 0-100 N: 0-6 O: 0-12 Na: 0-1
49
251116-6-3 77 (0.445)

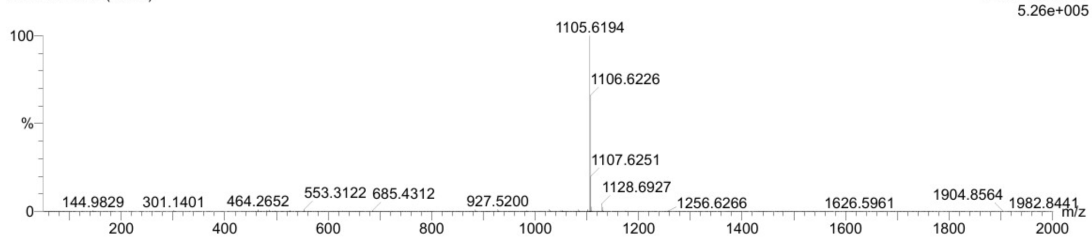

Minimum:	80.00				-1.5		
Maximum:	100.00		5.0	5.0	50.0		
Mass	RA	Calc. Mass	mDa	PPM	DBE	i-FIT	Formula
1105.6194	100.00	1105.6201	-0.7	-0.6	20.5	486.4	C60 H86 N6 O12 Na

Figure S33. HR-MS (ESI) of compound **15**.

Monoisotopic Mass, Even Electron Ions
356 formula(e) evaluated with 1 results within limits (up to 50 best isotopic matches for each mass)
Elements Used:
C: 0-50 H: 0-73 N: 0-6 O: 0-8 Na: 0-1 Cl: 0-2
20230728-2-lcg-3 95 (0.391)
1: TOF MS ES+

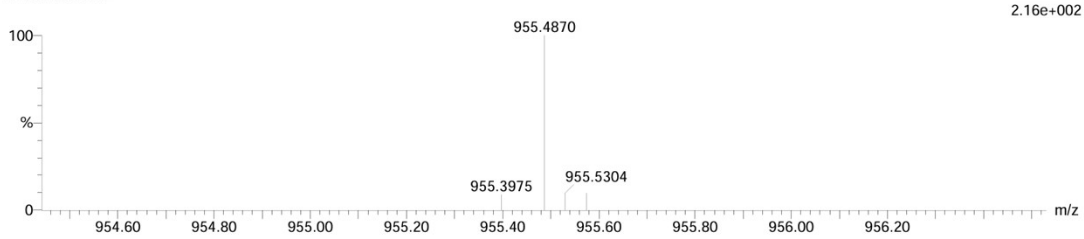

Minimum:				-1.5				
Maximum:		5.0	10.0	50.0				
Mass	Calc. Mass	mDa	PPM	DBE	i-FIT	Norm	Conf(%)	Formula
955.4870	955.4867	0.3	0.3	16.5	34.5	n/a	n/a	C50 H73 N6 O8 Cl2

Figure S34. HR-MS (ESI) of compound **16**.

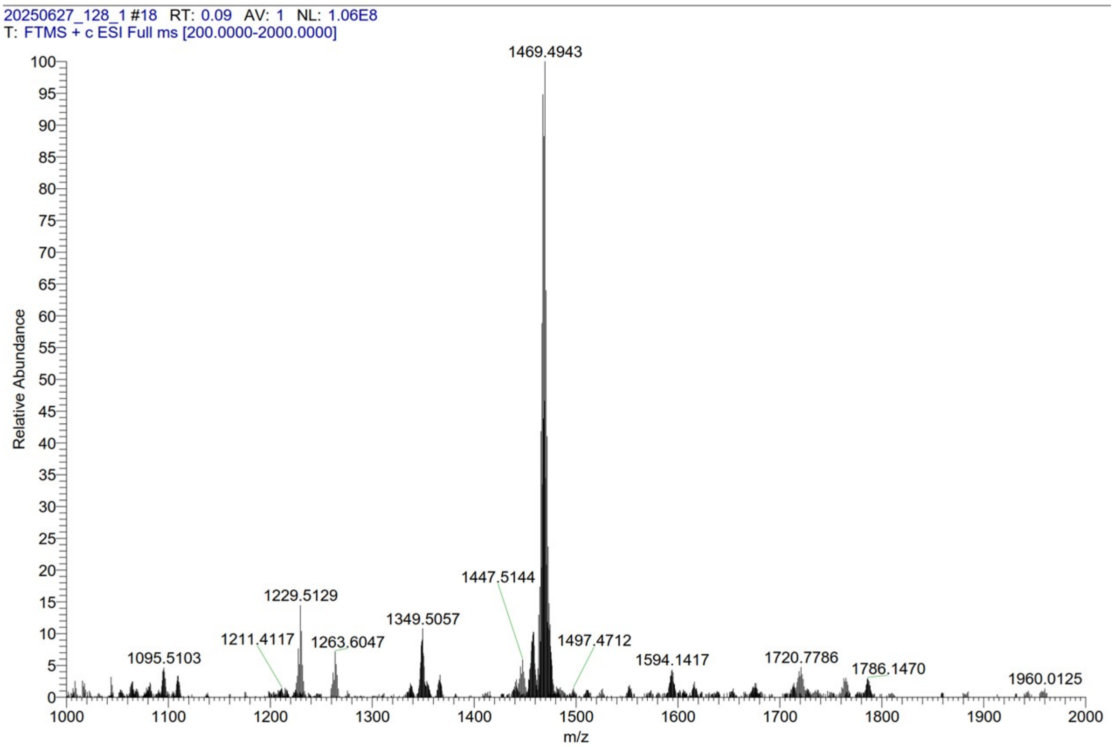

Figure S35. HR-MS (ESI) of compound **3**.

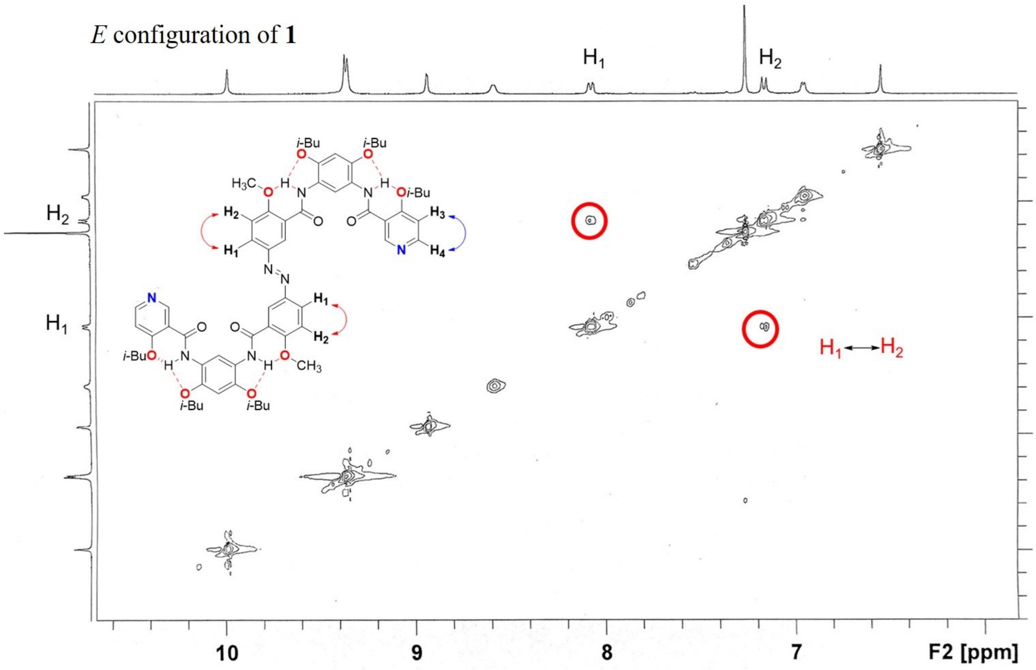

Figure S36. ROESY spectrum of compound **1** in $CDCl_3$ (3.0 mM) before 365 nm UV light irradiation.

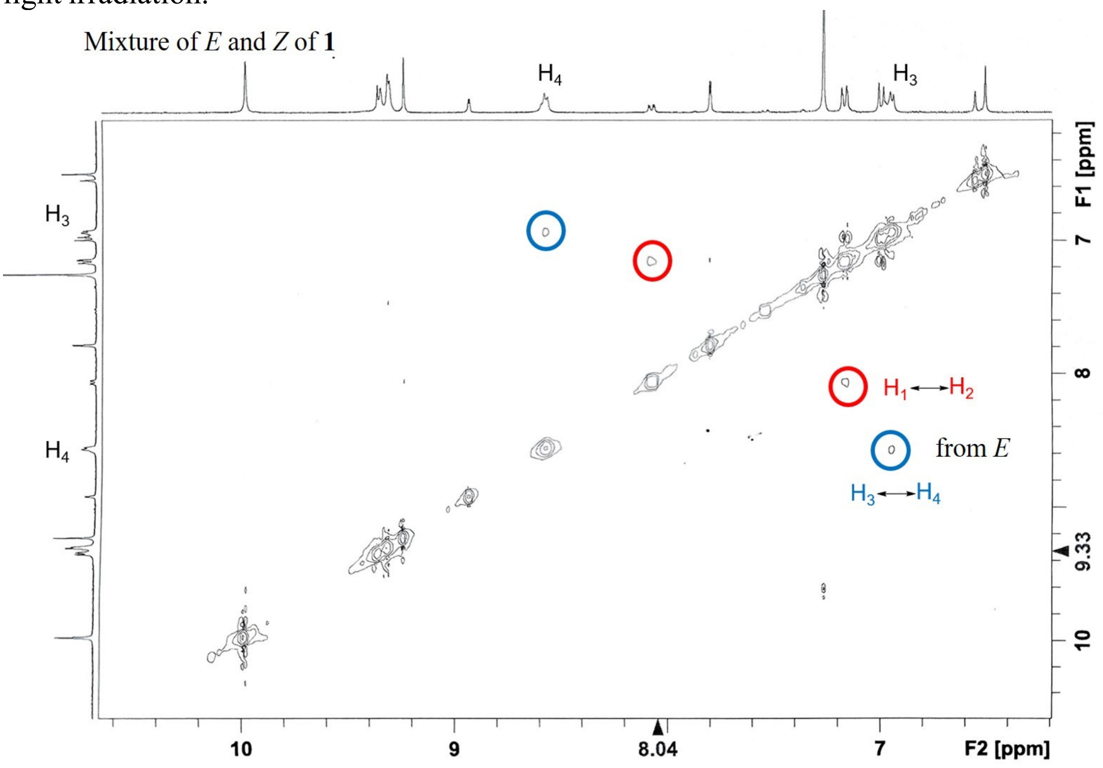

Figure S37. ROESY spectrum of compound **1** in $CDCl_3$ (3.0 mM) after 365 nm UV light irradiation.

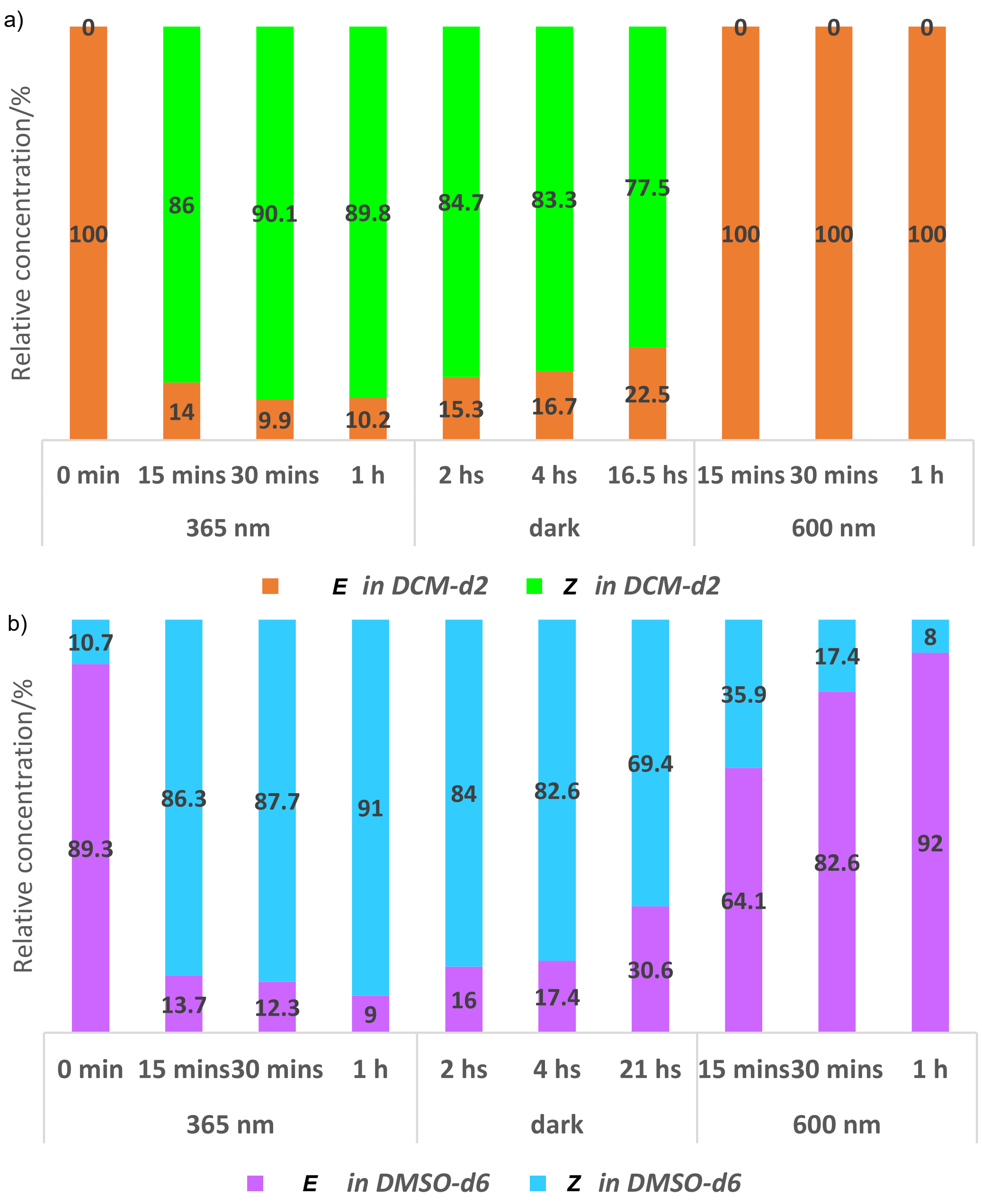

Figure S38. The interconversion between the *E* (*trans*) and *Z* (*cis*) configurations of compound **2** in CD_2Cl_2 (a) and DMSO-d_6 (b). (The percentages of the *E* (*trans*) and *Z* (*cis*) configurations of compound **2** were calculated by ^{1}H NMR.

Table S1 Integration of key cross-peaks of *E* and *Z* configurations of compound **2** in DMSO-d_6.

	Integral (Normalized)						
	**H_4-H_5**	**H_1-H_2**	**H_2-H_3**	**H_3-H_{11}**	**H_{10}-H_{11}/H_{12}**	**H_6-H_{13}**	**H_7-H_{13}**
E-**2**	1.0	1.257	-	0.1124	0.1103	0.0252	0.0866
Z-**2**	1.0	0.5252	0.1477	0.0080	0.1680	0.1045	0.1171

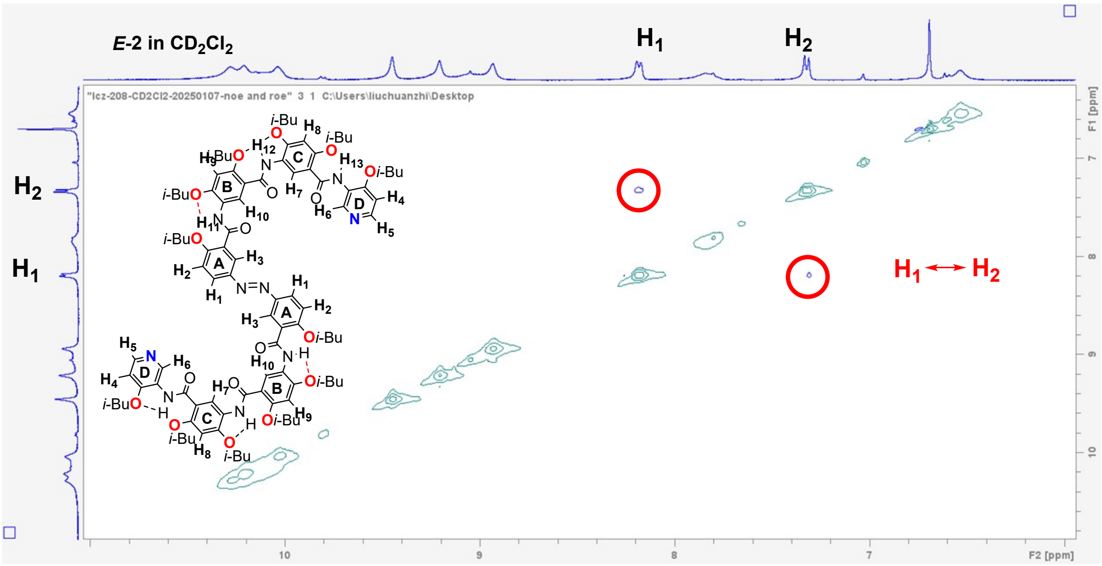

Figure S39. 2D ROE spectra of the *E* configuration of compound **2** in CD_2Cl_2 (5.0 mM).

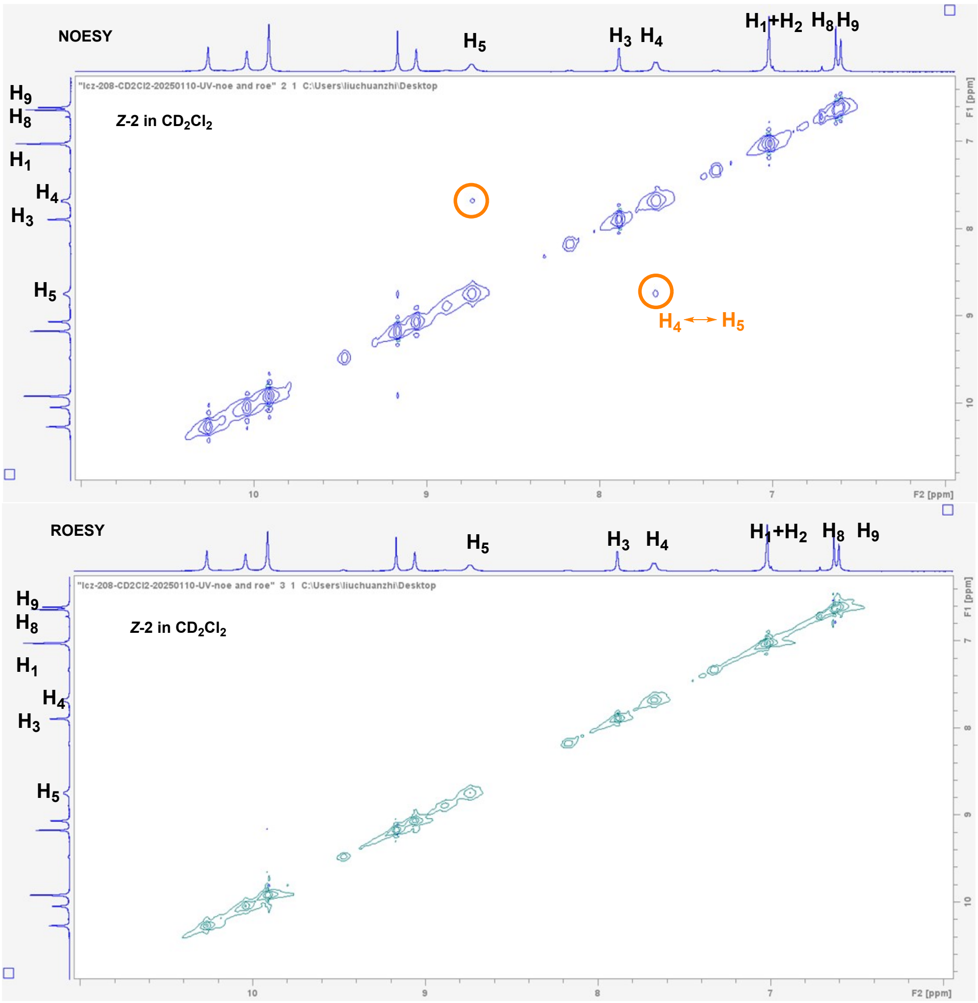

Figure S40. 2D NOE and ROE spectra of the *Z* configuration of compound **2** in CD_2Cl_2 (5.0 mM).

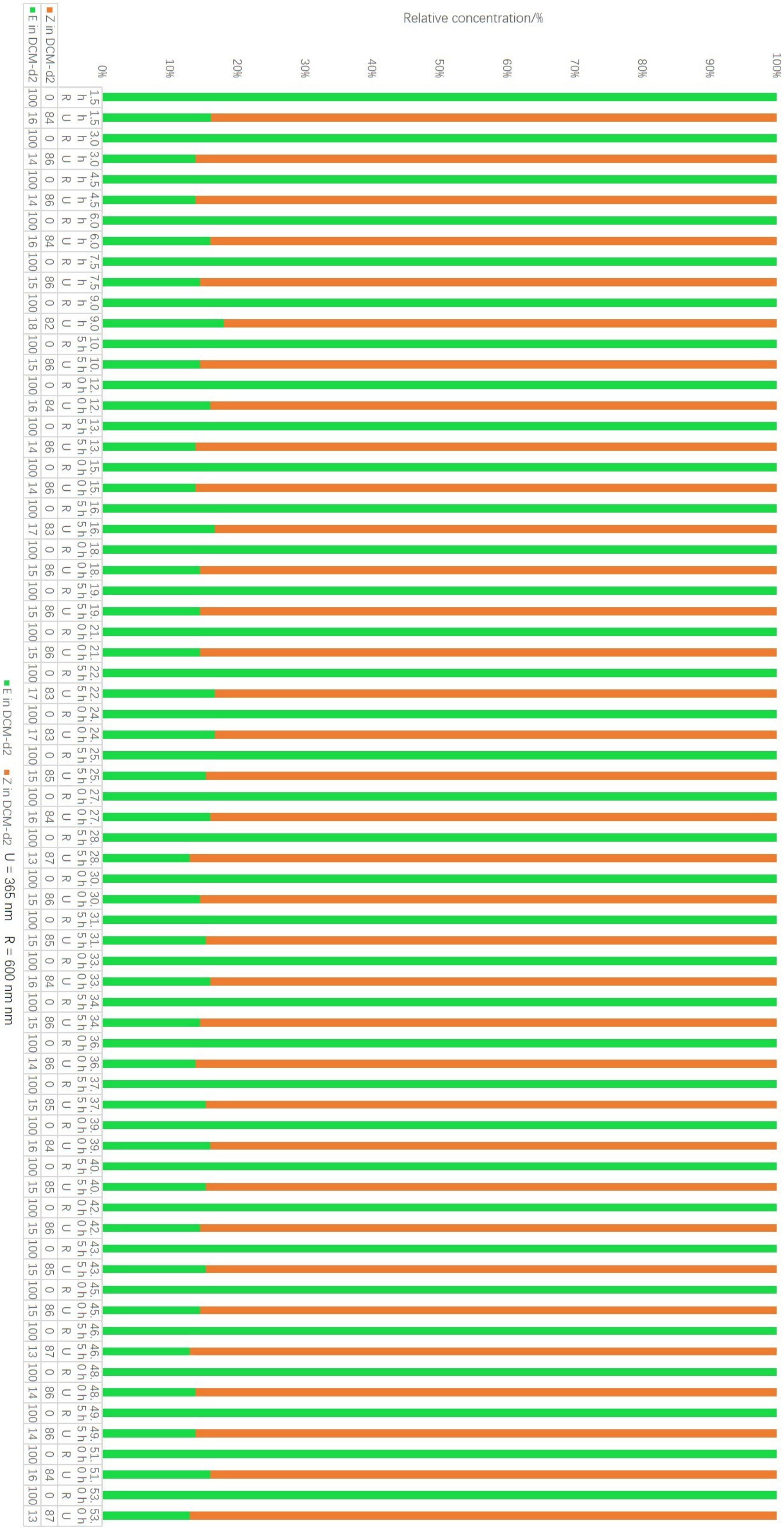

Figure S41. The reversible cyclic isomerization between *E* and *Z* configurations. The intercepted part of switching cycles of compound **2** in CD_2Cl_2. (The percentages of the *E* and *Z* configurations of compound **2** were calculated by ^{1}H NMR. The total duration of 600 nm red light irradiation was 53 hours, and the total duration of 365 nm UV irradiation was also 53 hours, with an irradiation time interval of 1.5 hours.)

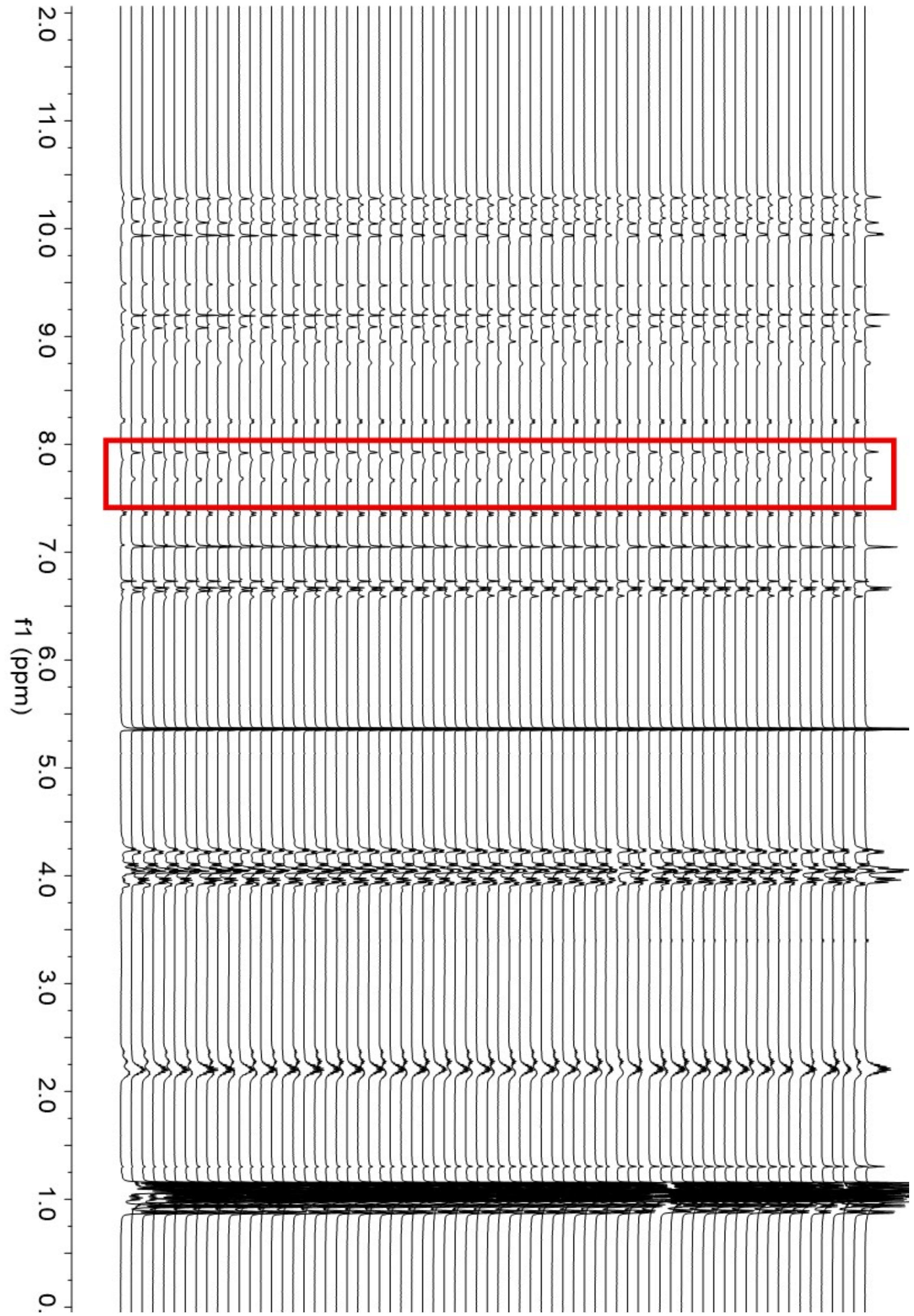

Figure S42. The reversible cyclic isomerization between *E* and *Z* configurations. The intercepted part of switching cycles of compound **2** in CD_2Cl_2 (5.0 mM). (The total duration of 600 nm red light irradiation was 53 hours, and the total duration of 365 nm UV irradiation was also 53 hours, with an irradiation time interval of 1.5 hours.)

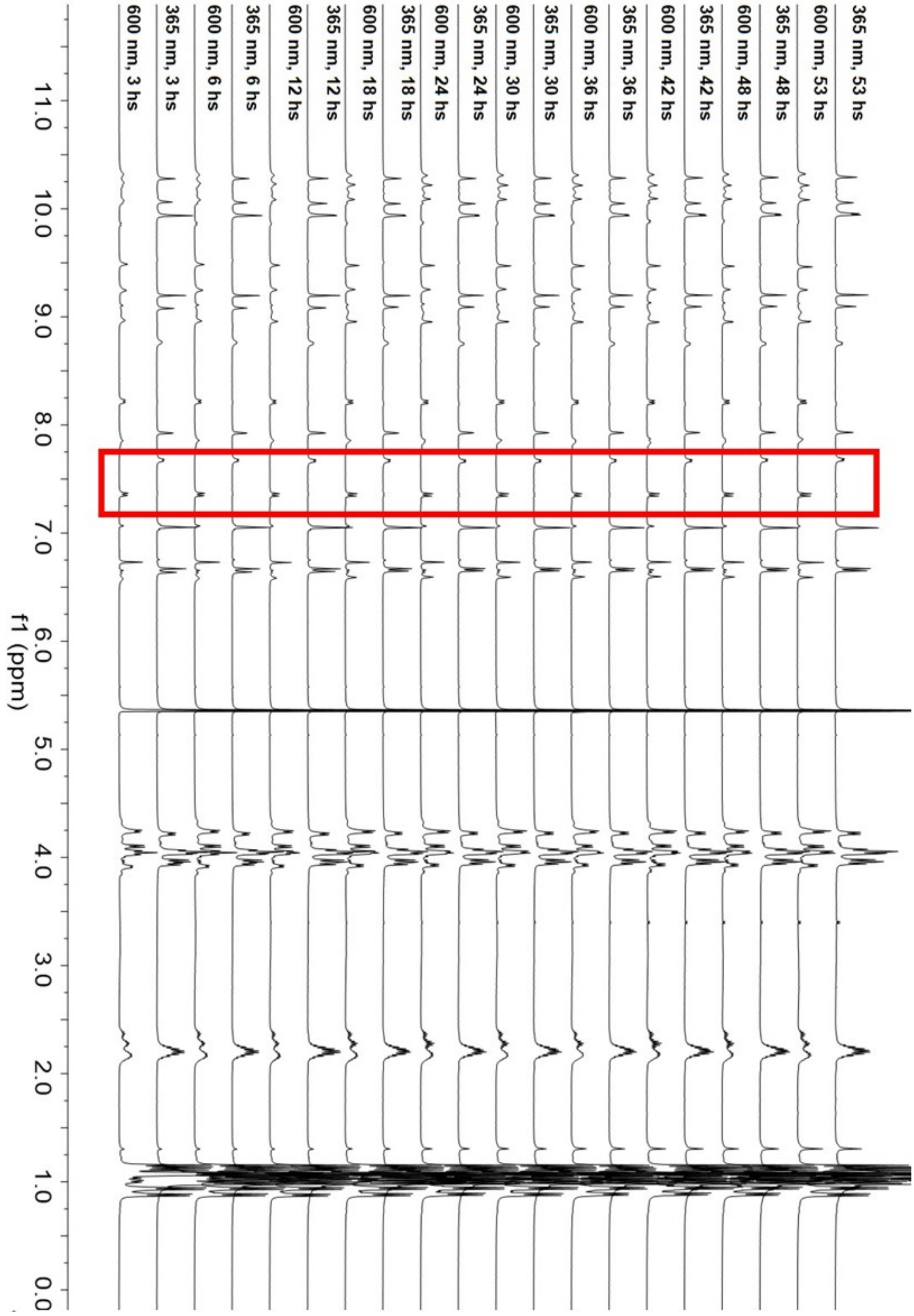

Figure S43. The reversible cyclic isomerization between *E* and *Z* configurations. The intercepted part of switching cycles of compound **2** in CD_2Cl_2 (5.0 mM). (Partial NMR data from the irradiation time period were captured.)

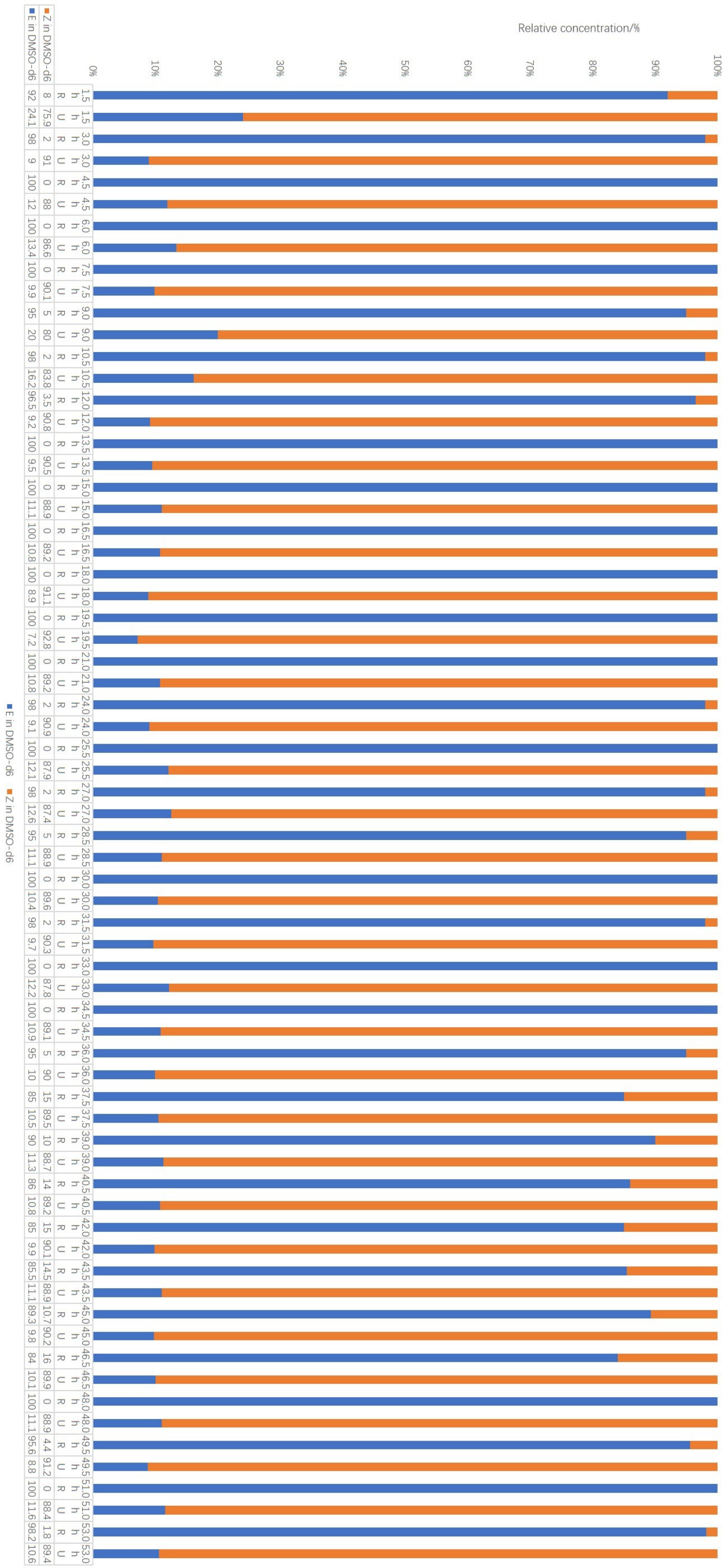

Figure S44. The reversible cyclic isomerization between *E* and *Z* configurations. The intercepted part of switching cycles of compound **2** in DMSO-d_6 (5.0 mM). (The percentages of the *E* and *Z* configurations of compound **2** were calculated by ^{1}H NMR. The total duration of 600 nm red light irradiation was 53 hours, and the total duration of 365 nm UV irradiation was also 53 hours, with an irradiation time interval of 1.5 hours.)

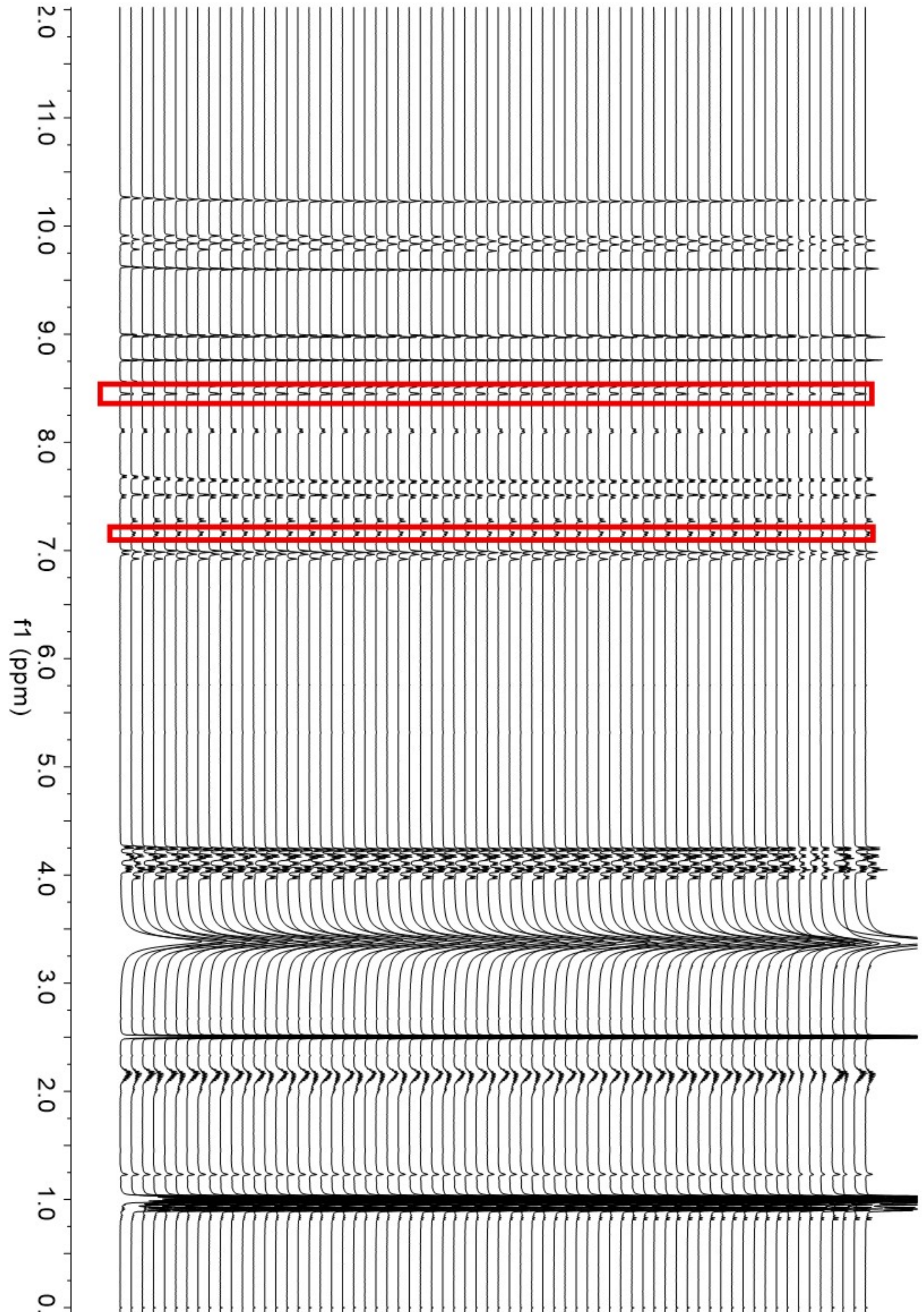

Figure S45. The reversible cyclic isomerization between *E* and *Z* configurations. The intercepted part of switching cycles of compound **2** in DMSO-d_6 (5.0 mM). (The total duration of 600 nm red light irradiation was 53 hours, and the total duration of 365 nm UV irradiation was also 53 hours, with an irradiation time interval of 1.5 hours.)

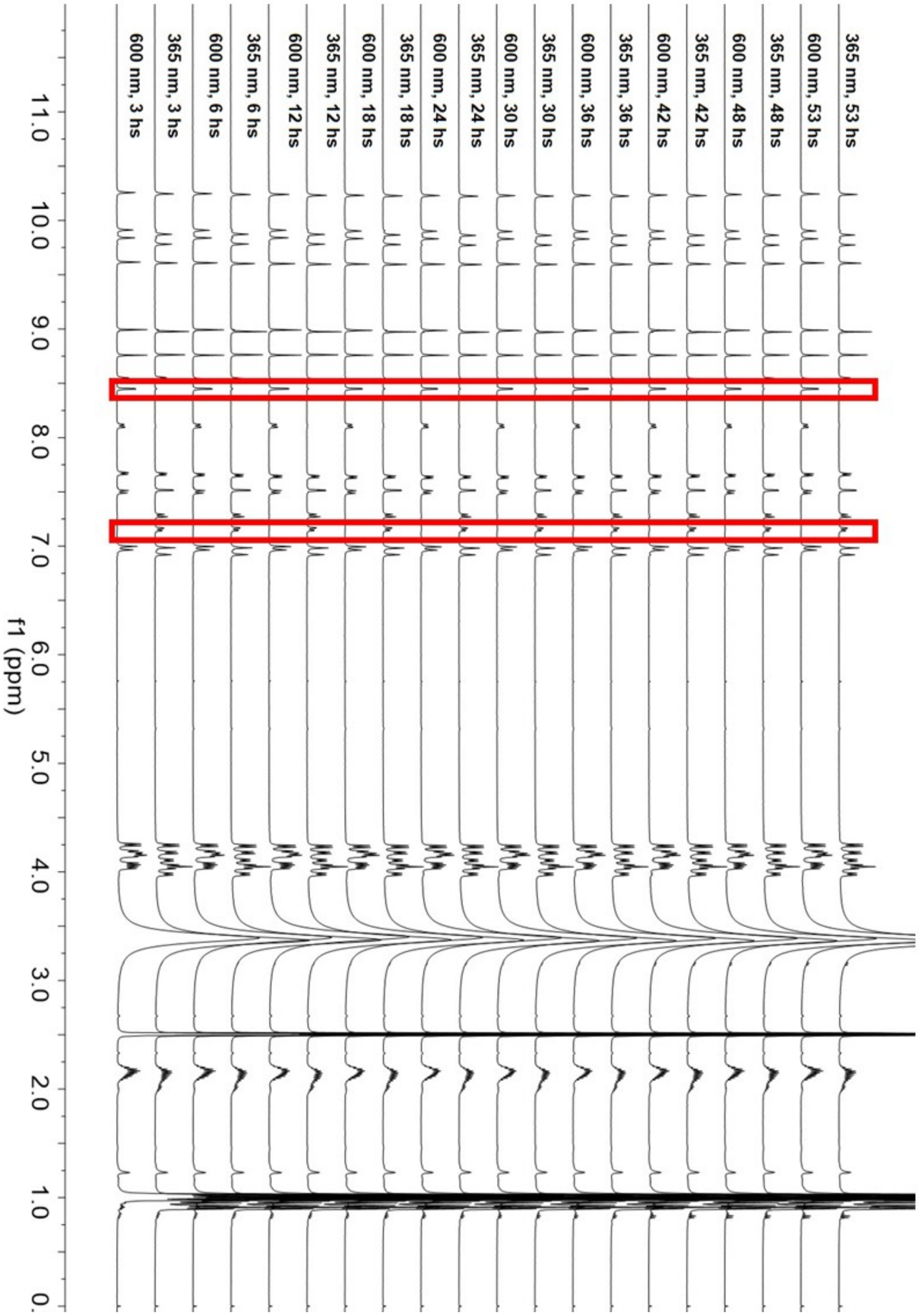

Figure S46. The reversible cyclic isomerization between *E* and *Z* configurations. The intercepted part of switching cycles of compound **2** in DMSO-d_6 (5.0 mM). (Partial NMR data from the irradiation time period were captured.)

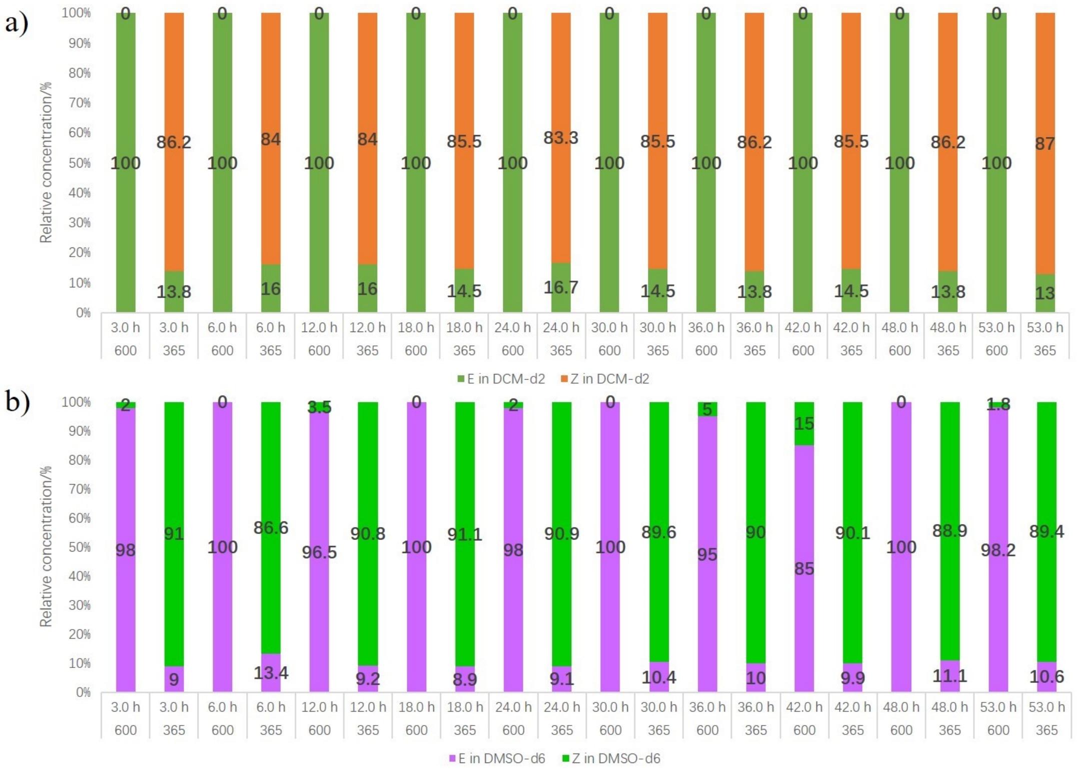

Figure S47 Intercepted part of the switching cycles of compound **2** in CD_2Cl_2 (a) (5.0 mM) and DMSO-d_6 (b) (5.0 mM). (The percentages of the *E* and *Z* configurations of compound **2** were calculated by ^{1}H NMR. The detailed ^{1}H NMR data and comprehensive bar chart are presented in Figs. S41, S44.

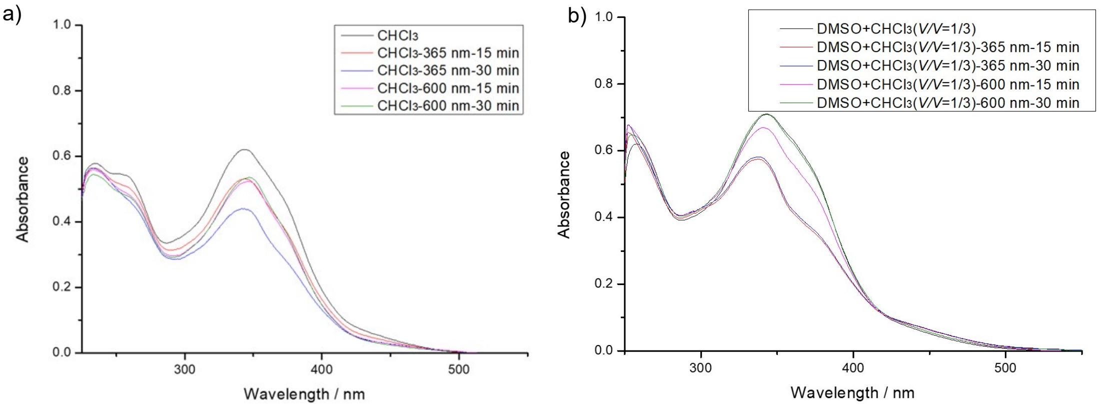

Figure S48. The UV-vis absorption spectra of compound **3** under varying irradiation durations at 365 nm and 600 nm. a) The spectra of dichloromethane as the solvent. b) The spectra of DMSO as the solvent. (Sample concentration of compound **3** in $CHCl_3$ and $CHCl_3$/DMSO with a volume ratio of 3:1 was1.1 × 10^{-6} mol/L in both cases.)

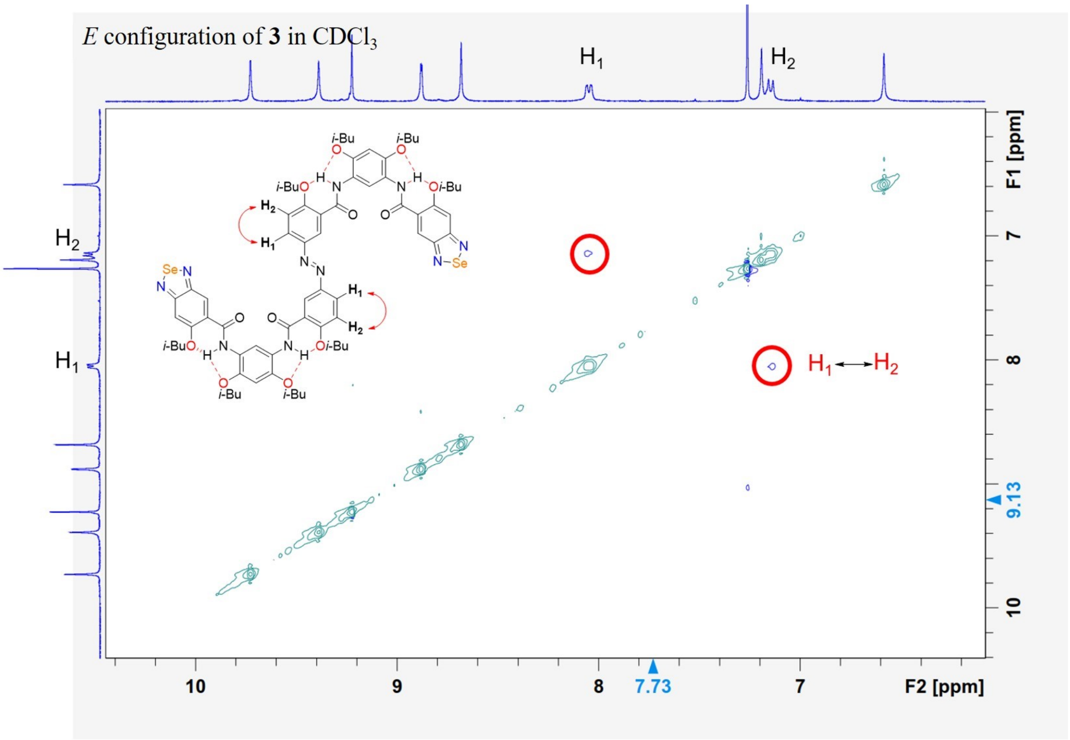

Figure S49. ROESY spectrum of compound **3** (*E* conformation) in $CDCl_3$ (5.0 mM) before 365 nm UV light irradiation.

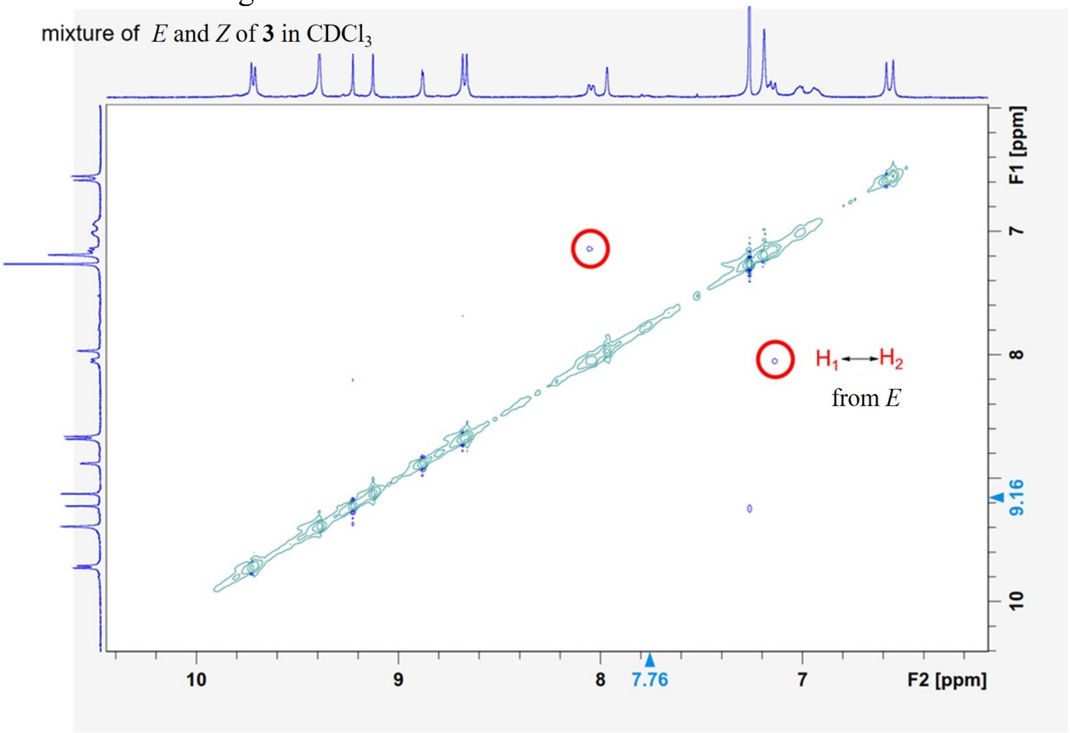

Figure S50. ROESY spectrum of compound **3** (mixture of *E* and *Z* conformations) in $CDCl_3$ (5.0 mM) after 365 nm UV light irradiation.

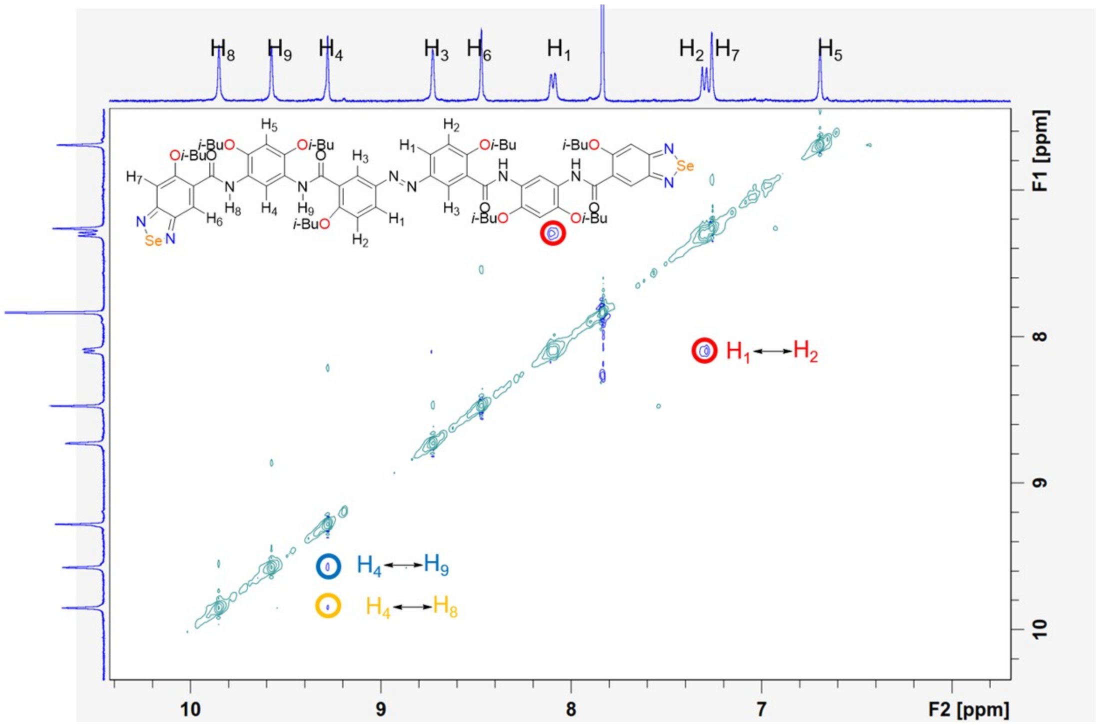

Figure S51. ROESY spectrum of compound **3** (*E* conformation) in $CDCl_3$/DMSO-d_6 (*V/V*: 3/1) (5.0 mM) before 365 nm UV light irradiation.

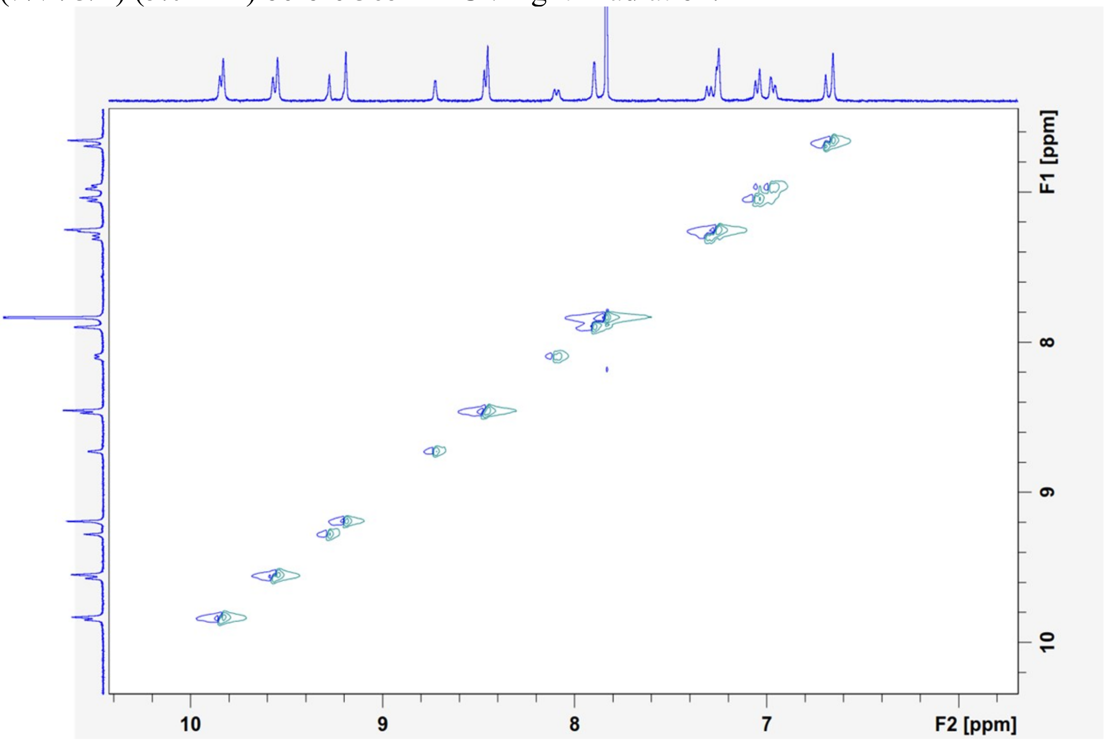

Figure S52. ROESY spectrum of compound **3** (mixture of *E* and *Z* conformations) in $CDCl_3$/DMSO-d_6 (*V/V*: 3/1) (5.0 mM) after 365 nm UV light irradiation.

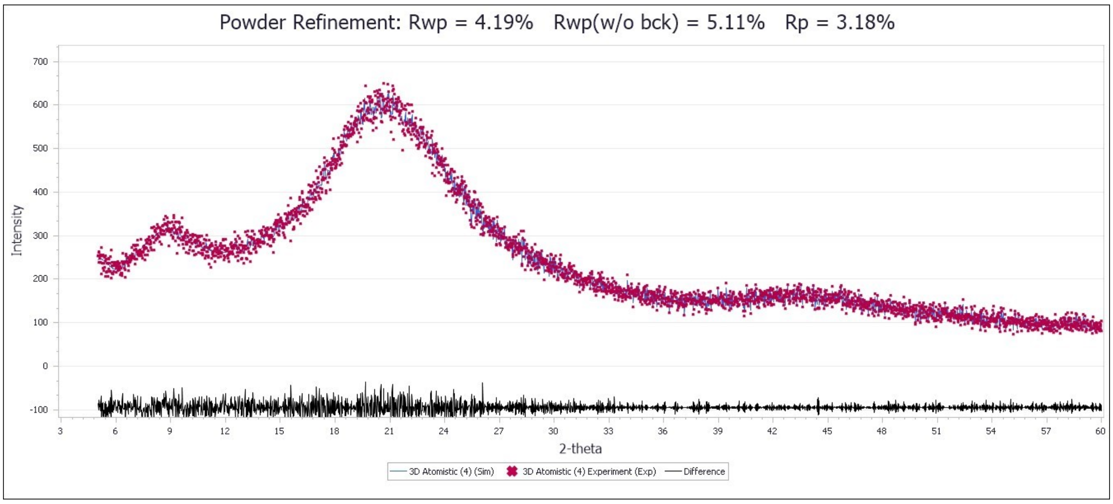

Figure S53. PXRD data of compound *Z*-**3** and its comparison with the computational model.

As shown in Figure S52, the experimental PXRD pattern of compound **3** (red dots) was compared with the simulated pattern derived from our proposed molecular packing model (blue line). The Rwp value of 4.19% (and Rwp(w/o bck) = 5.11%, Rp = 3.18%) confirms a good quantitative agreement between the experimental and calculated profiles, which was a strong indicator of the validity of the packing model.

Peak position matching: All major diffraction peaks observed in the experimental pattern were accurately reproduced by the simulation across the entire 2θ range (3°–60°). This precise alignment of peak positions confirms that the unit cell parameters and overall crystal lattice structure derived from our model were consistent with the real solid-state arrangement.

Intensity profile matching: The relative intensities of the peaks, including the dominant peak at ~20° 2θ and the secondary features at ~9° and ~30°, were well-reproduced by the simulation. Minor discrepancies in peak intensities, as reflected in the difference curve (black line), were attributed to small amounts of structural disorder, preferred orientation of the powder particles, or trace amounts of residual *Z* configuration in the photostationary state, all of which were common in powder diffraction measurements and do not compromise the integrity of the packing model.

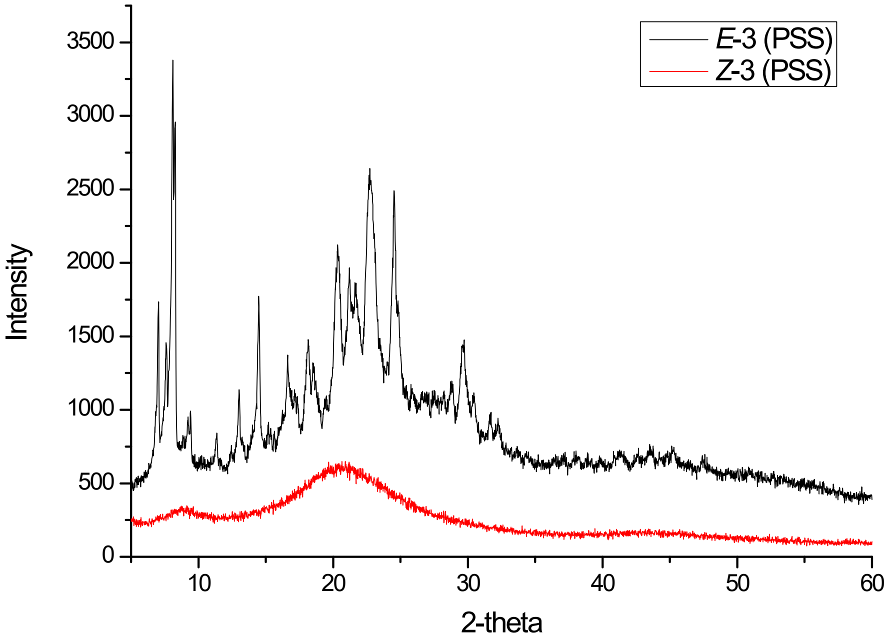

Figure S54. PXRD data of compound **3** (*E*-**3** (PSS) and *Z*-**3** (PSS)).

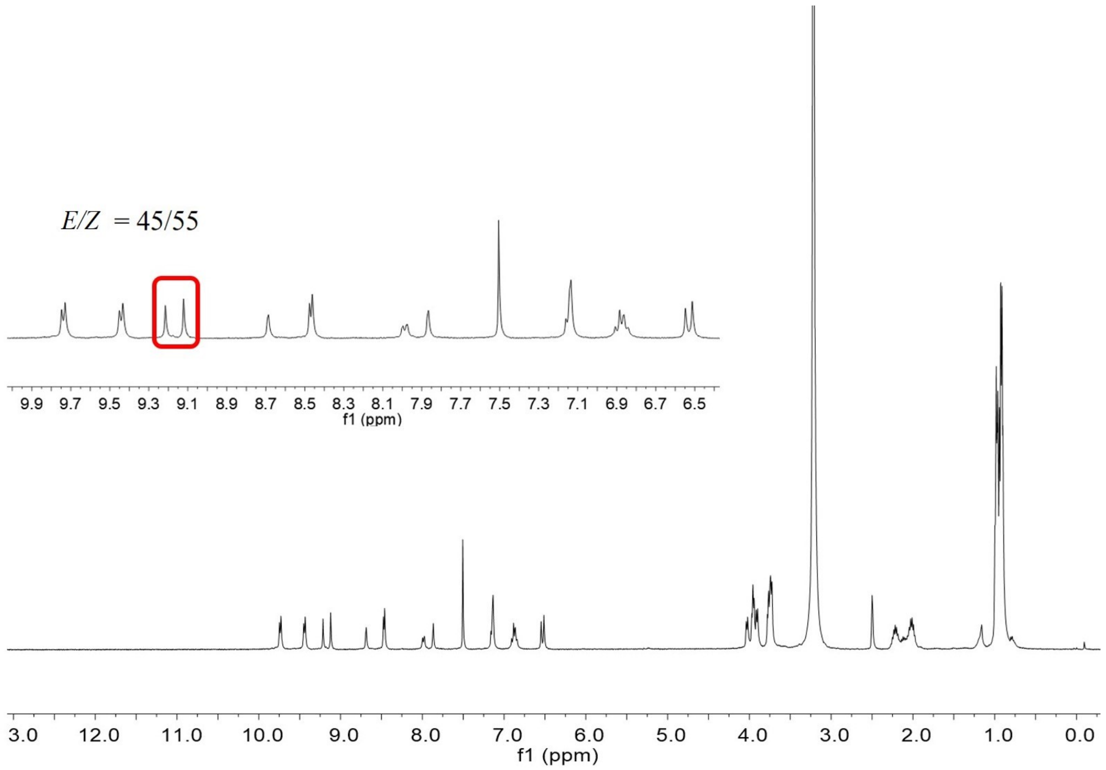

Figure S55. ^{1}HNMR data were acquired by dissolving the PXRD samples prepared under 365 nm light of compound **3** in $CDCl_3$/DMSO-d_6 (*V/V*: 3/1) (6.5 mM).

Quantum yield of compound 2 and 3

Photoluminescence measurements were carried out at room temperature on an FLS1000 fluorescence spectrophotometer. Samples were excited using a 450 W xenon lamp. Prior to UV irradiation at 365 nm, the emission and excitation spectra of compound **2** (in CH_2Cl_2 and DMSO) and compound **3** (in $CHCl_3$ and $CHCl_3$/DMSO with a volume ratio of 3:1) were recorded in the corresponding solvents. The quantum yield (QY) was calculated as follows, where the reference refers to the integrated emission intensity of the corresponding blank solvent without solute.

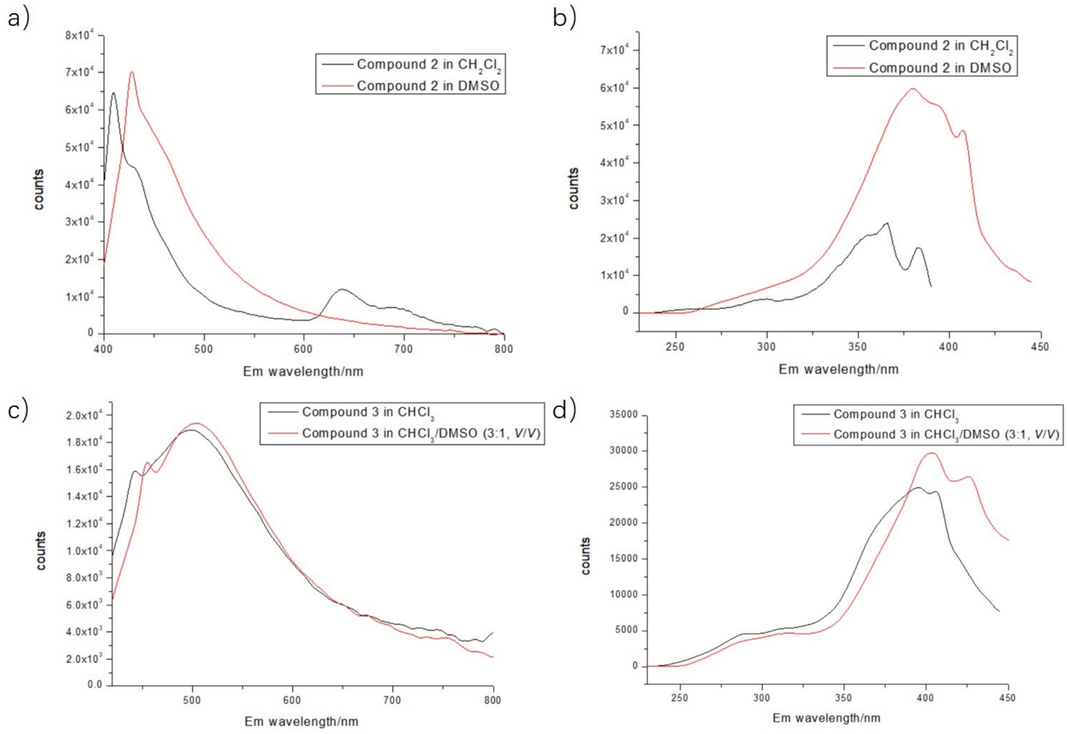

Figure S56. The excitation and emission spectra of compounds **2** and **3**. (a) Emission spectra of compound **2**. (b) Emission spectra of compounds **2**. CH_2Cl_2 (λex = 365 nm, λem = 410 nm), DMSO (λex = 375 nm, λem = 420 nm). (c) Emission spectra of compound **3**. (b) Emission spectra of compounds **3**. $CHCl_3$ (λex = 390 nm, λem = 500 nm), $CHCl_3$/DMSO (3:1 *V*/*V*) (λex = 406 nm, λem = 505 nm).

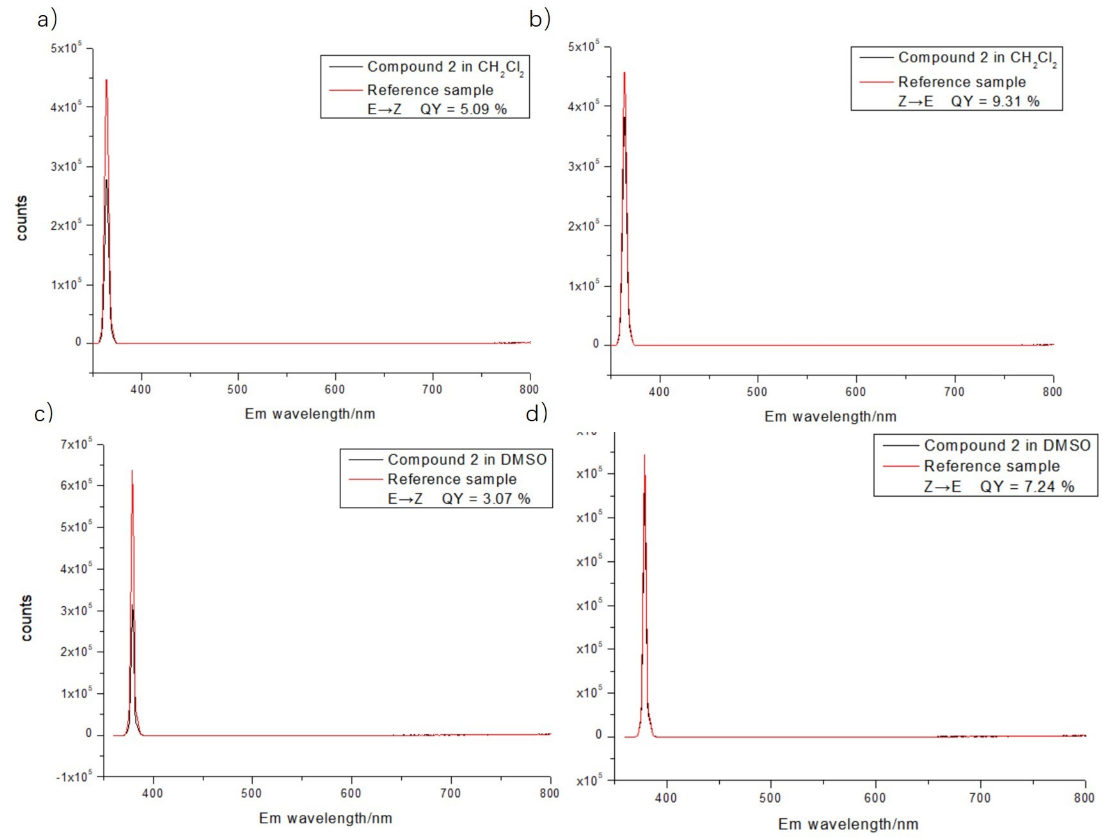

Figure S57. Quantum yield of *E* (*trans*-) ⇄ *Z* (*cis*-) photoisomerization of compound of **2**. (a), (b) Compound **2** in CH_2Cl_2. (c), (d) Compound **2** in DMSO.

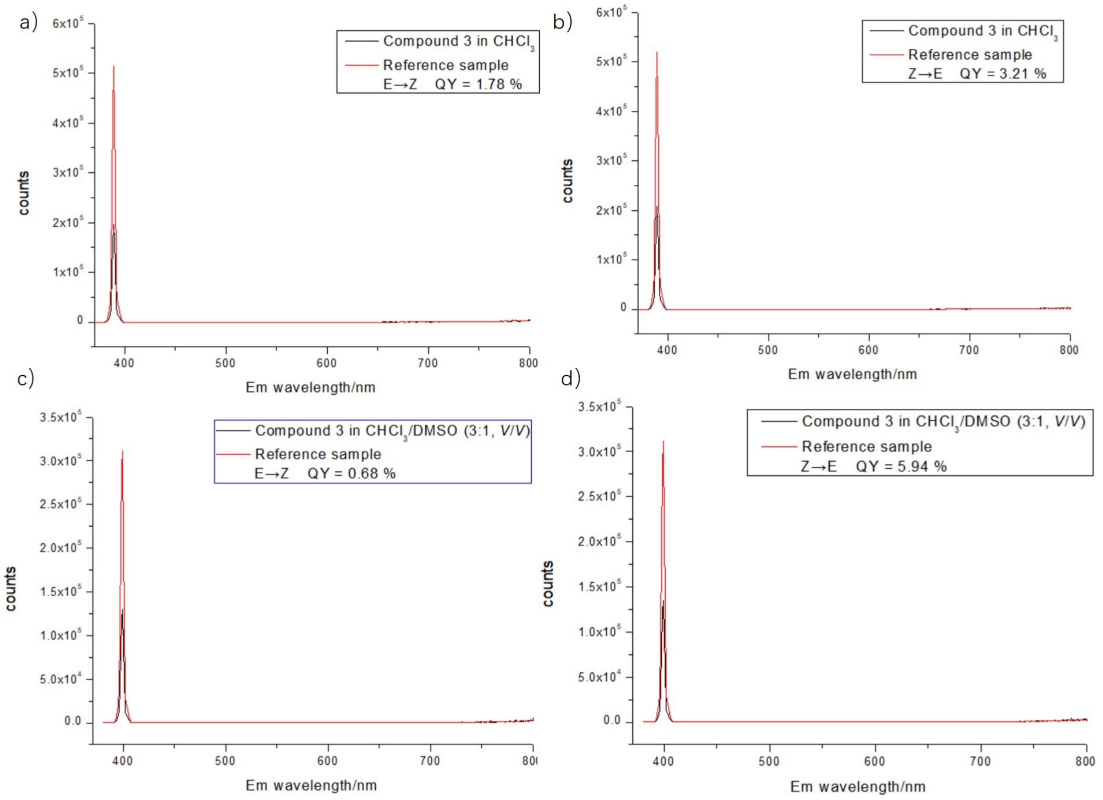

Figure S58. Quantum yield of *E* (*trans*-) ⇄ *Z* (*cis*-) photoisomerization of compound of **3**. (a), (b) Compound **3** in $CHCl_3$. (c), (d) Compound **3** in $CDCl_3$/DMSO (3:1 *V*/*V*).

Isomerization kinetic rate constants

The principle of calculating the rate constant[1]

Based on the ^{1}H NMR data of the photoisomerization of Compound **2** and **3**, we calculated the photoisomerization rate constant (k_{photo}, $E\rightarrow Z$), thermal relaxation rate constant ($k_{thermal}$, $Z\rightarrow E$), and photoisomerization rate constant (k_{photo}, $Z\rightarrow E$).

The following equations were used:

$$\ln\left(\frac{[Z]_\infty-[Z]_0}{[Z]_\infty-[Z]_t}\right)=k\cdot t$$

$[Z]_t$: Molar fraction of the Z configuration at time t (calculated from the integral area ratio of the ^{1}H NMR characteristic peaks);

$[Z]_0$: Molar fraction of the Z configuration at the initial time;

$[Z]_\infty$: Molar fraction of the Z configuration at photostationary state (PSS)/thermal equilibrium

k: First-order rate constant ($k_{thermal}$ for thermal relaxation, k_{photo} for photo-isomerization);

t: Time (converted uniformly to seconds, s)

Data Fitting Method

NMR time-series data were fitted to first-order kinetics using the nonlinear least-squares method. Goodness of fit was evaluated by the coefficient of determination R^2; fits with $R^2>0.98$ were considered reliable. All fittings were performed using OriginPro 9.0.

Table S2 ^{1}H NMR conversion percentage of the E (*trans*-)⇄Z (*cis*-) photoisomerization of compound **2** in CD_2Cl_2.

Conditions	Time (s)	E (*trans*-)/Z (*cis*-)	$[Z]t$ (Z %)
before UV	0 s	100/0	0.00 %
365 nm, 15 mins	900 s	14.0/86.0	86.00 %
365 nm, 30 mins	1800 s	9.9/90.1	90.10 %
365 nm, 60 mins	3600 s	10.2/89.8	89.80 %
dark, 2 hours	7200 s	15.3/84.7	84.70 %
dark, 4 hours	14400 s	16.7/83.3	83.30 %
dark, 16.5 hours	59400 s	22.5/77.5	77.50 %
600 nm, 15 mins	900 s	100/0	0.00 %
600 nm, 30 mins	1800 s	100/0	0.00 %

Note:

The conversion percentage were calculated based on the ^{1}H NMR data of the photoisomerization of compound **2** (Fig. 3a).

365 nm: PSS $[Z]_0$ = 0%, $[Z]_\infty$ = 90.1%; 600 nm $[Z]_0$ = 77.5%, $[Z]_\infty$ = 0%

Table S3 ^{1}H NMR conversion percentage of the E (*trans*-)⇄Z (*cis*-) photoisomerization of compound **2** in DMSO-d_6.

Conditions	Time (s)	E (*trans*-)/Z (*cis*-)	$[Z]t$ (Z %)
before UV	0 s	89.3/10.7	10.70%
365 nm, 15 mins	900 s	13.7/86.3	86.30%
365 nm, 30 mins	1800 s	12.3/87.7	87.70%
365 nm, 60 mins	3600 s	9.0/91.0	91.00%
dark, 2 hours	7200 s	16.0/84.0	84.00%
dark, 4 hours	14400 s	17.4/82.6	82.60%
dark, 21 hours	75600 s	30.6/69.4	69.40%
600 nm, 15 mins	900 s	64.1/35.9	35.90%
600 nm, 30 mins	1800 s	82.6/17.4	17.40%

600 nm, 60 mins	3600 s	92.0/8.0	8.00%

Note:

The conversion percentage were calculated based on the ^{1}H NMR data of the photoisomerization of compound **2** (Fig. 3b).

365 nm: PSS $[Z]_0$ = 10.7%, $[Z]_\infty$ = 87.7%;   600 nm $[Z]_0$ = 69.4%, $[Z]_\infty$ = 8.0%

Table S4 The photoisomerization rate constant (k_{photo}, E→Z) and photoisomerization rate constant (k_{photo}, *Z→E*) of compound **2** in CD_2Cl_2 and DMSO-d_6.

Solvent	Photoisomerization process	Rate constant (s^{-1})	Half-life ($t_{1/2}$)	Fitting (R^2)
CD_2Cl_2	*E→Z* (k_{photo}, 365 nm)	3.3×10^{-3}	3.5 min	0.992
	Z→E (k_{photo}, 600 nm)	$>4.62\times10^{-3}$	<2.5 min	-
DMSO-d_6	*E→Z* (k_{photo}, 365 nm)	2.20×10^{-3}	5.3 min	0.994
	Z→E (k_{photo}, 600 nm)	6.10×10^{-4}	19.0 min	0.996

Table S5 ^{1}H NMR conversion percentage of the *E* (*trans*-)⇄*Z* (*cis*-) photoisomerization of compound **3** in $CDCl_3$.

Conditions	Time (s)	*E* (*trans*-)/*Z* (*cis*-)	[*Z*]*t* (*Z* %)
before UV	0 s	100/0	0.00 %
365 nm, 15 mins	900 s	55.6/44.4	44.40 %
365 nm, 30 mins	1800 s	50.0/50.0	50.00 %
365 nm, 2 hours	7200 s	43.5/56.5	56.50 %
365 nm, 4 hours	14400 s	27.8/72.2	72.20 %
dark, 3 hours	10800 s	83.3/16.7	16.70 %
dark, 6 hours	21600 s	100/0	0.00 %

Note:

The conversion percentage were calculated based on the ^{1}H NMR data of the photoisomerization of compound **3** (Fig. 8a).

365 nm: PSS $[Z]_0$ = 0%, $[Z]_\infty$ = 72.2%;   dark $[Z]_0$ = 72.2%, $[Z]_\infty$ = 0%

Room temperature was approximately 25 ^{o}C

Table S6 ^{1}H NMR conversion percentage of the *E* (*trans*-)⇄*Z* (*cis*-) photoisomerization of compound **3** in $CDCl_3$/DMSO-d_6 (3:1 *V*/*V*).

Conditions	Time (s)	*E* (*trans*-)/*Z* (*cis*-)	[*Z*]*t* (*Z* %)
before UV	0 s	98.0/2.0	2.00%
365 nm, 15 mins	900 s	50.8/49.2	49.20%
365 nm, 30 mins	1800 s	39.7/60.3	60.30%
365 nm, 2 hours	7200 s	33.9/66.1	66.10%
365 nm, 4 hours	14400 s	36.0/64.0	64.00%
dark, 2 hours	7200 s	39.1/60.9	60.90%
dark, 14 hours	50400 s	48.3/51.7	51.70%
600 nm, 15 mins	900 s	73.0/27.0	27.00%
600 nm, 30 mins	1800 s	81.3/18.7	18.70%
600 nm, 60 mins	3600 s	82.0/18.0	18.00%
600 nm, 2 hours	7200 s	100/0	0.00 %

Note:

The conversion percentage were calculated based on the ^{1}H NMR data of the photoisomerization of compound **3** (Fig. 8b).

365 nm: PSS $[Z]_0$ = 2.0%, $[Z]_\infty$ = 66.1%;   600 nm $[Z]_0$ = 51.7%, $[Z]_\infty$ = 0%

Table S8 The photoisomerization rate constant (k_{photo}, E→Z) and photoisomerization rate constant (k_{photo}, *Z→E*) of compound **3** in $CDCl_3$ and $CDCl_3$/DMSO-d_6 (3:1 *V*/*V*).

Solvent	Photoisomerization process	Rate constant (s^{-1})	Half-life ($t_{1/2}$)	Fitting (R^2)
$CDCl_3$	*E*→*Z* (k_{photo}, 365 nm)	3.90×10^{-4}	29.6 min	0.992
	Z→*E* ($k_{thermal}$, dark)	1.55×10^{-5}	12.4 h	0.990
$CDCl_3$/DMSO-d_6 (3:1 *V*/*V*)	*E*→*Z* (k_{photo}, 365 nm)	3.15×10^{-4}	36.7 min	0.991
	Z→*E* (k_{photo}, 600 nm)	1.01×10^{-3}	11.4 min	0.995

Take the calculation of photoisomerization rate constant (k_{photo}, *E*→*Z*, 365nm) in CD_2Cl_2 as an example:

$[Z]_0$=0.00%, $[Z]_\infty$ = 89.8%

t = 900s: ln[(89.80−0)/(89.80-86.00)] ≈ 3.163 k_1 = 3.136/900 = 3.51×10^{-3} s^{-1}

t = 1800s: ln[(89.80−0)/(89.80-90.10)] ≈ 5.699 k_2 = 5.699/1800 = 3.17×10^{-3} s^{-1}

$k_{photo,\,E\to Z}$ was obtained through nonlinear least squares fitting (OriginPro 9.0)

$k_{photo,\,E\to Z}$=3.30×10^{-3} s^{-1}(R^2 = 0.992)

$t_{1/2}$ = ln2/ $k_{photo,\,E\to Z}$ ≈ 3.5 min

References

[1] V. V. Jercaa, F. A. Jercaa, I. Rauc, A. M. Maneac, D. M. Vulugaa, F. Kajzar, *Opt. Mater.*, 2015, 48, 160-164.

Computational details

All calculations were carried out by using the Gaussian 16 suite of computational program[1]. The geometric structures of compound 1 and compound 3 were optimized at M06-2X[2]-D3(0)[3]/def-TZVP[4] level of theory, while the geometric structure of compound 2 was optimized at B3LYP-D3(BJ)[5]/6-311G**[6] level of theory using IEFPCM[7] solvation model in dichloromethane. The obtained relative Gibbs free energies were presented herein. All energies were reported in kcal/mol. The vertical electronic excitation energies of the E, Z isomers and transition states of compound 2 were calculated using time-dependent density functional theory (TD-DFT)[8], and five singlet excited states were considered at B3LYP-D3(BJ)[5]/6-311G**[6] level of theory with IEFPCM[7] solvation model in dichloromethane. The vertical electron excitation energies of the E and Z isomers of compound 3 were also computed at M06-2X[2]-D3(0)[3]/ def-TZVP[4]. The electrostatic potential (ESP)[9] maps and quantitative distribution of electrostatic potential[10-11], UV-Vis absorption spectroscopy, and independent gradient model based on Hirshfeld partition (IGMH)[12] analysis were completed using the open-source program Multiwfn[13]. Visualization was completed using VMD (Visual Molecular Dynamics)[14] and CLYview software[15].

Molecular dynamics (MD) simulations experiment for compound **3** were performed with GROMACS 2023.1 package.[16] The models for compound **3** were generated using the sobtop method. Subsequently, these models were placed in a cubic box with a side length of 10 nm, generated using PACKMOL. The models were solvated with water, and chloride ions and sodium ions were added to maintain charge neutrality. For these simulations, the AMBER14 force field was applied, and the RESP charges were g+enerated as follows: the molecules were optimized at the B3LYP level using the 6-31+G (d, p) basis set with Gaussian 16 program,[17,18] where the solvent effects were corrected by applying the SDM method. The RESP charges were further optimized alongside the wave function using Multiwfn.[13] All calculations were conducted under periodic boundary conditions in all three dimensions. A 1.2 nm cut-off was employed for the neighbor list in the Verlet method and for short-range interactions. Temperature control was achieved using the modified Berendsen thermostat (V-rescale in GROMACS), while pressure control was maintained using the Berendsen pressure bath. Electrostatic interactions were treated using the Particle Mesh Ewald (PME) method.

The computational procedures involved a sequential series of steps. First, an energy minimization phase was performed utilizing the steepest descent algorithm. Following this, the system underwent NVT and NPT equilibration phases, each spanning a duration of 5 ns. Subsequently, in the final production phase, to highlight the distance variation between molecules of compound **3**. This production phase lasted for 5 ns, during which various parameters, including average density, were recorded. Visualization of the simulation results, including graphics and animations, was performed using VMD.[14]

References

[1] M. J. Frisch, G. W. Trucks, H. B. Schlegel, G. E. Scuseria, M. A. Robb, J. R. Cheeseman, G. Scalmani, V. Barone, G. A. Petersson, H. Nakatsuji, X. Li, M. Caricato, A. V. Marenich, J. Bloino, B. G. Janesko, R. Gomperts, B. Mennucci, H. P. Hratchian, J. V. Ortiz, A. F. Izmaylov, J. L. Sonnenberg, D. Williams-Young, F. Ding,

F. Lipparini, F. Egidi, J. Goings, B. Peng, A. Petrone, T. Henderson, D. Ranasinghe, V. G. Zakrzewski, J. Gao, N. Rega, G. Zheng, W. Liang, M. Hada, M. Ehara, K. Toyota, R. Fukuda, J. Hasegawa, M. Ishida, T. Nakajima, Y. Honda, O. Kitao, H. Nakai, T. Vreven, K. Throssell, J. A. Montgomery, Jr., J. E. Peralta, F. Ogliaro, M. J. Bearpark, J. J. Heyd, E. N. Brothers, K. N. Kudin, V. N. Staroverov, T. A. Keith, R. Kobayashi, J. Normand, K. Raghavachari, A. P. Rendell, J. C. Burant, S. S. Iyengar, J. Tomasi, M. Cossi, J. M. Millam, M. Klene, C. Adamo, R. Cammi, J. W. Ochterski, R. L. Martin, K. Morokuma, O. Farkas, J. B. Foresman, D. J. Fox, Gaussian 16, Revision B.01; Gaussian, Inc.: Wallingford CT, 2016.

[2] Zhao, Y.; Truhlar, D. G. J. Chem. Phys. 2006, 125, 194101-1.

[3] (a) Grimme, S., J. Comput. Chem. 2004, 25, 1463-73. (b) Grimme, S.; Antony, J.; Ehrlich, S.; Krieg, H., J. Chem. Phys. 2010, 132, 154104. (c) Ehrlich, S.; Moellmann, J.; Grimme, S., Acc. Chem. Res. 2013, 46, 916-926.

[4] (a) A. Schaefer, H. Horn, and R. Ahlrichs. J. Chem. Phys. 1992, 97, 2571-2577. (b) A. Schaefer, C. Huber, and R. Ahlrichs. J. Chem. Phys. 1994, 100, 5829-5835.

[5] (a) S. H. Vosko, L. Wilk, M. Nusair, Can. J. Phys. 1980, 58, 1200-1211. (b) Grimme, S.; Antony, J.; Ehrlich, S.; Krieg, H. J. Chem. Phys. 2010, 132, 154104.

[6] Tomasi, J.; Persico, M. Chem. Rev. 1994, 94, 2027-2094.

[7] Cancès, E.; Mennucci, B.; Tomasi, J. J. Chem. Phys. 1997, 107, 3032-3041.

[8] F. Furche, R. Ahlrichs. J. Chem. Phys. 2002, 117, 7433-7447.

[9] Zhang, J.; Lu, T. Phys. Chem. Chem. Phys., 2021,23, 20323-20328.

[10] Lu, T.; Sergio, M. J. Phys. Org. Chem., 2013, 26, 473-483.

[11] Struct. Chem., 2014, 25, 1521.

[12] Lu, T.; Chen, Q. X. J. Comput. Chem., 2022, 43, 539-555.

[13] Lu, T.; Chen, F. J. Comput. Chem. 2012, 33, 580-592.

[14] Humphrey, W.; Dalke, A.; Schulten, K., VMD: Visual Molecular Dynamics. Journal of molecular graphics. 1996, 14, 33-38.

[15] CYLview20; Legault, C. Y., Université de Sherbrooke, 2020. (http://www.cylview.org)

[16] Van Der Spoel, D. et al. GROMACS: fast, flexible, and free. J. Comput. Chem. **2005**, *26*, 1701-1718.

[17] Wang, J., Wolf, R. M., Caldwell, J. W., Kollman, P. A. & Case, D. A. Development and testing of a general amber force field. *J. Comput. Chem.* **2004**, *25*, 1157-1174.

[18] Frisch, M. J. et al. Uranyl extraction by N, N-dialkylamide ligands studied by static and dynamic DFT simulations. Gaussian 9, 227 (2009).

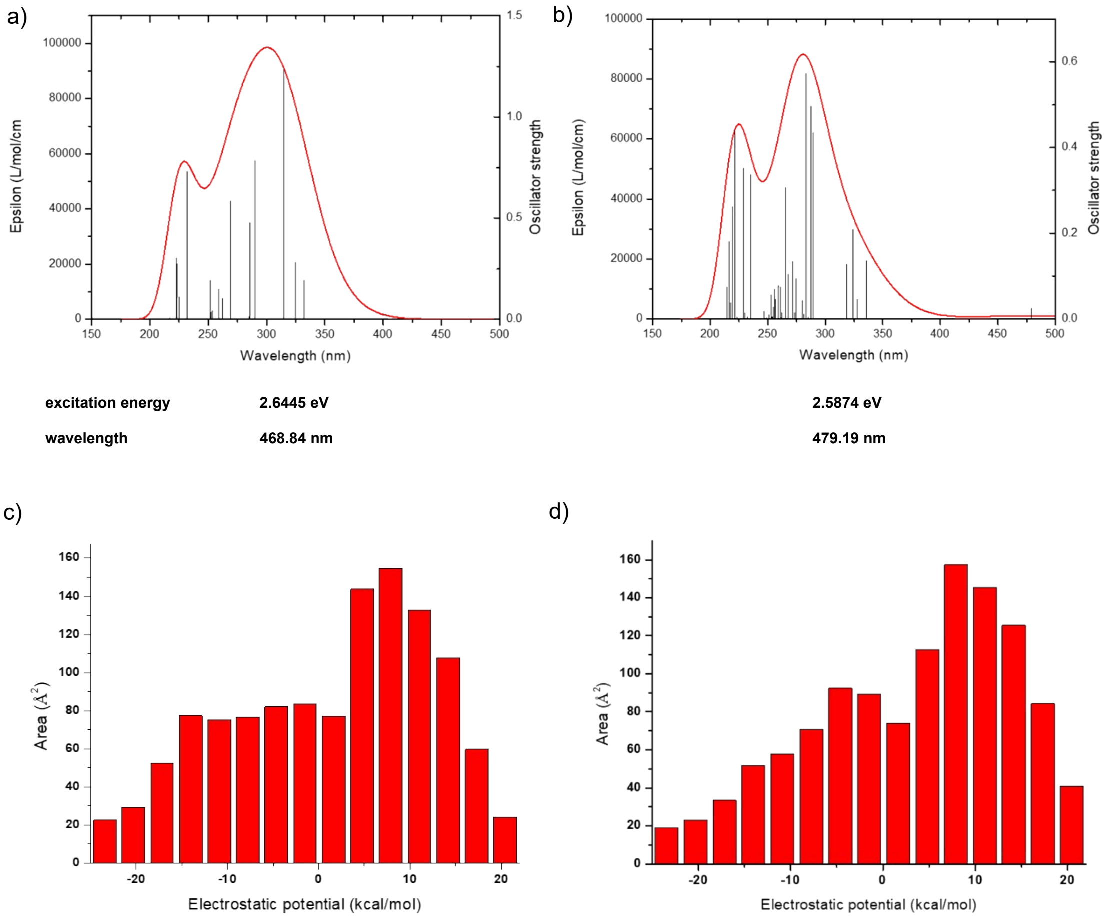

Figure S59. (a), (b) Simulated UV–vis absorption spectra (red line) and oscillator strengths (gray bars), with calculated excitation energies of 2.6445 eV (468.84 nm) for *E*-3 and 2.5874 eV (479.19 nm) for *Z*-3. (c), (d) Electrostatic potential (ESP) distribution histograms, showing the area-weighted population of ESP values across the molecular surface for *E*-**3** and *Z*-**3**, respectively.

After optimizing the structure of the ground state *Z*-**3** and *E*-**3** of compound **3**, TD-DFT calculations were used to obtain the energies corresponding to the lowest 50 excitation states for each compound. Subsequently, the calculated UV-Vis spectra of the two isomers were analyzed using the open-source program Multiwfn. As shown in the figure S58 the lowest excitation energy of the *Z*-isomer was 2.5874 eV, while that of the *E*-isomer was 2.6445 eV.

Table S9 Gibbs free energy and Enthalpy pathway of the *E* and *Z* configurations of compound **2** in DMSO.

	Energy	ΔE(a.u.)	ΔE(kcal/mol)	G	ΔG(a.u.)	ΔG(kcal/mol)
E-DMSO	-5747.042171	0	0.0	-5745.082262	0	0.0
Z-DMSO	-5747.028256	0.0139147	8.7	-5745.06371	0.01855222	11.6

Note that Single point energy was further calculated at M06-2X/def2-SVP level with Gaussian 16. When the solvent effect (dimethyl sulfoxide) was applicable, it was accounted for using the solvation model based in the density (SMD) model. To assess the impact of intramolecular hydrogen bonding (HB) on the isomerization process, we intentionally disrupt the HB and compare the resulting system with one in which the original HB remains intact.

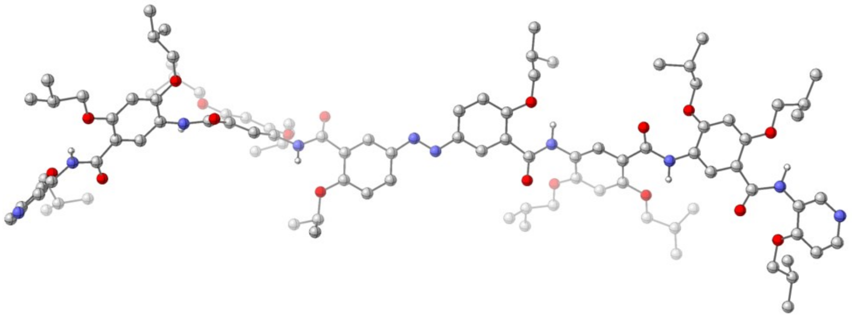

Figure S60. The *E* configuration of compound **2** in DMSO simulated by computer.

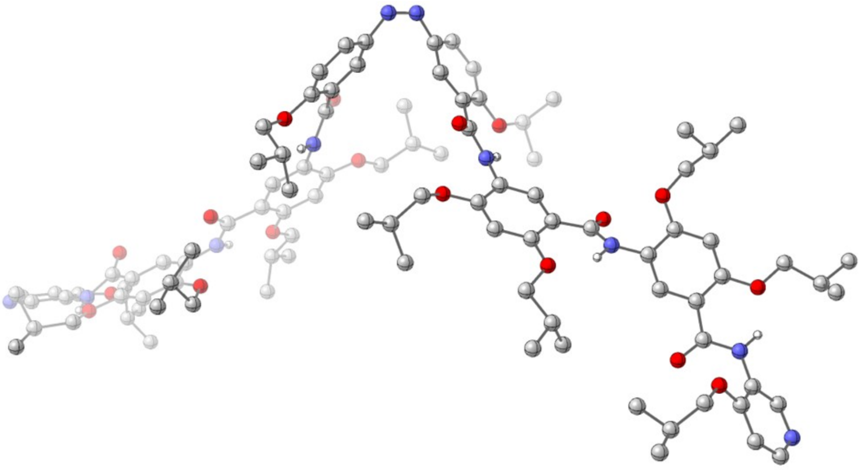

Figure S61. The *Z* configuration of compound **2** in DMSO simulated by computer.

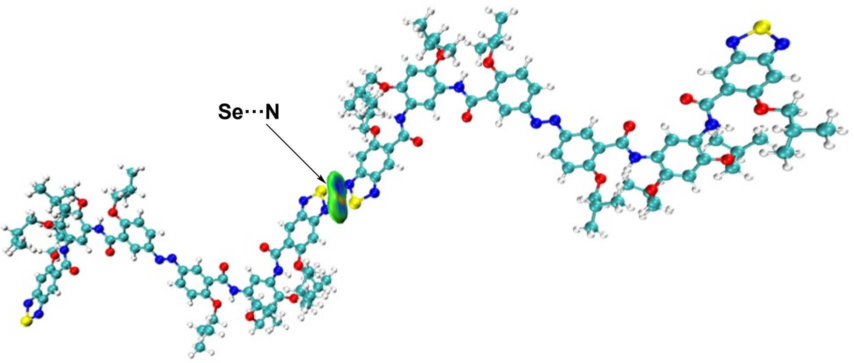

Weak interaction (IGMH) analysis of *E-3* dimer structure

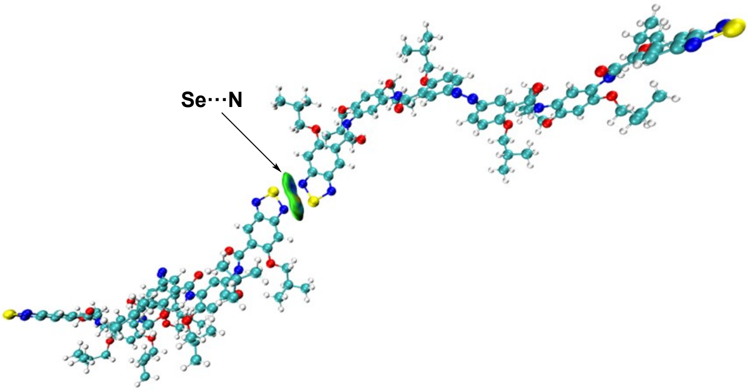

Weak interaction (IGMH) analysis of *Z-3* dimer structure

Figure S62. Weak interaction analysis of *E*-**3** and *Z*-**3** dimers by independent gradient model based on Hirshfeld (IGMH) method.

To confirm the existence of chalcogen bonding, we performed geometric optimization of dimer structures formed by the *Z*-**3** and *E*-**3** conformations of compound **3**. Subsequently, an analysis of the weak interaction (IGMH analysis) between the two conformations was conducted using the open-source program Multiwfn. As shown in Figure S62, clear weak interactions between Se···N exist in both *Z* and *E* states of the helical dimer structure.

The calculated structures

E-1

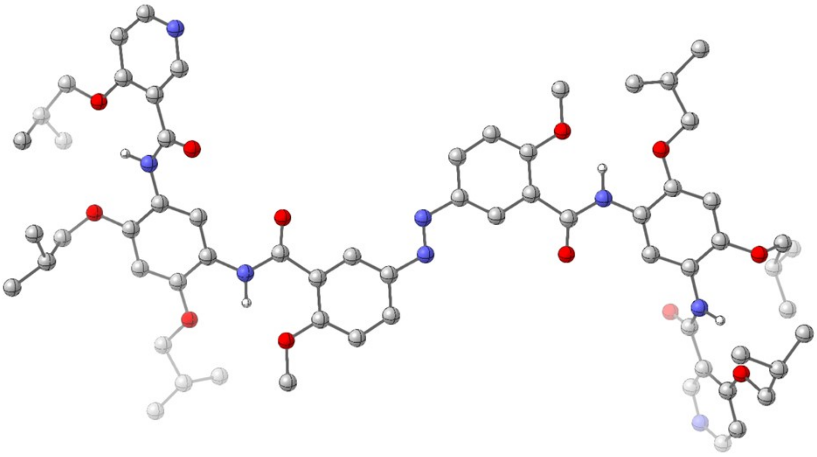

Z-1 (15.8 kcal/mol)

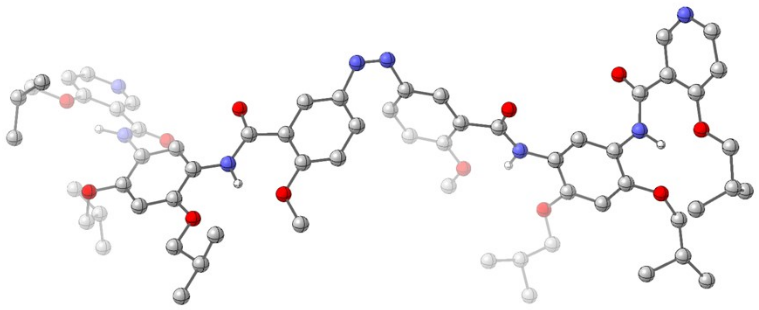

E-2-DCM

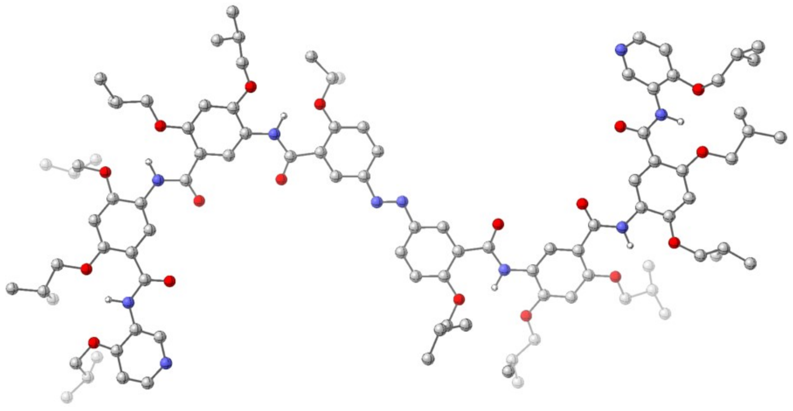

Z-2-DCM (13.9 kcal/mol)

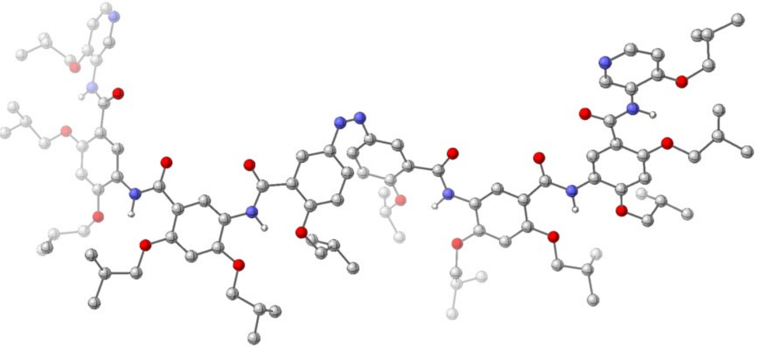

TS-2(TD-DFT)

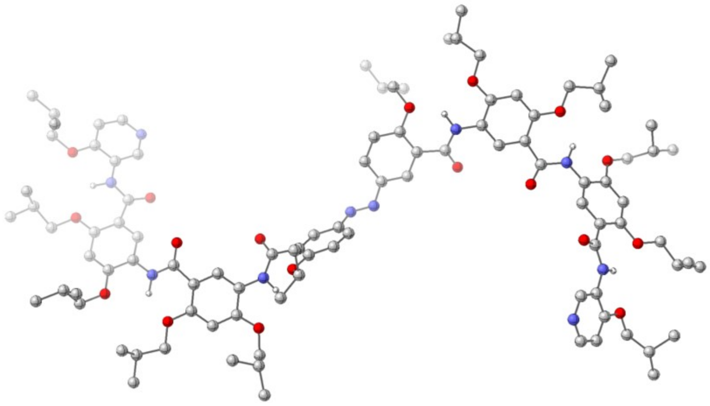

E-3

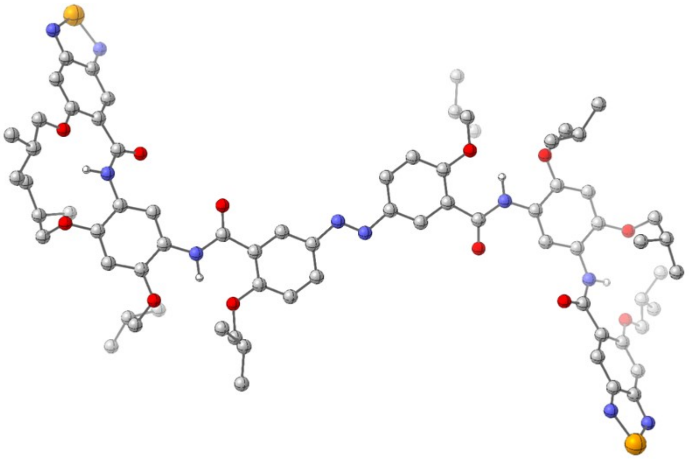

Z-3 (19.5 kcal/mol)

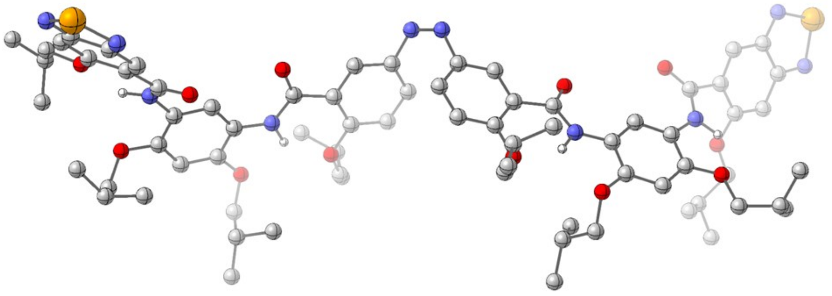

Cartesian coordinates

E-1

O	-11.47834800	3.12814800	-0.47621600
O	-7.92991400	2.25333000	1.57973700
O	-11.18390200	-0.63433800	-0.18158000
O	-7.54261100	-3.84857900	-0.03754800
O	-4.13482800	-4.01308600	0.52950500
O	-4.79454900	0.05419900	-0.14120900
C	-10.11381600	6.40317900	0.38523500
H	-10.34627100	7.45834900	0.28779200
N	-9.47263800	1.35195500	0.18385400
H	-10.36653600	1.51052600	-0.26091000
N	-5.80201000	-1.93617900	0.26627100
H	-5.63903000	-2.92805000	0.37325600
N	0.17576000	-0.54987700	-0.07027700
C	-9.48055400	3.76401000	0.64405000
C	-8.70077700	4.81097200	1.13517800
H	-7.78258300	4.55105100	1.64681600
N	-8.98924400	6.10341800	1.02390300
C	-10.98163200	5.46276200	-0.14605900
H	-11.87411400	5.79657200	-0.65137500
C	-10.67176100	4.11026500	-0.01066600
C	-12.71179900	3.50467900	-1.09021300
H	-12.49721500	4.05616100	-2.01135900
H	-13.26409900	4.15536100	-0.40557600
C	-13.52556300	2.25744000	-1.38091000
H	-14.45343600	2.62505800	-1.83230500
C	-12.82847200	1.34066100	-2.38265800
H	-12.57393700	1.87376000	-3.30062600
H	-13.47843000	0.50470700	-2.64840000
H	-11.91367500	0.92500700	-1.95790700
C	-13.86856700	1.52428200	-0.08707100
H	-12.96369800	1.12446700	0.37434500
H	-14.54802500	0.69309700	-0.28593600
H	-14.35447400	2.18846900	0.63083700
C	-8.96718900	0.03577500	0.16126900
C	-9.87473500	-1.00446800	-0.03431700
C	-9.42606300	-2.31998900	-0.11510700
H	-10.12597500	-3.12711200	-0.25885400
C	-8.07234100	-2.59049800	-0.00144600

C	-7.14801300	-1.55136900	0.17101600
C	-7.60547500	-0.24399500	0.25508000
H	-6.90070100	0.55713600	0.39167400
C	-12.18559400	-1.63973000	-0.17521200
H	-11.95339400	-2.40980200	-0.91832700
H	-13.09105100	-1.12819100	-0.50669200
C	-12.42077900	-2.24789900	1.20450100
H	-11.49638700	-2.72905900	1.53570600
C	-12.78363600	-1.16541600	2.21548300
H	-12.01281400	-0.39475300	2.26069800
H	-12.89899900	-1.59267700	3.21258100
H	-13.72828300	-0.68750900	1.94106800
C	-13.51444700	-3.30646800	1.09732900
H	-14.45130700	-2.85734300	0.75589200
H	-13.70254900	-3.76772600	2.06743100
H	-13.24385300	-4.09747300	0.39422600
C	-8.38585200	-4.92739500	-0.39317900
H	-8.86171200	-4.71969700	-1.36084300
H	-9.17168600	-5.05269400	0.36124500
C	-7.54054000	-6.18532500	-0.48935900
H	-7.08931700	-6.34441500	0.49565300
C	-6.43599500	-6.02097100	-1.52875800
H	-6.87096900	-5.87920100	-2.52184600
H	-5.80338000	-6.91013100	-1.56534700
H	-5.80973000	-5.15565900	-1.30932300
C	-8.43766100	-7.37656400	-0.81299400
H	-9.21882400	-7.50920200	-0.06201500
H	-7.85419800	-8.29671500	-0.85843700
H	-8.91908900	-7.24104900	-1.78498300
C	-4.71658200	-1.13743000	0.08264600
C	-3.35304400	-1.79771400	0.17146400
C	-3.07945300	-3.16583400	0.38486400
C	-1.76381300	-3.61845700	0.44155000
H	-1.54791100	-4.66386100	0.60275700
C	-0.71478300	-2.72687900	0.28785300
H	0.31289600	-3.06478700	0.33019800
C	-0.96829000	-1.38111900	0.07405000
C	-2.28349500	-0.92996600	0.01796300
H	-2.49529600	0.11772300	-0.14596800
C	-3.88862000	-5.37749800	0.81215400
H	-3.31994900	-5.48696300	1.73802700
H	-3.35605500	-5.85798900	-0.01187300
H	-4.86721100	-5.83561200	0.92602900
C	-8.90012800	2.38739700	0.86237200
O	11.20187700	-2.93583500	1.25668600
O	8.76788500	-1.76848900	-1.94249900
O	11.29528500	1.01533000	0.41928200
O	7.57100800	4.04346300	-0.03239500
O	4.22993300	4.11830200	-0.87742400
O	4.90646300	0.06269400	-0.17511400

C	11.11187900	-5.88723600	-0.88445700
H	11.50842000	-6.89552000	-0.93931300
N	9.59273800	-1.12262100	0.06352700
H	10.29142700	-1.32879100	0.76243700
N	5.89995500	2.09101600	-0.38005900
H	5.72628500	3.08155900	-0.48614500
N	-0.07085600	0.64113300	-0.29209300
C	10.08667500	-3.36318100	-0.79458800
C	9.83968300	-4.27262500	-1.82104200
H	9.19750900	-3.94905800	-2.63093100
N	10.32781700	-5.50741000	-1.88682200
C	11.44064600	-5.08337900	0.19573800
H	12.08639300	-5.46928700	0.96935800
C	10.92282800	-3.79147900	0.24888000
C	12.04137500	-3.36478300	2.32879300
H	11.57630700	-4.22601100	2.81907300
H	13.01341800	-3.66405400	1.92387100
C	12.20287800	-2.21044600	3.30300600
H	12.82181700	-2.60818100	4.11450200
C	10.85889200	-1.77917300	3.88568600
H	10.33401600	-2.61839800	4.34612000
H	11.00252300	-1.00931100	4.64453100
H	10.21250000	-1.36334200	3.11043300
C	12.93699000	-1.03524400	2.66124500
H	12.33381600	-0.57126700	1.87831500
H	13.14845400	-0.26734900	3.40673000
H	13.88560100	-1.35196000	2.22165900
C	9.08669200	0.19629400	0.01513000
C	9.95817400	1.25583500	0.20555000
C	9.48067300	2.56570000	0.20820000
H	10.18117100	3.37008300	0.38276800
C	8.13679900	2.80327100	-0.00149000
C	7.24095200	1.73116600	-0.19676600
C	7.72621700	0.43408800	-0.18323600
H	7.05411500	-0.39530200	-0.32817200
C	12.12898800	1.30926200	-0.70921300
H	11.85992800	2.29516100	-1.10307400
H	13.14622200	1.36112700	-0.31335700
C	12.04198400	0.25642700	-1.80685000
H	10.98167900	0.08079500	-2.01941700
C	12.69023700	-1.05013000	-1.35797400
H	12.32063300	-1.36299900	-0.38076100
H	12.49026600	-1.85268600	-2.07059300
H	13.77499500	-0.92608700	-1.28297700
C	12.70746000	0.78822600	-3.07352800
H	13.75349700	1.04492100	-2.88151100
H	12.68964300	0.03461200	-3.86163100
H	12.20094700	1.67991100	-3.44703800
C	8.37580300	5.15703400	0.31116700
H	8.80056900	5.00722400	1.31237600

H	9.19970900	5.25503300	-0.40468600
C	7.50628600	6.40157900	0.29254900
H	7.11677400	6.50546100	-0.72555200
C	6.33968700	6.26811600	1.26653800
H	6.71160000	6.17857800	2.29080400
H	5.69524500	7.14829800	1.22102200
H	5.73895600	5.38453200	1.04855900
C	8.36261500	7.62327200	0.61356100
H	9.19032800	7.73176100	-0.08980900
H	7.76411600	8.53394500	0.57279100
H	8.77983000	7.54511000	1.62080000
C	4.82004600	1.26392900	-0.33713400
C	3.45462700	1.90766800	-0.48119400
C	3.17706200	3.26798000	-0.73575600
C	1.86013500	3.70855400	-0.83740400
H	1.64087000	4.74761300	-1.03165700
C	0.81424600	2.81338600	-0.68445600
H	-0.21458000	3.14243600	-0.75766700
C	1.07165300	1.47468400	-0.43325100
C	2.38802700	1.03468500	-0.33495700
H	2.60228600	-0.00776800	-0.14328300
C	3.98193800	5.47112400	-1.21123800
H	3.43828500	5.54487400	-2.15540500
H	3.42352400	5.97434800	-0.41853500
H	4.96004000	5.93282100	-1.31354300
C	9.42350200	-2.01667000	-0.95034100
Z-1			
O	-12.18279300	-1.47479400	1.50173300
O	-9.02566100	-3.25979900	-0.61870200
O	-10.61815800	1.13394500	-0.82293600
O	-6.10425300	2.02674100	-2.37928700
O	-2.99396500	0.55067100	-2.60122500
O	-5.04345800	-1.94508800	0.04253300
C	-12.53491800	-5.04180600	2.19319400
H	-13.16727700	-5.72831800	2.74609300
N	-9.86283000	-1.26131600	0.04703000
H	-10.67159000	-0.80398000	0.44429500
N	-5.28436000	-0.28164000	-1.48221300
H	-4.77765100	0.34072800	-2.09742100
N	-0.47360600	-3.70977700	-0.02692000
C	-10.90962000	-3.35506100	0.79685800
C	-10.76216900	-4.74186200	0.82863300
H	-9.94117900	-5.16864500	0.26640700
N	-11.54295900	-5.58391800	1.49714700
C	-12.79632600	-3.68218900	2.24568600
H	-13.62435200	-3.32807100	2.83916900
C	-11.97821900	-2.81267600	1.52610900
C	-13.26179800	-0.94174600	2.26940200
H	-13.07365800	-1.13942900	3.32968100
H	-14.19118300	-1.43776000	1.97357500

C	-13.36693500	0.54900300	2.00590100
H	-14.18639000	0.89184200	2.64664900
C	-12.09303500	1.28807900	2.41041300
H	-11.77746100	1.01411000	3.41890400
H	-12.25523400	2.36720600	2.39153600
H	-11.27690900	1.06551900	1.72033300
C	-13.74211300	0.81174800	0.54922300
H	-12.94116100	0.47330000	-0.11044100
H	-13.90333900	1.87719900	0.37506700
H	-14.65880200	0.28506100	0.27645800
C	-8.90210100	-0.44317600	-0.58189400
C	-9.30767500	0.80907200	-1.04347800
C	-8.38896600	1.65741400	-1.65418800
H	-8.69631800	2.62904600	-2.00220400
C	-7.06890400	1.26147200	-1.78846200
C	-6.64409800	0.01802500	-1.30307600
C	-7.56760300	-0.82566700	-0.70320700
H	-7.25008200	-1.78539500	-0.33536800
C	-11.24363900	2.18162300	-1.55603600
H	-12.21015300	1.78499400	-1.87528900
H	-10.67398200	2.41565900	-2.45953200
C	-11.46791400	3.42372900	-0.70257700
H	-12.03128300	3.09560900	0.17456600
C	-10.16819000	4.05522700	-0.21614300
H	-9.54069100	3.32962200	0.30381600
H	-10.37953100	4.87677000	0.47046900
H	-9.59722900	4.46688700	-1.05404500
C	-12.31510800	4.42256600	-1.48571200
H	-11.79310600	4.74607700	-2.39054100
H	-12.51937700	5.31033000	-0.88602800
H	-13.27147600	3.98893900	-1.78530700
C	-6.40629500	3.37725000	-2.67533500
H	-6.78731200	3.87523800	-1.77326500
H	-7.17866300	3.42666100	-3.45181200
C	-5.13581900	4.06143300	-3.14810700
H	-4.79308100	3.52271900	-4.03767400
C	-4.05414700	4.00060600	-2.07390100
H	-4.37407900	4.55374100	-1.18651800
H	-3.12670500	4.45181900	-2.43239600
H	-3.84978200	2.97196500	-1.77489900
C	-5.45082900	5.50188500	-3.54117400
H	-6.20947500	5.54950900	-4.32461700
H	-4.55589100	6.00555000	-3.90805600
H	-5.81728200	6.06355000	-2.67787600
C	-4.56401700	-1.20798300	-0.79676100
C	-3.08685000	-1.30718100	-1.12330500
C	-2.34281900	-0.46834200	-1.97241100
C	-0.97961300	-0.69247000	-2.15701200
H	-0.40315100	-0.06380200	-2.81912500
C	-0.33929100	-1.72229500	-1.48893300

H	0.72110700	-1.88391400	-1.63572300
C	-1.06407600	-2.54992000	-0.64312500
C	-2.42787100	-2.36017600	-0.49887200
H	-3.01043200	-3.02466700	0.12682500
C	-2.28642200	1.34367300	-3.53543800
H	-1.87799100	0.72808200	-4.33972900
H	-1.48276800	1.90056600	-3.04777100
H	-3.01439000	2.04085000	-3.94091500
C	-9.85551100	-2.62496900	-0.00066600
O	12.10928900	-1.89631300	-0.82254200
O	8.56908700	-0.30460300	-2.38637000
O	10.68371800	1.34303000	1.14715900
O	6.23989300	2.02910100	2.88700800
O	2.95660800	1.11227600	2.35170400
O	5.07775000	-1.80749200	0.25782000
C	12.30887900	-2.67623600	-4.37987700
H	12.96858200	-3.15094300	-5.09839700
N	9.72908400	-0.67025700	-0.47807800
H	10.63689300	-0.91219400	-0.10830300
N	5.26200200	0.14381300	1.40145600
H	4.75689100	0.80988000	1.97085900
N	0.54433500	-3.62753600	0.66365700
C	10.61353700	-1.47436200	-2.61621500
C	10.36526400	-1.59615800	-3.98192800
H	9.43686800	-1.18573400	-4.35955100
N	11.17606400	-2.17682400	-4.86073100
C	12.68035600	-2.62191600	-3.04560300
C	11.82123500	-2.00745700	-2.13781300
C	13.32895100	-2.44487600	-0.32225700
H	13.33791000	-3.52214100	-0.51624800
H	14.17077300	-1.97885600	-0.84429600
C	13.40493100	-2.16357300	1.16866500
H	14.33764100	-2.63327300	1.49900000
C	12.24394000	-2.81187100	1.91935700
H	12.18688900	-3.88373200	1.72049300
H	12.35968200	-2.67059100	2.99444500
H	11.29167300	-2.36513800	1.62713300
C	13.48733000	-0.66528000	1.45187000
H	13.66061900	-0.48903200	2.51442200
H	14.30281100	-0.19997100	0.89338700
C	8.84266700	0.03377100	0.36798800
C	9.34367700	1.03784800	1.18027000
C	8.49841900	1.72263900	2.05246300
H	8.92266900	2.48511400	2.69033800
C	7.15409400	1.40962900	2.08727100
C	6.63511600	0.38666600	1.26662800
C	7.48763800	-0.29509900	0.41486600
H	7.10694200	-1.08044200	-0.21670700
C	10.98852300	2.56732800	0.46674300
H	10.30907800	3.35006500	0.82060900

H	12.00436300	2.82612200	0.77512500
C	10.91207400	2.44194400	-1.04952100
H	9.95275800	1.97522200	-1.29896600
C	12.04950900	1.57349800	-1.57937200
H	12.11741800	0.63090600	-1.03491900
H	11.90976000	1.34624700	-2.63799200
H	13.00496700	2.09540100	-1.46820300
C	10.95040200	3.83331900	-1.67614700
H	11.86442800	4.36137200	-1.38866100
H	10.93514800	3.76448700	-2.76445500
H	10.09614700	4.43642000	-1.36346200
C	6.70788600	2.95763900	3.84857800
H	7.44034300	2.46873600	4.50406700
H	7.19852700	3.79842000	3.34540900
C	5.52150700	3.44543300	4.66111600
H	4.83188100	3.93058500	3.96235100
C	4.80959700	2.28124200	5.34309900
H	5.48145100	1.79179800	6.05333900
H	3.93681900	2.63281700	5.89696100
H	4.48478700	1.53417100	4.61813200
C	5.99468300	4.48119400	5.67708400
H	6.49146900	5.32392100	5.19268000
H	5.15265200	4.87047400	6.25035800
H	6.69763200	4.03211900	6.38338400
C	4.57063300	-0.92382900	0.92092400
C	3.09522900	-0.99217300	1.25844000
C	2.32978300	-0.02235600	1.93067800
C	0.97209300	-0.23909900	2.15866000
H	0.38059900	0.49140700	2.69014800
C	0.35854900	-1.39485100	1.70631400
H	-0.69811700	-1.55057400	1.88332600
C	1.10469200	-2.35415900	1.03617700
C	2.46349900	-2.16281400	0.85361900
H	3.06189600	-2.92144800	0.36510000
C	2.19449300	2.12512200	2.97969900
H	1.40041500	2.48116200	2.31968300
H	1.76587600	1.76848900	3.91929300
H	2.89117100	2.93328700	3.18472400
C	9.54452400	-0.76264400	-1.82495400
H	13.62028500	-3.05259400	-2.73661500
H	12.55719600	-0.15917200	1.18554500

E-2 in DCM

N	0.55679800	0.10868200	-0.81640100
N	-0.69617400	0.18393100	-0.75660200
O	3.00248400	-4.85467100	-0.20554600
O	5.19125100	-1.31799300	-0.60877100
N	5.34012100	-3.59283400	-0.53154200
C	9.89406000	-1.72774100	-0.75310200
O	6.24374200	-6.04884500	-0.85176000
O	10.79092700	-4.48362800	-1.31844600

O	-5.32828000	1.63069500	-0.89933800
N	-5.46227100	3.90629300	-0.81138100
C	-9.98068200	2.00833900	-0.38550300
O	-6.35795300	6.35648200	-0.39370200
O	-10.92805800	4.81262400	-0.08262600
N	-11.31110000	2.14773600	-0.09564900
C	2.35773400	-3.66550500	-0.33071700
C	0.95654300	-3.56899500	-0.30751000
C	0.32720700	-2.35000700	-0.46520300
C	1.08919600	-1.18681700	-0.64683900
C	2.47687500	-1.28643500	-0.66756700
C	3.14116700	-2.50410800	-0.51085000
C	-1.22839400	1.47826000	-0.93266400
C	-2.61358800	1.58867900	-0.85696000
C	-3.27569200	2.80773100	-1.00893300
C	-2.49566600	3.96065000	-1.25517400
C	-1.09667000	3.85017900	-1.34081100
C	-0.46911500	2.63099800	-1.17876600
C	4.64743600	-2.41756600	-0.54894900
C	6.72839200	-3.75871200	-0.68158900
C	7.66306500	-2.73367000	-0.65393600
C	9.03468200	-2.95586500	-0.84478100
C	9.46501300	-4.27097300	-1.09274100
C	8.54315800	-5.32137300	-1.10316200
C	7.19167500	-5.07839000	-0.88595700
O	9.37633500	-0.61500700	-0.69158100
C	-4.77965600	2.72981500	-0.91201200
C	-6.84718700	4.07147200	-0.63150100
C	-7.78176700	3.04474400	-0.64268200
C	-9.15103300	3.25974800	-0.43063600
C	-9.58996400	4.58087500	-0.22195900
C	-8.66183600	5.62343100	-0.18737200
C	-7.30789500	5.38473000	-0.38951600
N	11.25056400	-1.91633700	-0.69389600
C	12.22217500	-0.95004100	-0.38097900
C	11.99261700	0.41747300	-0.31670600
C	12.98022300	1.33502800	0.07093100
C	14.25439800	0.83953200	0.39665600
C	14.50886600	-0.53180600	0.33146800
C	13.51895400	-1.42200000	-0.07018400
C	12.53537600	2.76614500	0.10974300
N	13.36938800	3.66533700	0.72950700
O	11.44886400	3.09293900	-0.35905900
O	13.69975100	-2.76429500	-0.17772900
O	15.22081900	1.72776700	0.76173700
C	13.06135100	4.98388700	1.08834400
C	11.93196600	5.69484500	0.66877700
N	11.63665300	6.93635300	1.08305900
C	12.46778000	7.51281700	1.94943700
C	13.63126300	6.91093600	2.42279800

C	13.95285900	5.63490400	1.97379300
O	15.05567900	4.93004600	2.32565200
O	-9.45750600	0.91082400	-0.56568200
C	-12.22469600	1.10928600	0.15605500
C	-11.86991400	-0.19258600	0.47769000
C	-12.81184900	-1.19137800	0.76423800
C	-14.17676700	-0.84782300	0.71616100
C	-14.55214200	0.46552400	0.42664400
C	-13.59738100	1.43504400	0.13737700
C	-12.22102100	-2.50968000	1.16712400
O	-11.00235300	-2.65557600	1.21552400
N	-13.09051700	-3.51368400	1.51154500
O	-13.89230500	2.72817800	-0.15479200
O	-15.10501200	-1.82374700	0.93770000
C	-12.75577700	-4.75277800	2.07419100
C	-11.50411900	-5.08605800	2.60299400
N	-11.22830900	-6.26376700	3.18187600
C	-12.21086300	-7.16097400	3.25598000
C	-13.49260900	-6.94589400	2.75749300
C	-13.77559800	-5.72820000	2.14704600
O	-14.97136200	-5.37386100	1.61746000
C	-6.57195500	7.60843500	0.29345700
C	-6.74970700	8.78413500	-0.66612100
C	-7.95883500	8.63371200	-1.59264500
C	-6.82912200	10.07849700	0.15190200
C	-11.39010900	6.11099000	0.32719000
C	-12.90611000	6.14320200	0.20263500
C	-13.44464500	7.41688300	0.86222300
C	-13.32215400	6.05112700	-1.26772600
C	-15.25679800	3.10457200	-0.41524200
C	-15.78306800	2.58679100	-1.75584200
C	-17.22205100	3.07858500	-1.94398300
C	-14.88020600	3.00976100	-2.91755700
C	-16.49482500	-1.47613500	1.06083400
C	-17.31251700	-2.75896600	1.06683300
C	-18.76322900	-2.43727400	1.44010300
C	-17.22233300	-3.45519900	-0.29343900
C	-16.00517200	-6.36953400	1.48798000
C	-15.73186300	-7.38775300	0.37876900
C	-16.91805700	-8.35462300	0.29629200
C	-15.46059400	-6.70617400	-0.96497300
C	2.33079500	-5.99826700	0.36557800
C	2.05239300	-5.83291000	1.85908000
C	1.38018200	-7.10653700	2.38287800
C	3.33176300	-5.50337300	2.63386000
C	6.58843700	-7.38040100	-1.27128600
C	5.39353100	-8.28635500	-1.01229400
C	5.59616900	-9.62856300	-1.72256600
C	5.17586500	-8.47545800	0.49183400
C	11.24477000	-5.74205800	-1.84277000

C	12.63753700	-5.52977800	-2.41695300
C	13.23091500	-6.87651900	-2.84009200
C	12.60150500	-4.53427600	-3.57982700
C	14.88527200	-3.37589800	0.36677300
C	16.12035000	-3.28823700	-0.53464400
C	17.29039000	-3.97676100	0.17726300
C	15.86281100	-3.89293800	-1.91490300
C	16.59562100	1.31546600	0.83257900
C	17.46055000	2.56549800	0.76919900
C	18.92589600	2.18894500	1.00584000
C	17.26949400	3.29067100	-0.56579200
C	15.89503100	5.40934200	3.39632400
C	16.88217100	6.50458900	2.98584300
C	17.70916200	6.89713200	4.21555500
C	17.77198200	6.06275500	1.82371200
H	-4.91753500	4.76013100	-0.83685500
H	4.79333900	-4.44597900	-0.50593600
H	11.58239500	-2.87235500	-0.73988300
H	-11.67605000	3.09114800	-0.04362200
H	0.35589400	-4.45613900	-0.17036000
H	-0.75166100	-2.28018800	-0.45140900
H	3.07136600	-0.39338000	-0.80115000
H	-0.49480300	4.72538300	-1.53452100
H	0.60748300	2.55315800	-1.24113700
H	7.33507300	-1.72075800	-0.49879100
H	8.88404200	-6.32891500	-1.27167200
H	-7.45207700	2.03112600	-0.78858400
H	-8.99631200	6.63210800	-0.02553400
H	11.01532600	0.79947100	-0.55325700
H	15.48317200	-0.90193700	0.59775400
H	14.24356900	3.30356400	1.09363400
H	11.24348000	5.22436100	-0.01471400
H	12.20758300	8.51167800	2.28642900
H	14.26034700	7.44161600	3.12106000
H	-10.82951800	-0.46569200	0.51014800
H	-15.59380800	0.73333200	0.41674200
H	-14.07898900	-3.33423600	1.37686800
H	-10.70184300	-4.36805100	2.53924500
H	-11.97392500	-8.10689400	3.73352600
H	-14.23821400	-7.72178900	2.84709800
H	-7.42063300	7.51809300	0.97543900
H	-5.67428700	7.75761400	0.89636100
H	-5.85088300	8.82819600	-1.28822100
H	-7.98794100	9.45706000	-2.31049500
H	-7.92190300	7.69872600	-2.15501300
H	-8.89598400	8.65819700	-1.02730900
H	-6.90131300	10.94530900	-0.50874400
H	-5.94661700	10.20892600	0.78403200
H	-7.71193900	10.07758000	0.79940700
H	-11.06195500	6.29194000	1.35568000

H	-10.95964000	6.87915400	-0.32278400
H	-13.29916900	5.27382600	0.74057600
H	-14.53384100	7.45375800	0.78765300
H	-13.17658600	7.46510500	1.92094700
H	-13.04773700	8.31080500	0.37026700
H	-14.40919900	6.08346000	-1.37057600
H	-12.96304800	5.12671900	-1.72066800
H	-12.91105200	6.89371200	-1.83350600
H	-15.89320700	2.78507000	0.41524400
H	-15.23423300	4.19337400	-0.41545900
H	-15.79890900	1.49318500	-1.72154500
H	-17.63681000	2.70121600	-2.88141100
H	-17.87117300	2.74392500	-1.12981400
H	-17.25901500	4.17221400	-1.97859700
H	-15.23538500	2.57281300	-3.85399500
H	-13.85090000	2.68257300	-2.75865100
H	-14.87579500	4.09726200	-3.03243900
H	-16.63235600	-0.90142300	1.98224700
H	-16.79895000	-0.85912700	0.20955600
H	-16.88812400	-3.41542200	1.83393100
H	-19.36378400	-3.34959400	1.45681800
H	-18.83040300	-1.97046900	2.42634700
H	-19.21386500	-1.75653900	0.71054200
H	-17.81631600	-4.37168600	-0.30488400
H	-16.19138200	-3.71375700	-0.53610300
H	-17.60796400	-2.80114600	-1.08244900
H	-16.16125900	-6.86144300	2.45213300
H	-16.89981000	-5.79236700	1.25826600
H	-14.84245000	-7.96413900	0.65073500
H	-16.73995900	-9.11496200	-0.46745200
H	-17.08693000	-8.86672200	1.24771000
H	-17.83694000	-7.82221700	0.03017700
H	-15.20120800	-7.45025900	-1.72196700
H	-14.63525000	-5.99552000	-0.89067700
H	-16.34358700	-6.16373000	-1.31362600
H	1.41946700	-6.21182700	-0.19739300
H	3.02839200	-6.81551500	0.19399800
H	1.35196900	-5.00102100	1.98597500
H	1.14174900	-7.00392800	3.44381600
H	0.45032600	-7.31917500	1.84832600
H	2.04178000	-7.97133500	2.27091100
H	3.10299200	-5.31331300	3.68505100
H	3.82813300	-4.61945000	2.22920000
H	4.03876700	-6.33693800	2.58971300
H	6.85190400	-7.35432000	-2.33342400
H	7.45290800	-7.73765100	-0.70167400
H	4.51658900	-7.79191100	-1.44547700
H	4.74772400	-10.29101700	-1.53675000
H	5.69430100	-9.49938400	-2.80355000
H	6.49696300	-10.13207000	-1.35691000

H	4.25486300	-9.02887400	0.69119000
H	5.11799400	-7.51412900	1.00404900
H	6.00670700	-9.03891500	0.92858400
H	11.25309900	-6.48515600	-1.03884200
H	10.55868900	-6.07640200	-2.62788700
H	13.25404200	-5.11057900	-1.61678800
H	14.24353600	-6.74242500	-3.22725200
H	13.28039700	-7.57585300	-2.00088000
H	12.63140700	-7.33783400	-3.63145300
H	13.60085200	-4.38336200	-3.99308600
H	12.21529800	-3.56649600	-3.25613500
H	11.95836700	-4.90666500	-4.38410000
H	15.08525400	-2.94700800	1.35312600
H	14.60400000	-4.42172400	0.50362000
H	16.38132000	-2.23562500	-0.67572400
H	18.19658000	-3.91588700	-0.42949500
H	17.50109600	-3.51404500	1.14548200
H	17.07351000	-5.03609000	0.34843900
H	16.74464400	-3.78329700	-2.55082500
H	15.02440000	-3.40110500	-2.40896900
H	15.63543800	-4.96046700	-1.83690000
H	16.75798800	0.76223700	1.76325700
H	16.82213700	0.65921700	-0.01421100
H	17.13263500	3.22600900	1.57695100
H	19.55644300	3.08081300	0.99060200
H	19.06360700	1.69476600	1.97150100
H	19.28770300	1.51334100	0.22406600
H	17.89518200	4.18393800	-0.61579400
H	16.23006000	3.59279500	-0.70322100
H	17.54819800	2.63802100	-1.39982100
H	15.26022200	5.73918700	4.22364900
H	16.44158600	4.52180800	3.72019300
H	16.31792200	7.38346900	2.66141100
H	18.41127000	7.69553100	3.96532300
H	17.07366800	7.25086000	5.03209300
H	18.28939500	6.04450900	4.58296100
H	18.44264100	6.87257800	1.52636900
H	17.17487400	5.78043200	0.95609700
H	18.38750100	5.20399900	2.10862300
H	-3.20751400	0.70263900	-0.68085100
O	-3.14192800	5.14462700	-1.38598700
C	-2.47227800	6.31884300	-1.93357400
H	-1.52080200	6.44407400	-1.41200800
C	-3.38261400	7.48456800	-1.60206600
H	-2.94819000	8.41274400	-1.97833500
H	-4.36037700	7.33849900	-2.06289500
H	-3.51670200	7.57269100	-0.52299000
C	-2.25491200	6.14009300	-3.42900400
H	-1.63705900	5.26831100	-3.64840100
H	-3.21713900	6.02007300	-3.93240200

H	-1.75570400	7.02195000	-3.83640000

Z-2 in DCM

N	0.33717100	2.79095000	-1.54281600
N	-0.45727500	2.91225200	-0.58613200
O	2.16219000	-2.39071200	-2.53176800
O	4.82640100	0.75246400	-1.71206300
N	4.66119100	-1.47424400	-2.18736600
C	9.41968100	-0.34367000	-1.60542300
O	5.24391100	-3.97665200	-2.78015000
O	9.98673900	-3.11636400	-2.44792600
O	-4.77960600	0.99926000	0.85130300
N	-4.43403000	-1.11925300	1.62704300
C	-9.27439500	-0.37809400	1.16991300
O	-4.77991200	-3.68865600	2.10527300
O	-9.58995100	-3.25101400	1.87784900
N	-10.55732600	-0.85227700	1.12148300
C	1.71554100	-1.10602500	-2.39585400
C	0.35812000	-0.79761800	-2.53584700
C	-0.10435400	0.47968400	-2.28421200
C	0.79948300	1.47876800	-1.91327800
C	2.15773800	1.21241200	-1.91208600
C	2.64536900	-0.08406000	-2.11876800
C	-0.79204300	1.77216300	0.22948500
C	-2.12947500	1.46406900	0.42418500
C	-2.52307400	0.33658600	1.15113600
C	-1.52195600	-0.45726600	1.75554200
C	-0.17793800	-0.07917500	1.64218400
C	0.18680700	1.01449800	0.87680900
C	4.14179100	-0.22966800	-1.98601100
C	6.01925800	-1.83771000	-2.19978400
C	7.07742900	-0.98726700	-1.91240400
C	8.41630900	-1.39948900	-1.97168800
C	8.68626700	-2.72385800	-2.35853600
C	7.63501300	-3.60056900	-2.64064200
C	6.31467100	-3.17474000	-2.55026100
O	9.06015200	0.82061900	-1.44706700
C	-4.01357200	0.10847800	1.20921800
C	-5.75487800	-1.59793600	1.67139800
C	-6.89077400	-0.82587600	1.47229800
C	-8.18697500	-1.35941200	1.50479100
C	-8.33138300	-2.73349600	1.77605800
C	-7.19809600	-3.52937100	1.95842100
C	-5.92296300	-2.97954500	1.91023800
N	10.71552800	-0.74939700	-1.41867100
C	11.76330200	0.01280700	-0.87301300
C	11.73944600	1.39088100	-0.70903100
C	12.78305800	2.09943600	-0.09614500
C	13.89712900	1.37488400	0.36152500
C	13.94775600	-0.01053400	0.19376300
C	12.90840500	-0.69035800	-0.43216300

C	12.57073500	3.58004600	0.00620800
N	13.42038900	4.28022600	0.82832300
O	11.64611200	4.11879600	-0.59525300
O	12.89745600	-2.03136300	-0.65062400
O	14.91429400	2.06052200	0.95355700
C	13.27310000	5.60956700	1.24522800
C	12.39715800	6.54151000	0.67889300
N	12.23753200	7.79068900	1.14114200
C	12.95228600	8.15167200	2.20545600
C	13.86923000	7.31743200	2.84039400
C	14.05588700	6.03136400	2.34579100
O	14.92423500	5.11535600	2.83980800
O	-8.99394900	0.79150500	0.91667100
C	-11.69095400	-0.15426200	0.66863800
C	-11.65307200	1.01811500	-0.07202100
C	-12.80694800	1.65810500	-0.54780200
C	-14.05565400	1.08019700	-0.24934700
C	-14.11267300	-0.11514100	0.47023100
C	-12.95335700	-0.72493600	0.93836300
C	-12.54499900	2.86433400	-1.39980600
O	-11.39340400	3.22798700	-1.62370200
N	-13.63081500	3.50569400	-1.94010000
O	-12.93722200	-1.88333700	1.64711900
O	-15.18873300	1.72142900	-0.65953000
C	-13.60550100	4.53502900	-2.89093300
C	-12.47997700	4.93040700	-3.62209500
N	-12.49940500	5.88562600	-4.56305500
C	-13.66328100	6.48547000	-4.81094600
C	-14.84562900	6.18404800	-4.14098700
C	-14.82321900	5.20046200	-3.15753300
O	-15.88936500	4.79881700	-2.42400700
C	-4.73358300	-5.11052100	1.85992200
C	-4.58527000	-5.92213200	3.14572900
C	-5.75255600	-5.73300100	4.11754000
C	-4.39555100	-7.39853900	2.77842000
C	-9.77339500	-4.67315000	1.98090500
C	-11.23467600	-4.94663700	2.30341100
C	-11.51014800	-6.44902000	2.18456000
C	-11.57944500	-4.41945200	3.69856500
C	-14.17425100	-2.44136800	2.12640700
C	-14.79270100	-1.65256900	3.28336000
C	-16.07060000	-2.36669300	3.73637000
C	-13.80402300	-1.46951700	4.43761700
C	-16.46533900	1.07207000	-0.53121200
C	-17.55470600	2.08433500	-0.85179700
C	-18.90271300	1.36447200	-0.96189900
C	-17.59007800	3.18532800	0.21110500
C	-17.11705400	5.54980500	-2.49916900
C	-17.04409100	6.90497200	-1.79210500
C	-18.41685700	7.58017800	-1.88287800

C	-16.57490600	6.76498900	-0.34159800
C	1.31077800	-3.46460600	-2.06522900
C	0.96672100	-3.35743700	-0.57859100
C	0.09997000	-4.55775700	-0.18259400
C	2.22504000	-3.25194700	0.28716500
C	5.45328100	-5.26396600	-3.38670100
C	4.11787300	-5.99204800	-3.43593700
C	4.22317500	-7.20847100	-4.36143000
C	3.67078000	-6.39699500	-2.02810100
C	10.32379800	-4.37771200	-3.04686900
C	11.81199300	-4.35757000	-3.36140600
C	12.25383500	-5.74027400	-3.84908500
C	12.13843700	-3.26432200	-4.38260500
C	13.87024500	-2.87475900	-0.00385200
C	15.23442600	-2.92871800	-0.69781600
C	16.13989400	-3.88213700	0.09026600
C	15.11406500	-3.34552100	-2.16367000
C	16.16849700	1.41198400	1.21757500
C	17.20629600	2.49507900	1.47091300
C	18.52006600	1.85305300	1.92554200
C	17.40029200	3.36385800	0.22497000
C	15.57658000	5.36207900	4.10260200
C	16.80048600	6.27723000	4.01709000
C	17.39051100	6.42957700	5.42388300
C	17.83649900	5.75188900	3.02309700
H	-3.71904700	-1.78209800	1.90192300
H	4.00345000	-2.21041000	-2.42289700
H	10.91014500	-1.73493400	-1.54745900
H	-10.70723300	-1.81019100	1.41446900
H	-0.34545100	-1.57218700	-2.80599800
H	-1.16250600	0.69566800	-2.34703400
H	2.86972300	1.98952900	-1.66919700
H	0.59658700	-0.64685500	2.13476600
H	1.23280900	1.27012000	0.77918100
H	6.87837800	0.03565300	-1.64489700
H	7.84908000	-4.61798000	-2.92090000
H	-6.78672800	0.22199200	1.25136900
H	-7.30784900	-4.57940000	2.16156200
H	10.88114100	1.94644400	-1.04316600
H	14.80169200	-0.55680800	0.55391300
H	14.14376600	3.74887700	1.29899100
H	11.79813900	6.24717800	-0.16797100
H	12.80104800	9.16074500	2.57673600
H	14.41814700	7.68047200	3.69578400
H	-10.70322000	1.46879900	-0.30119800
H	-15.06607800	-0.56828100	0.67570400
H	-14.54760100	3.18375300	-1.65156000
H	-11.53251600	4.45611200	-3.42056800
H	-13.66411600	7.25178800	-5.58015200
H	-15.75173300	6.71648600	-4.38871100

H	-5.61398200	-5.41904200	1.29113700
H	-3.85732800	-5.26079500	1.22583500
H	-3.67390400	-5.57304000	3.64005000
H	-5.55662500	-6.26652700	5.05082000
H	-5.90885900	-4.67989100	4.35902800
H	-6.68279300	-6.13209400	3.70057400
H	-4.23196500	-7.99854200	3.67633500
H	-3.53652200	-7.54076700	2.11698600
H	-5.28215400	-7.79230000	2.27095700
H	-9.47089700	-5.13599100	1.03615600
H	-9.14791900	-5.06979700	2.78672400
H	-11.84036400	-4.41671400	1.56048900
H	-12.55791700	-6.66346400	2.40692500
H	-11.29579700	-6.81843200	1.17826700
H	-10.89764400	-7.01671600	2.89254000
H	-12.62274800	-4.62458000	3.94825000
H	-11.41773600	-3.34325800	3.76385700
H	-10.95588400	-4.90762600	4.45478400
H	-14.87658600	-2.54728500	1.29427400
H	-13.90091700	-3.44192900	2.45868400
H	-15.06975100	-0.65966700	2.91680300
H	-16.55331900	-1.81312500	4.54488400
H	-16.78967500	-2.46260500	2.91786500
H	-15.84441300	-3.37148000	4.10776600
H	-14.24318100	-0.84296200	5.21773500
H	-12.88201900	-0.99353500	4.09839000
H	-13.54332900	-2.43246000	4.88516600
H	-16.49307900	0.21553700	-1.21224400
H	-16.59744000	0.71371400	0.49451500
H	-17.31289900	2.53094100	-1.82211200
H	-19.69652600	2.07613700	-1.19994500
H	-18.88784900	0.60080700	-1.74400500
H	-19.16530900	0.87794500	-0.01688700
H	-18.37795300	3.91097900	-0.00256400
H	-16.63985500	3.71762600	0.25907300
H	-17.79423700	2.75603600	1.19756100
H	-17.41429000	5.66122300	-3.54573100
H	-17.84864400	4.90811300	-2.01012600
H	-16.32116600	7.53259500	-2.32198600
H	-18.39003200	8.56719300	-1.41576600
H	-18.73443500	7.70797500	-2.92151100
H	-19.17794600	6.98635200	-1.36633200
H	-16.46480200	7.75010100	0.11817500
H	-15.61228700	6.25343900	-0.28308400
H	-17.29708100	6.19531500	0.24953900
H	0.40839900	-3.51417300	-2.67932300
H	1.89763900	-4.35929600	-2.26164100
H	0.37357700	-2.44963300	-0.42788600
H	-0.18238000	-4.49311100	0.87026200
H	-0.81874100	-4.60092600	-0.77391600

H	0.64123500	-5.49819900	-0.32515500
H	1.95606400	-3.13709700	1.34014100
H	2.83944800	-2.39653900	0.00246100
H	2.83986900	-4.15200800	0.19654800
H	5.86752300	-5.11370300	-4.38862700
H	6.16795900	-5.84293900	-2.79215200
H	3.38840000	-5.29359300	-3.86130200
H	3.27236500	-7.74515700	-4.39660100
H	4.48375000	-6.91423700	-5.38134600
H	4.98601600	-7.90750700	-4.00367900
H	2.66108600	-6.81493600	-2.03888800
H	3.68221700	-5.54208900	-1.35062300
H	4.34436500	-7.15734100	-1.61990000
H	10.07318300	-5.18514600	-2.35104800
H	9.74434600	-4.50906600	-3.96675600
H	12.33411700	-4.13149600	-2.42723900
H	13.32734800	-5.74859300	-4.05093000
H	12.04420300	-6.51478700	-3.10617500
H	11.73965800	-6.01151500	-4.77670600
H	13.20629000	-3.25155700	-4.60997300
H	11.85662100	-2.27932100	-4.00725100
H	11.59790000	-3.44024600	-5.31859100
H	13.97181600	-2.56448900	1.04041600
H	13.41210300	-3.86545800	-0.01934300
H	15.68608400	-1.93317800	-0.67060000
H	17.13349000	-3.92705400	-0.36111900
H	16.25554700	-3.56100600	1.12921000
H	15.72777500	-4.89635200	0.09343500
H	16.09534300	-3.33862700	-2.64439200
H	14.46166000	-2.66653600	-2.71343900
H	14.70538500	-4.35687000	-2.24959000
H	16.05813400	0.75280900	2.08473200
H	16.45571400	0.81062900	0.34856600
H	16.82411400	3.12359300	2.28051700
H	19.26648200	2.62164000	2.13853700
H	18.38344000	1.25520500	2.83087700
H	18.92791900	1.20106800	1.14642200
H	18.14937800	4.13781000	0.40348700
H	16.46723300	3.85049600	-0.06352200
H	17.74179300	2.75406500	-0.61809500
H	14.84032900	5.75272700	4.81085300
H	15.88038900	4.36960500	4.44039300
H	16.47864600	7.26497100	3.67526700
H	18.25579700	7.09587200	5.40546400
H	16.66034100	6.84477400	6.12403500
H	17.72179000	5.46189000	5.81437200
H	18.68363300	6.43875400	2.95522700
H	17.40624300	5.64415200	2.02707400
H	18.21935600	4.77600600	3.33721900
H	-2.90145300	2.07289500	-0.02682400

O	-1.91530100	-1.57366800	2.42294000
C	-0.99412900	-2.30539000	3.27955300
H	-0.06315300	-2.45727200	2.72760400
C	-1.65707000	-3.64751100	3.52104000
H	-1.01997200	-4.26864100	4.15358200
H	-2.61962700	-3.50851600	4.01574400
H	-1.82718700	-4.16657800	2.57708400
C	-0.74369100	-1.52702400	4.56302500
H	-0.31641900	-0.54309900	4.36564700
H	-1.68270400	-1.39477200	5.10546100
H	-0.04816000	-2.07884600	5.19920900

TS-2(TD-DFT) in DCM

N	0.92826700	0.62842300	-1.03285200
N	-0.32480900	0.40183200	-1.09652900
O	3.19877000	5.41671100	0.51586600
O	5.45461400	2.49998600	-1.35266400
N	5.55892400	4.13321400	0.22168700
C	9.86857200	1.86040700	0.36862300
O	6.62844200	6.41737900	1.10643500
O	11.04842100	4.42549200	1.09478300
O	-5.11507200	-0.89524700	-0.39552000
N	-4.92046200	-3.10845700	0.10823900
C	-9.68620900	-2.10888600	0.05797400
O	-5.31190800	-5.67742500	-0.26579300
O	-10.13115600	-5.02985200	-0.26885300
N	-10.93817700	-2.44330800	-0.37629900
C	2.60618700	4.23784600	0.16037500
C	1.24426300	3.99030400	0.35135600
C	0.65361400	2.82910100	-0.07935400
C	1.42411000	1.83643200	-0.72141300
C	2.79978400	2.07163400	-0.87406100
C	3.40607400	3.22924300	-0.42160400
C	-0.91188500	-0.56101100	-0.36077900
C	-2.27099600	-0.83670900	-0.59174100
C	-2.97654500	-1.69865600	0.21898100
C	-2.33302000	-2.32511300	1.31092300
C	-0.99196000	-2.02720800	1.56247000
C	-0.28548600	-1.18477600	0.73693600
C	4.89588200	3.25897800	-0.58615700
C	6.93990100	4.18978400	0.41453900
C	7.76985800	3.09859300	0.23738600
C	9.14053100	3.15829700	0.47526900
C	9.69917600	4.37049300	0.90239700
C	8.86419300	5.46272400	1.12675500
C	7.49955100	5.38915200	0.89378400
O	9.28530300	0.80744300	0.52413800
C	-4.44489800	-1.84285100	-0.04801000
C	-6.23898700	-3.54527500	-0.00971000
C	-7.33480700	-2.70887900	0.06493100
C	-8.64515900	-3.17794500	-0.03202600

C	-8.84915300	-4.55426300	-0.20958200
C	-7.74665000	-5.40259200	-0.29209900
C	-6.45035500	-4.92611800	-0.19125500
N	11.20328400	1.94470500	0.08857200
C	12.05822400	0.85917700	-0.10812900
C	11.85332000	-0.37576000	0.47955400
C	12.68603800	-1.46468100	0.23240700
C	13.76877300	-1.29962600	-0.64011400
C	14.00687200	-0.04919700	-1.20440800
C	13.18102500	1.03236300	-0.94061600
C	12.33798200	-2.71375900	0.97122600
N	12.65595000	-3.88091300	0.33314400
O	11.77845500	-2.66613700	2.04760600
O	13.35828300	2.29077200	-1.44111600
O	14.56789600	-2.37975000	-0.88347100
C	12.22933000	-5.16290000	0.66016500
C	11.43491800	-5.46695000	1.76402500
N	10.97256100	-6.67805800	2.02132800
C	11.26575500	-7.65027000	1.18231300
C	12.06222200	-7.48122300	0.05848500
C	12.58006400	-6.22229100	-0.20659000
O	13.39841500	-5.90088000	-1.25196800
O	-9.40941000	-1.00042800	0.46809700
C	-11.99477800	-1.54570700	-0.53860500
C	-11.80122200	-0.21890700	-0.87458900
C	-12.85340700	0.67870900	-1.05273800
C	-14.16557100	0.21068300	-0.88330000
C	-14.36983900	-1.14092800	-0.61631400
C	-13.31385900	-2.01911600	-0.42646200
C	-12.43545800	2.02929000	-1.53434100
O	-11.33097700	2.19030000	-2.01402900
N	-13.35186900	3.03595400	-1.41416400
O	-13.45291100	-3.35075600	-0.15021300
O	-15.20168300	1.09878400	-0.98157400
C	-13.21124400	4.34975400	-1.85107900
C	-12.09626400	4.84025300	-2.52981700
N	-11.98653200	6.08707300	-2.95347300
C	-12.98260400	6.91763900	-2.72297700
C	-14.13916100	6.55765800	-2.04718600
C	-14.26640700	5.25252100	-1.59428100
O	-15.34701200	4.74817000	-0.92736300
C	-5.34027000	-7.07792000	-0.44836200
C	-5.18482500	-7.85202800	0.87005300
C	-6.37630900	-7.64807400	1.80173700
C	-4.98470800	-9.33473300	0.56784100
C	-10.35745800	-6.39780700	-0.54181900
C	-11.86172800	-6.66446900	-0.45051900
C	-12.19854100	-8.00016200	-1.10721900
C	-12.29893700	-6.64897800	1.01105400
C	-14.72225100	-3.86213600	0.19543200

C	-15.21653100	-3.39573100	1.57355800
C	-16.62644000	-3.92426200	1.82069200
C	-14.25730100	-3.85696800	2.66659800
C	-16.53547500	0.63215800	-0.93319800
C	-17.46701000	1.83621600	-0.76991700
C	-18.88548200	1.46460000	-1.19259100
C	-17.44105200	2.31177400	0.68054000
C	-16.34122900	5.63959100	-0.46656100
C	-15.89236800	6.46668600	0.74667600
C	-16.98833700	7.45622300	1.12985200
C	-15.55157500	5.54609000	1.91526000
C	2.41267900	6.58987200	0.62200300
C	1.72936500	6.98941700	-0.69304000
C	0.88953100	8.24362700	-0.47184800
C	2.76611300	7.20997700	-1.79109000
C	7.10793100	7.60355300	1.70436600
C	5.93431200	8.57515000	1.84473900
C	6.32582500	9.75304000	2.73166500
C	5.49009000	9.05205200	0.46490300
C	11.61449300	5.53665000	1.75755500
C	13.10993600	5.26437600	1.93165500
C	13.80070000	6.48897700	2.52399800
C	13.33021200	4.03289000	2.80548100
C	14.43307100	2.56237600	-2.31431800
C	15.79724200	2.68857500	-1.61724300
C	16.87795400	2.95790500	-2.66035500
C	15.75397500	3.79540000	-0.56918700
C	15.77869000	-2.21005400	-1.58987000
C	16.55562600	-3.52388400	-1.49153100
C	17.74977700	-3.50639700	-2.44044600
C	17.00283900	-3.75913700	-0.05154300
C	13.70050300	-6.88076000	-2.22389300
C	14.75609400	-7.90950300	-1.78814200
C	14.94313800	-8.93994200	-2.89822700
C	16.07434500	-7.21999800	-1.45334800
H	-4.25864300	-3.84673500	0.30053400
H	5.03022500	4.87303300	0.66552000
H	11.58612600	2.84636700	-0.15787300
H	-11.16511300	-3.42294800	-0.48032100
H	0.63112100	4.72607300	0.84902100
H	-0.39724300	2.64439600	0.08560100
H	3.41680000	1.33117200	-1.35955000
H	-0.48254100	-2.46643100	2.40624100
H	0.75133500	-0.95907300	0.93688800
H	7.35652700	2.15709900	-0.08580300
H	9.28849800	6.38446000	1.48630700
H	-7.19169600	-1.64751900	0.19316300
H	-7.90809100	-6.45631900	-0.43625600
H	11.01511500	-0.52098300	1.14064400
H	14.83913000	0.07048800	-1.87631200

H	13.19833500	-3.82053200	-0.51893600
H	11.15554000	-4.69132600	2.45941100
H	10.85088800	-8.62133300	1.41716500
H	12.25581900	-8.32415200	-0.58439900
H	-10.79976600	0.15835300	-1.00568500
H	-15.37560700	-1.51870500	-0.56120900
H	-14.25498900	2.81290700	-1.01546700
H	-11.25812800	4.19338000	-2.73399800
H	-12.85473400	7.92701900	-3.09050900
H	-14.91142800	7.29310200	-1.88975500
H	-6.23825200	-7.40110700	-0.98212700
H	-4.47575700	-7.29359700	-1.08649700
H	-4.28732700	-7.46809700	1.36686200
H	-6.19359900	-8.14290100	2.75291900
H	-6.54375600	-6.59098800	1.99354000
H	-7.27883700	-8.07343100	1.36681100
H	-4.84751000	-9.89130900	1.49134500
H	-4.10962500	-9.48807100	-0.05906900
H	-5.85271200	-9.73842300	0.05031000
H	-9.98693600	-6.64041700	-1.54792400
H	-9.83846900	-7.03016800	0.19083500
H	-12.37528900	-5.85826100	-0.98339900
H	-13.26555300	-8.19543500	-1.03650700
H	-11.92040500	-7.99785600	-2.15854800
H	-11.66921800	-8.81186700	-0.61184000
H	-13.36981500	-6.81975100	1.09096600
H	-12.06072700	-5.68914700	1.45995800
H	-11.78855900	-7.43269700	1.56833100
H	-15.46243000	-3.62430600	-0.57998000
H	-14.58304700	-4.94604900	0.20697600
H	-15.23798200	-2.30126700	1.58329300
H	-16.98571300	-3.59604500	2.79276000
H	-17.31778700	-3.56586700	1.06042400
H	-16.63271900	-5.01209400	1.80272200
H	-14.55162900	-3.44215700	3.62763800
H	-13.24672900	-3.52373700	2.44331700
H	-14.25925000	-4.94184600	2.74370900
H	-16.76567700	0.08680500	-1.85921500
H	-16.68393000	-0.04118700	-0.07971100
H	-17.09299900	2.63618800	-1.41577700
H	-19.55071200	2.31501300	-1.06619000
H	-18.91284200	1.16156600	-2.23656900
H	-19.26070900	0.64359000	-0.58446500
H	-18.10349900	3.16345700	0.81630700
H	-16.43325100	2.60263900	0.96369400
H	-17.77531800	1.51721400	1.34590200
H	-16.67943200	6.29606800	-1.27784700
H	-17.17604800	4.99561700	-0.18114700
H	-14.98960700	7.02259200	0.47243100
H	-16.67614500	8.04872300	1.98595800

H	-17.20762500	8.13302300	0.30679300
H	-17.90254500	6.92784500	1.39296500
H	-15.15322200	6.12196600	2.74694100
H	-14.80422300	4.81548200	1.61483100
H	-16.43968900	5.01690700	2.25482000
H	1.67020500	6.48961600	1.42407400
H	3.13039500	7.36009900	0.91470000
H	1.07009800	6.17197600	-1.00306900
H	0.39153300	8.52719500	-1.39517300
H	0.12988800	8.07463800	0.28812400
H	1.51843000	9.07204000	-0.15219200
H	2.27238000	7.37800400	-2.74495300
H	3.41097900	6.33944900	-1.88845800
H	3.38230900	8.07727700	-1.56507200
H	7.53280800	7.37846800	2.69276300
H	7.88822800	8.06064900	1.08032700
H	5.11194500	8.02730400	2.31653200
H	5.49430800	10.44730200	2.82148400
H	6.60019200	9.41524500	3.72832300
H	7.17209500	10.28602400	2.30298300
H	4.58414200	9.64973100	0.53851600
H	5.30204600	8.19620400	-0.17779100
H	6.26660600	9.66306600	0.00855900
H	11.47589900	6.45002100	1.16123300
H	11.13927800	5.67764500	2.73782300
H	13.51947100	5.07092900	0.93577000
H	14.86651100	6.30309200	2.62727800
H	13.66433700	7.35974400	1.88631800
H	13.39527900	6.71546000	3.50788300
H	14.39060500	3.80199400	2.87448800
H	12.81211800	3.17411000	2.38685000
H	12.95085100	4.20911100	3.80985300
H	14.47895000	1.81079200	-3.11269500
H	14.17376400	3.52390800	-2.76832300
H	16.02371700	1.74871700	-1.10434300
H	17.85107400	3.03736300	-2.18216000
H	16.92082400	2.15538800	-3.39389300
H	16.67590900	3.88971500	-3.18402500
H	16.70969200	3.87082500	-0.05585400
H	14.98357800	3.57700300	0.16544400
H	15.53482000	4.75397800	-1.03525800
H	15.57235900	-1.97197500	-2.64356600
H	16.36910000	-1.39458400	-1.15097200
H	15.87029100	-4.32428500	-1.78396900
H	18.29731800	-4.44273700	-2.36973500
H	17.42723300	-3.37301400	-3.47085500
H	18.42746800	-2.69446900	-2.18423800
H	17.51440300	-4.71424300	0.03754500
H	16.14233400	-3.75923300	0.61262500
H	17.68615200	-2.97378500	0.26634000

H	12.78337600	-7.39219300	-2.54262000
H	14.08596700	-6.30900800	-3.07431400
H	14.40451400	-8.41925900	-0.88618600
H	15.68159300	-9.68065400	-2.60204500
H	14.00926400	-9.45462300	-3.11391000
H	15.28728900	-8.45735800	-3.81068000
H	16.80147900	-7.94751800	-1.10010400
H	15.91953500	-6.48001600	-0.67278800
H	16.48067800	-6.72512500	-2.33328500
H	-2.77824600	-0.35447100	-1.41260200
O	-3.08725300	-3.18203900	2.04237400
C	-2.60884000	-3.75751500	3.25247700
H	-1.58788500	-4.13842400	3.10686400
C	-3.54226000	-4.92975900	3.53925100
H	-3.24142300	-5.43385500	4.45289200
H	-4.56119000	-4.56740100	3.64707200
H	-3.51367500	-5.63872600	2.71591000
C	-2.63491200	-2.73748500	4.38900000
H	-2.00330400	-1.88292500	4.15896400
H	-3.65214000	-2.38211400	4.53479000
H	-2.28321700	-3.19741100	5.30845200

E- **2** in DMSO

N	0.59258400	-0.56506200	-0.80921100
N	-0.53753300	-0.06884700	-1.12423900
O	5.28511900	2.28385500	-1.52944300
O	5.34996100	-1.51005400	-0.08019800
N	6.49777300	-0.11182900	-1.45155500
C	11.22486400	0.52452900	-0.66783400
O	6.83094700	-2.80680700	-1.87132700
O	11.51959300	-2.42141000	-0.77928000
O	-5.27218500	-0.07442600	-2.67526200
N	-6.44740600	-0.78451300	-0.86758800
C	-10.87561900	0.97598900	0.08120600
O	-7.25543300	-1.72107400	-3.29287600
O	-11.62211800	0.27363000	-2.61473300
N	-12.19195400	0.64100700	-0.04549700
C	4.09676500	1.63075400	-1.42267800
C	2.85646900	2.24624100	-1.60415000
C	1.68151300	1.54918600	-1.43951300
C	1.70653000	0.19865200	-1.07018200
C	2.94581000	-0.41442100	-0.88069200
C	4.13773100	0.26643800	-1.06402200
C	-1.64246900	-0.80041500	-0.74810900
C	-2.88004700	-0.42405500	-1.27248300
C	-4.05172000	-1.06142200	-0.90070100
C	-3.99538300	-2.12411100	0.02708600
C	-2.75510700	-2.50306200	0.54625300
C	-1.60069700	-1.85717200	0.17069100
C	5.37885800	-0.53266200	-0.80097200
C	7.75744500	-0.71923700	-1.32755300

C	8.87219500	0.05371600	-1.08086200
C	10.14948300	-0.47902100	-0.91640800
C	10.28662000	-1.87654700	-0.99216200
C	9.17770900	-2.65907400	-1.29527000
C	7.91455700	-2.10893000	-1.48898300
O	10.99090200	1.71934000	-0.69339400
C	-5.30488400	-0.60307700	-1.58293200
C	-7.74915400	-0.54652700	-1.33312900
C	-8.67020400	0.07641700	-0.51343700
C	-9.97961000	0.31936700	-0.91219800
C	-10.35474400	-0.03378500	-2.21806300
C	-9.44119200	-0.68306100	-3.04001700
C	-8.15189800	-0.98289500	-2.61071100
N	12.45622500	0.01411200	-0.39573500
C	13.61358600	0.75169500	-0.11866700
C	14.41387200	0.37196800	0.94035700
C	15.59195800	1.03186100	1.27819400
C	15.94842300	2.15866000	0.52115600
C	15.16601000	2.53211200	-0.56532200
C	14.01977500	1.83040000	-0.92821300
C	16.32153800	0.47571500	2.45370300
N	17.64576300	0.81034200	2.52545500
O	15.76909500	-0.23890700	3.26519300
O	13.29739600	2.05573000	-2.04370500
O	17.07112800	2.85366000	0.87899800
C	18.52014000	0.40849000	3.53856200
C	19.39019500	1.32701600	4.11134800
N	20.23558700	1.04349400	5.08629700
C	20.23915000	-0.19425900	5.54633900
C	19.45281000	-1.21123300	5.03089300
C	18.58214400	-0.93162800	3.97933900
O	17.82855400	-1.82588300	3.31751200
O	-10.45105100	1.69139300	0.96485200
C	-13.23264900	1.16785400	0.73247100
C	-14.17661500	0.33592200	1.29454900
C	-15.22973700	0.80091600	2.08193400
C	-15.30232700	2.18433100	2.32219000
C	-14.37513200	3.03424000	1.72893600
C	-13.35086300	2.55911400	0.91673000
C	-16.15384500	-0.25613400	2.58020000
O	-15.98965700	-1.42709000	2.29145300
N	-17.16984700	0.16564500	3.38599000
O	-12.46313300	3.32117600	0.25309300
O	-16.30015500	2.65414900	3.12816000
C	-18.14707200	-0.66280000	3.94677400
C	-18.47575700	-0.53142200	5.28955000
N	-19.38360300	-1.26383000	5.90950700
C	-20.00731100	-2.18538000	5.19846200
C	-19.79536700	-2.39144600	3.84559100
C	-18.86228200	-1.60298700	3.17351000

O	-18.63600800	-1.60396600	1.84787400
C	-7.36246100	-1.95309200	-4.68271100
C	-7.74081300	-3.40943100	-4.99233300
C	-9.16576600	-3.73567000	-4.55164700
C	-7.56522900	-3.67919300	-6.48416200
C	-11.99429200	0.10922100	-3.96679100
C	-13.33355200	0.81856500	-4.18910200
C	-13.72804100	0.71599400	-5.65986900
C	-14.41883700	0.23032200	-3.29178300
C	-12.54444600	4.72759500	0.28916700
C	-13.64397200	5.27169200	-0.63675200
C	-13.74638200	6.78702300	-0.49549800
C	-13.34499900	4.86844100	-2.07816800
C	-16.44304700	4.03978800	3.37014800
C	-17.65635700	4.22662400	4.28645200
C	-17.73976300	5.67753700	4.75096500
C	-18.93801600	3.81056900	3.56873900
C	-19.26668000	-2.56196300	1.02377000
C	-18.63254400	-3.95672400	1.12026600
C	-19.48499600	-4.96602800	0.35592100
C	-17.20545000	-3.91518800	0.58405000
C	5.34883300	3.64816700	-1.89881800
C	5.19941700	3.86636900	-3.41104600
C	5.22149300	5.36144200	-3.71397800
C	6.31479500	3.14030000	-4.15942300
C	6.89122900	-4.21515300	-1.95662900
C	5.47439400	-4.72319600	-2.23762800
C	5.51213700	-6.17942700	-2.69269600
C	4.60618800	-4.55470900	-0.99450700
C	11.70800700	-3.82218600	-0.78741200
C	13.16543100	-4.09191500	-0.39887100
C	13.38438800	-5.58987900	-0.20870500
C	14.11442300	-3.53209500	-1.45564500
C	13.58413700	3.16155100	-2.87112600
C	13.09317500	4.49636100	-2.29162300
C	13.64495900	5.65639100	-3.11607500
C	11.56868800	4.51236000	-2.25579900
C	17.29800300	4.15204800	0.36484800
C	18.39989300	4.79851500	1.20988900
C	18.84971300	6.10830900	0.57007900
C	17.90633700	5.02903800	2.63618400
C	17.71852900	-3.15154700	3.78821400
C	18.85097300	-4.05531500	3.27747500
C	18.75761700	-5.42929800	3.93386000
C	18.77696900	-4.16582500	1.75698300
H	-6.36468400	-1.07587300	0.09424600
H	6.49951000	0.84091200	-1.78883000
H	12.50206900	-0.97290500	-0.18528400
H	-12.43006500	-0.10713800	-0.67790400
H	2.80283600	3.28984200	-1.87243500

H	0.72644200	2.03294000	-1.57586900
H	2.98427300	-1.45084500	-0.58250000
H	-2.68721400	-3.30895900	1.26050300
H	-0.64585100	-2.14373000	0.58427200
H	8.77506100	1.12627000	-0.99451600
H	9.30108100	-3.72353800	-1.40002000
H	-8.38258600	0.39853700	0.47641200
H	-9.75255100	-0.99722100	-4.02143700
H	14.12786300	-0.46923400	1.55445500
H	15.47817700	3.37353300	-1.16006400
H	17.94293300	1.59686400	1.96389000
H	19.39815200	2.35222000	3.76261700
H	20.92443200	-0.38968400	6.36007000
H	19.54184400	-2.20600800	5.43651500
H	-14.11997300	-0.73080100	1.13387800
H	-14.45716300	4.09406200	1.89944000
H	-17.10225900	1.10220300	3.75895100
H	-17.97236300	0.21109200	5.89628200
H	-20.72872600	-2.78436100	5.73768200
H	-20.36989700	-3.14394000	3.33067200
H	-8.06164400	-1.25896100	-5.15741000
H	-6.36200400	-1.75090400	-5.08147200
H	-7.04993100	-4.04591000	-4.42977500
H	-9.37279300	-4.79250500	-4.70582600
H	-9.30370500	-3.51107700	-3.49652600
H	-9.88692100	-3.16293100	-5.13281500
H	-7.81063800	-4.71351200	-6.71222000
H	-6.53933300	-3.49276900	-6.79147900
H	-8.22062700	-3.03482300	-7.06701700
H	-11.23523400	0.54888900	-4.62696600
H	-12.09506000	-0.96022600	-4.20459900
H	-13.19576700	1.87224000	-3.92645900
H	-14.65962900	1.24701200	-5.83647800
H	-12.96112000	1.14724400	-6.29924500
H	-13.86836200	-0.32512500	-5.94352900
H	-15.36935200	0.72307500	-3.48111700
H	-14.16429200	0.37007400	-2.24498900
H	-14.53808100	-0.83394100	-3.48468600
H	-12.67840900	5.09047800	1.31520500
H	-11.56649700	5.06451600	-0.06546700
H	-14.59841100	4.81909900	-0.34509900
H	-14.51975400	7.17584200	-1.15349900
H	-13.99213400	7.06791200	0.52666900
H	-12.80162100	7.25583700	-0.76108400
H	-14.16673500	5.14884100	-2.73328200
H	-13.19873600	3.79249700	-2.13866600
H	-12.43881100	5.35862900	-2.42723700
H	-15.54157600	4.43381000	3.85847900
H	-16.60366400	4.57832800	2.42662900
H	-17.51394500	3.58032300	5.15899400

H	-18.59680500	5.81109600	5.40556000
H	-16.84346400	5.96383200	5.29634200
H	-17.85261300	6.34379900	3.89815900
H	-19.78350500	3.85526700	4.25040900
H	-18.85405500	2.79531200	3.18972100
H	-19.13457700	4.47704200	2.73116100
H	-20.34226400	-2.60668700	1.24164700
H	-19.13744100	-2.17223800	0.00986700
H	-18.59513500	-4.24648400	2.17565600
H	-19.03611500	-5.95437800	0.41406000
H	-20.49133500	-5.02251200	0.76682600
H	-19.55794800	-4.68336200	-0.69239900
H	-16.71313100	-4.87187600	0.74100200
H	-16.64150200	-3.14199600	1.09923400
H	-17.20830800	-3.69656500	-0.48234600
H	4.61190600	4.23734500	-1.34219600
H	6.34779800	3.96445600	-1.58313700
H	4.23846200	3.45014900	-3.73260500
H	5.10565200	5.53008900	-4.78146100
H	4.41447500	5.87585500	-3.19678000
H	6.16626000	5.79758900	-3.39726200
H	6.21073300	3.29321500	-5.23080600
H	6.27728300	2.07112700	-3.96284600
H	7.28784300	3.51528600	-3.84896000
H	7.57044600	-4.51117900	-2.77063900
H	7.25821900	-4.64128900	-1.01397200
H	5.06466800	-4.10361300	-3.04156100
H	4.50281900	-6.53775600	-2.87740400
H	6.08859700	-6.28909600	-3.60900700
H	5.96237000	-6.80550200	-1.92474100
H	3.56143500	-4.74132200	-1.23006800
H	4.70870600	-3.54401800	-0.60849900
H	4.91663000	-5.25326000	-0.21968700
H	11.03406900	-4.29957900	-0.06370300
H	11.50594100	-4.22989600	-1.78707900
H	13.35453800	-3.58002100	0.55057200
H	14.41618300	-5.78393000	0.07192500
H	12.73650300	-5.98181600	0.57192800
H	13.17427000	-6.12520200	-1.13244100
H	15.14605000	-3.63746600	-1.12955200
H	13.91428100	-2.47822900	-1.63002000
H	13.99217900	-4.06807300	-2.39489300
H	14.65792100	3.20457800	-3.10024100
H	13.04655700	2.94988900	-3.79991600
H	13.45922600	4.58033300	-1.26308800
H	13.28936900	6.60290600	-2.71659800
H	14.73361600	5.66683900	-3.10319800
H	13.31601800	5.57552300	-4.15006500
H	11.20954700	5.40632600	-1.75136600
H	11.20786100	3.63723500	-1.72143700

H	11.16707800	4.49866800	-3.26748600
H	17.61761600	4.09190100	-0.68510200
H	16.38080000	4.75099700	0.42508900
H	19.24646300	4.10370500	1.23465900
H	19.63091100	6.56768400	1.16974100
H	19.24002200	5.93874700	-0.43094200
H	18.01557600	6.80352400	0.50232500
H	18.71740100	5.38692800	3.26536900
H	17.51924000	4.10610800	3.06103800
H	17.11043400	5.77104100	2.64464700
H	17.66618800	-3.17102900	4.88258000
H	16.76124700	-3.50465200	3.39370900
H	19.80940400	-3.59627200	3.54389300
H	19.54995200	-6.07698000	3.56671800
H	18.85162900	-5.35169800	5.01488000
H	17.80032100	-5.89303800	3.70585000
H	19.63057300	-4.71795900	1.37069200
H	18.77047600	-3.17446300	1.31037000
H	17.86646100	-4.68265500	1.46059800
H	-2.93477400	0.37103900	-2.00024800
O	-5.16594900	-2.72103500	0.35705100
C	-5.20922500	-3.93598200	1.10056400
H	-4.35013300	-4.56453700	0.83106300
C	-6.49480800	-4.63818000	0.67283000
H	-6.58413400	-5.59457600	1.17961100
H	-7.35439600	-4.02090600	0.92085300
H	-6.48787000	-4.80203900	-0.40155700
C	-5.20765400	-3.65095500	2.60209700
H	-4.32430600	-3.08751000	2.89301200
H	-6.08753600	-3.06890900	2.86618800
H	-5.22582000	-4.58549300	3.15582300
Z-**2** in DMSO			
N	0.21092700	7.85099000	-2.95018700
N	-0.51856900	8.10992900	-1.99809800
O	3.59538700	3.53989600	-3.77450700
O	3.40284600	5.23075800	-0.10571400
N	3.56054300	3.20145300	-1.12448100
C	7.22298700	0.19492000	-0.31320900
O	2.82122200	3.19302800	1.53467800
O	6.67590300	0.46648900	2.58942600
O	-2.82170000	3.77718100	-2.33900400
N	-3.18879300	2.95512900	-0.25624400
C	-7.28112800	0.53935200	0.57872000
O	-2.38901000	1.14084400	-2.19215700
O	-6.51256500	-1.30674400	-1.59236800
N	-8.30200900	-0.17943200	0.03440300
C	2.67782300	4.53227100	-3.59914900
C	1.95593800	5.11826900	-4.63766000
C	1.11822700	6.18916800	-4.41038000
C	0.93641200	6.68248600	-3.11778300

C	1.64094700	6.08274100	-2.07303400
C	2.50600000	5.02384100	-2.28918600
C	-0.98329200	7.20120000	-1.06171500
C	-1.48877600	5.95358500	-1.42335100
C	-2.13129800	5.12117700	-0.51878100
C	-2.23889000	5.53660700	0.82483300
C	-1.74089800	6.79071800	1.18186000
C	-1.14522500	7.62181500	0.25907100
C	3.21207000	4.51466100	-1.06777700
C	4.29496800	2.50651700	-0.15326400
C	5.35447100	1.72166300	-0.55373900
C	6.15174300	1.00251500	0.33302400
C	5.86483800	1.11482400	1.70440700
C	4.76544100	1.86354500	2.11438300
C	3.95166700	2.54122400	1.21065800
O	7.36410600	0.19527400	-1.52387700
C	-2.73251500	3.88910100	-1.13126100
C	-3.97957900	1.85138900	-0.61242700
C	-5.16671900	1.63584400	0.06069600
C	-6.04128500	0.60142700	-0.25185400
C	-5.67406400	-0.27237100	-1.28989300
C	-4.47141200	-0.07798100	-1.95603700
C	-3.60469000	0.96410700	-1.63632000
N	8.02004100	-0.53203800	0.51503100
C	9.06084500	-1.35585700	0.06204100
C	10.31863400	-1.25461600	0.61880900
C	11.39928700	-2.02615100	0.19670300
C	11.18965300	-2.91330100	-0.87106700
C	9.91602200	-3.05172200	-1.41130000
C	8.83277400	-2.31430400	-0.94526000
C	12.68553700	-1.76494200	0.90588900
N	13.63219500	-2.74273400	0.77403700
O	12.85599100	-0.75669100	1.56102000
O	7.55354800	-2.48636900	-1.33542800
O	12.25568900	-3.62442100	-1.34953900
C	14.91776000	-2.69408500	1.32034300
C	16.01408900	-3.05281200	0.54682400
N	17.26599500	-3.01600600	0.96738800
C	17.47969300	-2.59893100	2.20195200
C	16.47017400	-2.25708700	3.08626900
C	15.14351400	-2.32697900	2.66484900
O	14.05851400	-2.12004300	3.42965800
O	-7.35408300	1.11227900	1.64889300
C	-9.55835100	-0.38688300	0.61740700
C	-10.09898000	-1.65527900	0.63906900
C	-11.34608300	-1.94632600	1.18937400
C	-12.06374700	-0.88610400	1.77000000
C	-11.53875600	0.40073100	1.72880300
C	-10.31130900	0.68330600	1.13740700
C	-11.74983600	-3.37742900	1.09876600

O	-10.98461000	-4.22486400	0.67643100
N	-13.01369900	-3.66888900	1.51943600
O	-9.78773200	1.91349600	0.98411500
O	-13.27048000	-1.15544500	2.35334700
C	-13.59368200	-4.94085900	1.51276900
C	-14.29074800	-5.38544300	2.62867200
N	-14.87225100	-6.56779700	2.72180500
C	-14.77058000	-7.37654000	1.68297300
C	-14.13656500	-7.03700000	0.49943700
C	-13.54756600	-5.77926400	0.37762300
O	-13.00779300	-5.26497700	-0.74198500
C	-2.14349500	0.76694600	-3.53542200
C	-1.06684000	-0.32145300	-3.64031000
C	-1.51892300	-1.62984900	-2.99614600
C	-0.70890100	-0.54102300	-5.10786600
C	-6.06979600	-2.39217500	-2.38507900
C	-7.10493800	-3.51230200	-2.23471700
C	-6.83885700	-4.61687000	-3.25218900
C	-7.08398100	-4.06425300	-0.81128700
C	-10.45112700	3.05008500	1.48847400
C	-11.57976600	3.53473500	0.56439200
C	-12.34986400	4.67087300	1.23038100
C	-10.99543000	3.97567100	-0.77468300
C	-14.04378800	-0.11780000	2.92337900
C	-15.34059500	-0.73963000	3.45026600
C	-16.10626800	0.28329200	4.28418300
C	-16.19911800	-1.24916300	2.29503300
C	-12.86882800	-6.06938400	-1.89428500
C	-11.69666000	-7.05671500	-1.80476100
C	-11.73078200	-8.01158600	-2.99482400
C	-10.37950200	-6.29010200	-1.74401800
C	3.79921800	2.92112700	-5.02904800
C	2.76649000	1.82239800	-5.31606300
C	3.02324900	1.22951100	-6.69800100
C	2.83985500	0.74128800	-4.23933600
C	2.43732300	3.32256200	2.88582700
C	1.19256800	4.21391000	2.92848800
C	0.53932300	4.14079700	4.30591000
C	1.57062600	5.64751500	2.56873900
C	6.48390700	0.61206800	3.98268700
C	7.62250700	-0.13099400	4.68830500
C	7.59040900	0.16883200	6.18409600
C	7.52253800	-1.63277800	4.43235200
C	7.23592300	-3.37404300	-2.38475800
C	7.55122700	-2.80986300	-3.77813800
C	7.39844900	-3.90666700	-4.82875200
C	6.63970400	-1.62366500	-4.07495900
C	12.16822900	-4.28259000	-2.59918400
C	13.59068900	-4.65371000	-3.02917800
C	13.54605200	-5.57840200	-4.24176800

C	14.39640400	-3.39314500	-3.33397400
C	14.19804900	-1.62863900	4.74480600
C	14.46709300	-2.74360600	5.76701200
C	14.73725600	-2.13665400	7.14029900
C	13.27903900	-3.70065400	5.81230900
H	-3.22506900	3.22400600	0.71849900
H	3.59926400	2.79434300	-2.04959300
H	7.99818400	-0.30863800	1.49966000
H	-8.06388900	-0.79543900	-0.73086500
H	2.05561500	4.74943700	-5.64626600
H	0.57298300	6.63807800	-5.22596200
H	1.55800300	6.46737100	-1.06664200
H	-1.82878700	7.13672300	2.20000500
H	-0.77056800	8.58905700	0.55549700
H	5.60413400	1.65098000	-1.60246900
H	4.52191900	1.90685700	3.16249000
H	-5.45971300	2.30107700	0.85977000
H	-4.19236600	-0.76551200	-2.73649000
H	10.50220900	-0.54142300	1.40883200
H	9.76474500	-3.76930500	-2.19952100
H	13.49061700	-3.41102700	0.02869500
H	15.86877400	-3.38462400	-0.47366500
H	18.51590600	-2.55563100	2.50910400
H	16.72517300	-1.96249500	4.09131800
H	-9.54732500	-2.48189100	0.21618600
H	-12.10808700	1.20738800	2.15751600
H	-13.48190800	-2.96348800	2.07170300
H	-14.38034300	-4.74452600	3.49704100
H	-15.23758800	-8.34558400	1.79608100
H	-14.12783800	-7.74291500	-0.31485900
H	-3.06377000	0.44891300	-4.03395800
H	-1.78805400	1.67744200	-4.03220300
H	-0.18567300	0.04160800	-3.10215900
H	-0.70344500	-2.34976400	-2.99528600
H	-1.82584400	-1.46067700	-1.96671300
H	-2.35095100	-2.06334400	-3.54941800
H	0.05937600	-1.30531900	-5.20047200
H	-0.34222800	0.37839000	-5.55750900
H	-1.58317400	-0.86952700	-5.66663300
H	-5.99319800	-2.08463800	-3.43743600
H	-5.08762100	-2.74044300	-2.04152600
H	-8.09053700	-3.07673900	-2.42992600
H	-7.56672400	-5.41406700	-3.13075200
H	-6.90898300	-4.23616600	-4.26892300
H	-5.84449800	-5.03478900	-3.10819000
H	-7.92708200	-4.73007300	-0.64970600
H	-7.14181300	-3.25481400	-0.08771700
H	-6.16360500	-4.61761200	-0.63424400
H	-10.82472800	2.86779500	2.50321300
H	-9.67295500	3.81643800	1.54559900

H	-12.26262500	2.69778700	0.38117500
H	-13.14275000	5.02339500	0.57532900
H	-12.79804500	4.34370400	2.16656200
H	-11.68415300	5.50441200	1.44265500
H	-11.78883000	4.22305000	-1.47606900
H	-10.39034000	3.17790000	-1.19832900
H	-10.36438900	4.85172400	-0.64211400
H	-13.49254800	0.35344500	3.74823100
H	-14.27823200	0.64387600	2.16783500
H	-15.06527900	-1.58623400	4.08794700
H	-17.02685100	-0.15504900	4.65989700
H	-15.51309500	0.61587100	5.13287500
H	-16.36151500	1.15140700	3.67989500
H	-17.07370000	-1.76998100	2.67624400
H	-15.63608200	-1.93630300	1.66857700
H	-16.53464300	-0.41697100	1.67917600
H	-13.80746000	-6.59680100	-2.11054000
H	-12.68274900	-5.35484500	-2.70137700
H	-11.80164100	-7.63345100	-0.87976900
H	-10.89763200	-8.70777600	-2.93976800
H	-12.65535100	-8.58546000	-3.01208100
H	-11.65393500	-7.45700300	-3.92792800
H	-9.55051100	-6.97426800	-1.57670000
H	-10.41425500	-5.57069800	-0.92987300
H	-10.21051100	-5.75651500	-2.67796300
H	3.82453900	3.66091600	-5.83590600
H	4.79387100	2.47153500	-4.94795200
H	1.76648800	2.27073200	-5.29622100
H	2.28424100	0.46464000	-6.92293500
H	2.96790900	1.99590100	-7.46802100
H	4.01073600	0.77521100	-6.73471200
H	2.16517500	-0.07531900	-4.48089600
H	2.55404100	1.14125600	-3.26913000
H	3.85096500	0.34575000	-4.16672400
H	2.21085500	2.33140500	3.30772400
H	3.24319600	3.78277200	3.47284800
H	0.49535500	3.83403400	2.17449400
H	-0.33070000	4.79094300	4.34269600
H	0.21959200	3.12615900	4.53484300
H	1.23813300	4.46361300	5.07527600
H	0.68005200	6.25249100	2.41793200
H	2.15692800	5.65368500	1.65376200
H	2.16508400	6.09372800	3.36390200
H	6.50496200	1.67510400	4.25690300
H	5.51741900	0.18478600	4.28241300
H	8.56548200	0.23676800	4.27044200
H	8.39875600	-0.35458800	6.68762400
H	7.70349500	1.23440700	6.36967900
H	6.64754000	-0.15873700	6.61741500
H	8.38342900	-2.14532000	4.85397400

H	7.48713700	-1.83926400	3.36585200
H	6.62188400	-2.03486600	4.89236700
H	7.73129400	-4.34298600	-2.23441400
H	6.15654600	-3.52640300	-2.29139500
H	8.58578400	-2.45177400	-3.77844400
H	7.61302500	-3.51092800	-5.81834900
H	8.07895800	-4.73438600	-4.63654700
H	6.38119900	-4.29290000	-4.82714100
H	6.90782200	-1.16857000	-5.02578900
H	6.74550400	-0.88149300	-3.28773300
H	5.60117000	-1.94719000	-4.12319300
H	11.55603500	-5.19053400	-2.50464100
H	11.71354100	-3.62118300	-3.34707800
H	14.06040200	-5.18312000	-2.19307500
H	14.55499000	-5.83978900	-4.54925300
H	13.00846100	-6.49570400	-4.01223900
H	13.04969100	-5.08668800	-5.07580300
H	15.43576100	-3.64468800	-3.52946100
H	14.35849100	-2.70232800	-2.49520900
H	13.99293200	-2.89039800	-4.21069100
H	14.97785900	-0.86000300	4.79069300
H	13.23690000	-1.15448500	4.96367600
H	15.35050600	-3.30576800	5.44484000
H	14.91941700	-2.92176600	7.86998200
H	15.60773700	-1.48470100	7.11359900
H	13.88135400	-1.55181900	7.47021600
H	13.49607500	-4.55053000	6.45518300
H	13.05545800	-4.06634800	4.81301500
H	12.39814800	-3.19071500	6.19677200
H	-1.44401600	5.62600700	-2.45238800
O	-2.81728500	4.68777000	1.72080200
C	-3.28096700	5.13104100	2.99482500
H	-2.47821300	5.67309500	3.51339600
C	-3.60594800	3.86252300	3.77719000
H	-3.90427900	4.11888000	4.78936800
H	-4.41786600	3.32058900	3.29854600
H	-2.73246000	3.21692800	3.81865200
C	-4.50909900	6.02519500	2.84477700
H	-4.28288300	6.89640900	2.23388900
H	-5.31218500	5.46801900	2.36771600
H	-4.84452200	6.35862900	3.82274900

E-3

Se	13.36174200	6.09530500	-1.43882700
O	12.45558500	0.89343300	1.74462200
O	11.54031600	-2.68390900	0.57440500
O	7.27015800	-4.87080400	-0.04074600
O	5.43977300	-0.42560700	0.31483100
O	4.04181200	-4.19717000	-0.78840400
N	13.96859400	5.05806000	-0.11277400

N	11.98658000	5.02597700	-1.81824600
N	10.34444100	-0.28786600	0.56590700
H	11.19872800	-0.39817300	1.09336700
N	6.02871100	-2.58493800	-0.06999400
H	5.66138500	-3.50456100	-0.28241300
N	0.60885000	0.09543500	0.05502600
C	13.28369800	2.96032800	0.90149600
H	14.10652700	2.99779200	1.59732200
C	13.15277400	4.03060500	-0.03487500
C	12.05296700	4.01372700	-0.98237100
C	11.12635100	2.92722600	-0.95190200
H	10.29418000	2.90845700	-1.64237900
C	11.26638800	1.91636800	-0.05751000
C	12.38193500	1.94129700	0.88630400
C	13.44503400	0.92658800	2.77010400
H	13.28747400	1.82406400	3.37703000
H	14.44082200	0.98000700	2.31385100
C	10.19249800	0.84344300	-0.17432900
C	9.53752200	-1.43484400	0.41411000
C	8.15381300	-1.36406900	0.28289600
H	7.65751700	-0.40862400	0.28617300
C	7.41452900	-2.52882900	0.11191800
C	8.07447600	-3.76877600	0.09387200
C	9.44626100	-3.83897300	0.25496200
H	9.95190500	-4.79427100	0.27924800
C	10.18111700	-2.66933600	0.39254100
C	12.34793900	-3.35520300	-0.39545300
H	11.81448600	-4.22795600	-0.78303700
H	13.22430600	-3.70404900	0.15459900
C	12.77747100	-2.44861900	-1.54384000
H	13.42080800	-3.07521600	-2.17340900
C	13.60146300	-1.26624300	-1.03995200
H	12.99786800	-0.63033900	-0.38883500
H	13.94873000	-0.65419500	-1.87370600
H	14.47368400	-1.59829200	-0.47218800
C	11.59330900	-1.97679200	-2.38591600
H	10.95793200	-2.81147900	-2.69137000
H	11.94485300	-1.46989100	-3.28578500
H	10.97152900	-1.27043500	-1.83360500
C	7.79192900	-5.99878700	-0.73292300
H	6.98517600	-6.73541100	-0.69949300
H	8.64311900	-6.42105000	-0.18913700
C	8.15809400	-5.70315400	-2.18292300
H	9.01035200	-5.01652000	-2.18961800
C	6.99437400	-5.03628300	-2.90817200
H	7.24967500	-4.84589400	-3.95150000
H	6.72474300	-4.08504000	-2.44746100
H	6.11081700	-5.68154400	-2.88972300
C	8.57520400	-7.00503400	-2.86128200
H	7.73858200	-7.70839700	-2.89079500

H	9.40339900	-7.48670500	-2.33693900
H	8.88993100	-6.82043800	-3.88882400
C	5.13247800	-1.56735100	0.03347300
C	3.67917400	-1.92550000	-0.19972300
C	2.78031000	-0.87859700	-0.01713800
H	3.17196500	0.09355100	0.25281000
C	1.41477200	-1.05448800	-0.16451500
C	0.91364800	-2.30916300	-0.50954400
H	-0.15314400	-2.44251800	-0.62676700
C	1.78453700	-3.36088000	-0.70212900
H	1.39363900	-4.32973000	-0.97904300
C	3.16337000	-3.18330800	-0.55198300
C	3.63669400	-5.55468000	-0.62485400
H	4.52652100	-6.12307200	-0.90356700
H	2.84442500	-5.80279500	-1.33662700
C	3.24087800	-5.89906100	0.80548200
H	2.30954300	-5.37927600	1.04835200
C	2.99498500	-7.40337200	0.88991900
H	3.91674300	-7.95394000	0.68239900
H	2.23584100	-7.73065100	0.17636600
H	2.65927700	-7.68217400	1.88896400
C	4.32091300	-5.45018900	1.78444300
H	4.06385300	-5.75158200	2.80082600
H	4.44657500	-4.36623700	1.77626400
H	5.28594100	-5.89377300	1.52474000
O	9.26349700	1.00997600	-0.93713600
Se	-13.36248300	-6.09459300	1.43962500
O	-12.45522100	-0.89366200	-1.74506500
O	-11.54020400	2.68379100	-0.57449200
O	-7.27011000	4.87076300	0.04082100
O	-5.43960000	0.42565500	-0.31518800
O	-4.04174500	4.19713000	0.78844600
N	-13.96921600	-5.05745300	0.11343500
N	-11.98700500	-5.02554000	1.81868100
N	-10.34430400	0.28779300	-0.56606600
H	-11.19872100	0.39821700	-1.09329100
N	-6.02860100	2.58491700	0.06992300
H	-5.66129800	3.50453900	0.28238600
N	-0.60871000	-0.09539700	-0.05505100
C	-13.28386900	-2.96012400	-0.90136100
H	-14.10676600	-2.99753500	-1.59710900
C	-13.15313500	-4.03023000	0.03523200
C	-12.05321500	-4.01345100	0.98260000
C	-11.12632700	-2.92718800	0.95181800
H	-10.29408900	-2.90848200	1.64221500
C	-11.26621600	-1.91646900	0.05724700
C	-12.38183300	-1.94132900	-0.88648400
C	-13.44483700	-0.92674700	-2.77038500
H	-13.28766000	-1.82441600	-3.37712500
H	-14.44056900	-0.97974700	-2.31395600

C	-10.19214300	-0.84370800	0.17383300
C	-9.53738000	1.43475700	-0.41424400
C	-8.15366800	1.36401400	-0.28304300
H	-7.65734300	0.40858700	-0.28639800
C	-7.41441800	2.52879000	-0.11199600
C	-8.07439400	3.76871300	-0.09385600
C	-9.44618300	3.83888500	-0.25494300
H	-9.95184000	4.79417800	-0.27917300
C	-10.18100800	2.66924500	-0.39260400
C	-12.34787400	3.35506400	0.39533700
H	-11.81427500	4.22757700	0.78325400
H	-13.22404500	3.70426900	-0.15480000
C	-12.77785100	2.44827000	1.54339000
H	-13.42105200	3.07487100	2.17309300
C	-13.60216900	1.26632300	1.03902600
H	-12.99864000	0.63033400	0.38793400
H	-13.94989500	0.65422500	1.87255100
H	-14.47409800	1.59882700	0.47108200
C	-11.59391900	1.97577300	2.38541400
H	-10.95838300	2.81016300	2.69134200
H	-11.94569800	1.46853100	3.28499900
H	-10.97224600	1.26952800	1.83283200
C	-7.79187400	5.99863900	0.73318100
H	-6.98514500	6.73529300	0.69978900
H	-8.64311400	6.42094700	0.18950600
C	-8.15793500	5.70282000	2.18316800
H	-9.01014500	5.01612800	2.18983500
C	-6.99412900	5.03594700	2.90827700
H	-7.24937800	4.84537400	3.95158500
H	-6.72442400	4.08480000	2.44741300
H	-6.11063400	5.68129600	2.88990000
C	-8.57509300	7.00459500	2.86169800
H	-7.73853200	7.70803300	2.89119300
H	-9.40338700	7.48624300	2.33749000
H	-8.88969300	6.81986800	3.88925400
C	-5.13233900	1.56736800	-0.03367100
C	-3.67905000	1.92551300	0.19962900
C	-2.78017200	0.87862800	0.01703300
H	-3.17180500	-0.09350200	-0.25301400
C	-1.41464000	1.05451500	0.16449900
C	-0.91354400	2.30917000	0.50963200
H	0.15324100	2.44252600	0.62691500
C	-1.78444900	3.36087400	0.70222900
H	-1.39356300	4.32970800	0.97921500
C	-3.16327300	3.18330200	0.55201200
C	-3.63662300	5.55466600	0.62516400
H	-4.52645600	6.12301200	0.90395100
H	-2.84437400	5.80265300	1.33700300
C	-3.24076900	5.89928800	-0.80510400
H	-2.30945700	5.37949600	-1.04805400

C	-2.99479400	7.40360000	-0.88927400
H	-3.91651600	7.95418300	-0.68163700
H	-2.23561900	7.73070600	-0.17567500
H	-2.65908800	7.68256500	-1.88827400
C	-4.32081800	5.45064200	-1.78415300
H	-4.06372700	5.75217800	-2.80048500
H	-4.44655300	4.36669800	-1.77614100
H	-5.28581900	5.89425200	-1.52439400
O	-9.26281400	-1.01054200	0.93617300
C	-13.32839200	0.33120300	-3.61092700
H	-12.28140000	0.42651700	-3.91450400
C	-13.73469000	1.56278400	-2.80819800
H	-13.13471500	1.67079700	-1.90525200
H	-13.60779400	2.46936400	-3.40206000
H	-14.78813700	1.49121700	-2.52109500
C	-14.19530400	0.17591400	-4.85803000
H	-15.24281300	0.02710200	-4.58189700
H	-14.14057100	1.07371800	-5.47424800
H	-13.88010600	-0.67296600	-5.46714500
C	13.32884300	-0.33161400	3.61030700
H	12.28185000	-0.42729100	3.91376700
C	14.19559400	-0.17644600	4.85753600
H	13.88018700	0.67224700	5.46680400
H	14.14093800	-1.07439300	5.47355300
H	15.24310700	-0.02740000	4.58154800
C	13.73553700	-1.56286700	2.80727300
H	13.13566600	-1.67074900	1.90424400
H	14.78899400	-1.49095200	2.52029400
H	13.60880400	-2.46965100	3.40085800

Z-3

Se	-13.20198400	6.06686700	-0.82985500
O	-12.35062400	0.02008700	-1.67654300
O	-10.65007100	-2.68404700	0.52912800
O	-6.07224500	-4.23663800	0.72231400
O	-5.10661100	-0.33629800	-1.93873500
O	-2.89701700	-3.14371500	0.21377700
N	-13.94182600	4.54483000	-1.41122700
N	-11.65075500	5.29955500	-0.40157400
N	-9.86861900	-0.47535500	-0.78101700
H	-10.79249100	-0.82669800	-0.98908100
N	-5.23370600	-2.12799200	-0.54865400
H	-4.68425700	-2.84652600	-0.09278700
N	-0.47646900	0.31475300	-3.43790500
C	-13.22985500	2.23064500	-1.59933300
H	-14.18373400	1.94329900	-2.01188000
C	-13.03665800	3.60412700	-1.25955300
C	-11.76505200	4.02426700	-0.69941300
C	-10.73329400	3.05418700	-0.51342900
H	-9.77465600	3.35644800	-0.11449500
C	-10.93212500	1.75287000	-0.84288600

C	-12.22295000	1.33980800	-1.39014300
C	-9.72512500	0.86274500	-0.58106400
C	-8.88748400	-1.42179800	-0.41601300
C	-7.54306000	-1.25440500	-0.73194600
H	-7.21875100	-0.37851300	-1.26807700
C	-6.61291600	-2.20331000	-0.32498400
C	-7.04753300	-3.33333100	0.38755600
C	-8.38879700	-3.51376200	0.67255500
H	-8.73063900	-4.40101800	1.18697200
C	-9.30765000	-2.54574700	0.28890400
C	-11.08094900	-2.89390600	1.87587800
H	-10.35226500	-3.51314600	2.40731700
H	-12.01297700	-3.45634800	1.79144200
C	-11.32149000	-1.59436700	2.63683700
H	-11.69366000	-1.90808300	3.61968700
C	-12.39778300	-0.74693800	1.96272300
H	-12.07306500	-0.44004000	0.96626100
H	-12.59656800	0.15634700	2.54140700
H	-13.33460700	-1.29873500	1.85665100
C	-10.03699900	-0.79334100	2.84350300
H	-9.23826600	-1.41332800	3.25776600
H	-10.21359700	0.03669000	3.52926500
H	-9.67613700	-0.37052400	1.90463300
C	-6.23501700	-4.98561900	1.92042100
H	-5.35744100	-5.63635700	1.95604800
H	-7.11731600	-5.62995200	1.85186100
C	-6.28343700	-4.11788100	3.17283900
H	-7.19430500	-3.51241800	3.13605400
C	-5.08190000	-3.18062700	3.22603400
H	-5.11892900	-2.55809300	4.12109100
H	-5.04564400	-2.52261500	2.35668400
H	-4.15052900	-3.75432800	3.25338600
C	-6.34833100	-5.02302100	4.39972500
H	-5.44532900	-5.63503600	4.47470500
H	-7.20906900	-5.69413000	4.36156200
H	-6.42441400	-4.43057100	5.31201800
C	-4.56422500	-1.22845900	-1.31642800
C	-3.05937400	-1.39385900	-1.38884200
C	-2.42179700	-0.53708700	-2.28199300
H	-3.03192300	0.15273000	-2.85152800
C	-1.04713800	-0.54441500	-2.43293300
C	-0.28052300	-1.47359500	-1.73860400
H	0.79282300	-1.50732700	-1.87630200
C	-0.89596400	-2.35994900	-0.87428700
H	-0.29381400	-3.08113400	-0.34020000
C	-2.27627000	-2.32160100	-0.68173900
C	-2.33491100	-4.41736100	0.52192000
H	-3.03114300	-4.83475800	1.25246200
H	-1.36621200	-4.29518000	1.01523100
C	-2.24198800	-5.34984800	-0.67988900

H	-1.47731300	-4.97089000	-1.36391900
C	-1.81188200	-6.72922400	-0.18670900
H	-2.57422500	-7.15479100	0.47175500
H	-0.87087600	-6.68689000	0.36594100
H	-1.67821100	-7.41262000	-1.02548700
C	-3.57389500	-5.40806100	-1.42074700
H	-3.52531700	-6.13088900	-2.23623900
H	-3.84050300	-4.43928600	-1.84657100
H	-4.38082700	-5.70180400	-0.74395400
O	-8.69065900	1.35962900	-0.18544300
Se	15.16680900	-0.25469600	-4.86050600
O	11.40362900	-1.39488300	-0.11089500
O	10.43732100	1.64037400	2.14176800
O	5.74725800	2.29286300	3.17212200
O	5.03868200	0.71688300	-1.36210900
O	2.68443700	1.92815200	1.85790500
N	15.01121300	-1.09433100	-3.28724000
N	13.53446500	0.46210500	-4.77999100
N	9.86915500	0.86184900	-0.24352500
H	10.72042200	0.73039500	0.28336800
N	5.07934100	1.60285500	0.73041900
H	4.48820400	1.91432800	1.49189200
N	0.48725500	1.04491700	-3.19689500
C	13.29500700	-1.31014500	-1.58401300
H	13.90470300	-1.98380300	-1.00130000
C	13.81789900	-0.80962900	-2.81923300
C	12.99583900	0.05706400	-3.65413000
C	11.68816600	0.43024800	-3.20165300
H	11.07288800	1.08252000	-3.80750900
C	11.24036700	-0.02230500	-2.00980400
C	12.04188400	-0.94084300	-1.21162100
C	12.04350700	-2.34026600	0.73306300
H	12.44916200	-3.15905100	0.13030100
H	12.87291200	-1.85075300	1.26036700
C	9.88510500	0.46437700	-1.54582200
C	8.77904800	1.25379500	0.54337000
C	7.45661000	1.24370800	0.12183400
H	7.21045400	0.96079600	-0.88795900
C	6.44747000	1.58677900	1.02448500
C	6.77865500	1.92934000	2.33669500
C	8.10338800	1.97222900	2.75090700
H	8.33411200	2.27929200	3.76075000
C	9.10324900	1.63479500	1.85658900
C	10.86707900	2.01652400	3.43782000
H	10.42061400	2.97991100	3.70747000
H	10.54083700	1.26072300	4.16361900
C	12.38423000	2.13952000	3.41246500
H	12.67876300	2.42115100	4.42869700
C	13.04901100	0.81029300	3.06294600
H	12.73625400	0.48017800	2.06917100

H	14.13494300	0.91356300	3.05783000
H	12.78613600	0.02978200	3.78039400
C	12.82234100	3.24244500	2.45139100
H	12.35550300	4.19674100	2.70234900
H	13.90454700	3.37409100	2.48484900
H	12.54192700	2.98883300	1.42782500
C	5.62044900	1.61852700	4.42421200
H	6.60956700	1.36372300	4.81960000
H	5.16451000	2.34588100	5.09888800
C	4.75782700	0.36501600	4.34570300
H	3.80207900	0.65315600	3.89596100
C	5.39353100	-0.71953900	3.47994000
H	4.79012100	-1.62855900	3.50898600
H	5.48213700	-0.41221000	2.43862900
H	6.39444800	-0.96649700	3.84657600
C	4.51064900	-0.14528100	5.76408200
H	5.45286700	-0.43401800	6.23832900
H	4.03540400	0.61254900	6.39037300
H	3.86554300	-1.02435100	5.74956900
C	4.45703600	1.18002900	-0.40159000
C	2.95036000	1.34817500	-0.43552600
C	2.36631300	1.11781400	-1.67821400
H	3.01262400	0.85093700	-2.50510200
C	0.99840100	1.21114400	-1.86089700
C	0.18559100	1.61332300	-0.80656200
H	-0.88177600	1.72264700	-0.95064800
C	0.74781000	1.87922600	0.42803600
H	0.11055600	2.19141100	1.24328800
C	2.11989200	1.73737400	0.62847900
C	2.10429200	2.86063700	2.77069600
H	1.11134500	2.52101200	3.07906400
H	2.76192900	2.81090700	3.64172900
C	2.07256900	4.28998200	2.24368400
H	1.33839600	4.35299800	1.43526700
C	3.43894100	4.69259800	1.69691100
H	4.21894500	4.52561700	2.44527400
H	3.70275900	4.11608600	0.80878500
H	3.44337000	5.74817700	1.42204900
C	1.62615000	5.21008000	3.37748400
H	1.52662700	6.23585500	3.02215000
H	0.66376500	4.90353100	3.79291200
H	2.36290700	5.20648200	4.18520100
O	8.94852900	0.55163700	-2.30820400
C	11.01416800	-2.87145500	1.71911900
H	10.30174800	-3.47569800	1.14995900
C	10.25005900	-1.74171200	2.40309800
H	9.67914700	-1.15484800	1.68368800
H	9.55554500	-2.14723700	3.14105100
H	10.93876000	-1.06671600	2.91770600
C	11.72173800	-3.76416300	2.73494600

H	12.41298500	-3.17562700	3.34450500
H	10.99935100	-4.22986000	3.40583400
H	12.29110300	-4.55928600	2.24907200
C	-13.54167200	-0.42402100	-2.32131400
H	-14.40681500	-0.18863100	-1.68998700
C	-13.45326700	-1.92314400	-2.53889700
H	-12.50197500	-2.12600500	-3.04039300
H	-13.64370400	0.10953000	-3.27185600
C	-13.49048100	-2.66925500	-1.20905500
H	-14.44741900	-2.49407800	-0.70830700
H	-13.38338200	-3.74365300	-1.36661100
H	-12.68699700	-2.34894800	-0.54668300
C	-14.59773700	-2.36084000	-3.44960500
H	-14.56393700	-3.43830400	-3.61296000
H	-15.56335500	-2.12503500	-2.99397400
H	-14.55146300	-1.86904000	-4.42271600

CIF for compound **1**

Datablock: exp_2691_autored

Bond precision:	C-C = 0.0026 A		Wavelength=1.54184
Cell:	a=15.5644(2)	b=12.0487(1)	c=16.9585(2)
	alpha=90	beta=95.437(1)	gamma=90
Temperature: 173 K			

	Calculated	Reported
Volume	3165.93(6)	3165.93(6)
Space group	P 21/n	P 1 21/n 1
Hall group	-P 2yn	-P 2yn
Moiety formula	C64 H80 N8 O12	C64 H80 N8 O12
Sum formula	C64 H80 N8 O12	C64 H80 N8 O12
Mr	1153.36	1153.36
Dx, g cm-3	1.210	1.210
Z	2	2
Mu (mm-1)	0.685	0.685
F000	1232.0	1232.0
F000'	1235.79	
h, k, lmax	18, 14, 20	18, 14, 20
Nref	5655	5616
Tmin, Tmax	0.821, 0.860	0.788, 1.000
Tmin'	0.781	

Correction method= # Reported T Limits: Tmin=0.788 Tmax=1.000 AbsCorr = MULTI-SCAN

Data completeness= 0.993 Theta(max)= 67.079

R(reflections)= 0.0493(5024) wR2(reflections)= 0.1227(5616)

S = 1.070 Npar= 386

Alert level C

PLAT220_ALERT_2_C NonSolvent Resd 1 C Ueq(max)/Ueq(min) Range 3.2 Ratio
PLAT230_ALERT_2_C Hirshfeld Test Diff for C29 --C30 . 6.4 s.u.
PLAT242_ALERT_2_C Low 'MainMol' Ueq as Compared to Neighbors of C7 Check
PLAT601_ALERT_2_C Unit-Cell Contains Solvent Accessible VOIDS .LE. 94 Ang**3
PLAT906_ALERT_3_C Large K Value in the Analysis of Variance ...... 7.165 Check
PLAT911_ALERT_3_C Missing FCF Refl Between Thmin & STh/L= 0.597 38 Report

```
  1  1  0,   3  2  0,   6  1  0,  -4  2  1,  -3  0  1,  -2  2  1,
  0  1  1,   0  3  1,   1  3  1,   4  2  1,   5  0  1,  -5  1  2,
 -4  1  2,  -2  1  2,   0  0  2,   0  3  2,   2  0  2,   4  3  2,
 -4  1  3,  -2  2  3,   1  3  3,   2  1  3,   3  3  3,   4  3  3,
 -5  2  4,  -4  0  4,  -4  1  4,  -3  4  4,  -2  1  4,   0  1  4,
    (    8 More Missing: see the .ckf listing file)
```

Alert level G

PLAT003_ALERT_2_G Number of Uiso or U(i,j) Restrained non-H-Atoms 8 Report
PLAT007_ALERT_5_G Number of Unrefined Donor-H Atoms .... 2 Report
H2 H5
PLAT142_ALERT_4_G s.u. on b - Axis Small or Missing .... 0.00010 Ang.
PLAT177_ALERT_4_G The CIF-Embedded .res File Contains DELU Records 2 Report
PLAT178_ALERT_4_G The CIF-Embedded .res File Contains SIMU Records 1 Report
PLAT188_ALERT_3_G A Non-default SIMU Restraint Value has been used 0.0100 Report
PLAT192_ALERT_3_G A Non-default DELU Restraint Value for First Par 0.0010 Report
And 3 other PLAT192 Alerts
More ...
PLAT860_ALERT_3_G Number of Least-Squares Restraints .... 49 Note
PLAT909_ALERT_3_G Percentage of I>2sig(I) Data at Theta(Max) Still 83% Note
PLAT910_ALERT_3_G Missing FCF Reflection(s) Below Theta(Min)[Deg]= 4.05 Note
-1 0 1,
PLAT933_ALERT_2_G Number of HKL-OMIT Records in Embedded .res File 39 Note

```
 -2  2  3,   0  1  4,  -2  1  4,  -2  2  1,   1  2  4,   1  1  0,
  1  3  1,  -4  2  1,   2  0  2,   0  2  4,  -2  1  2,   2  1  3,
  4  0  4,  -1  0  1,  -4  1  2,   1  3  3,  -4  1  3,   5  0  1,
 -4  0  4,  -3  4  5,   3  2  0,  -3  4  4,   4  3  2,   2  3  4,
  3  3  3,   6  1  0,   4  2  1,  -4  1  4,   4  3  3,   4  1  4,
  1  1  6,  -5  2  4,  -5  1  2,   0  1  1,  -3  0  1,   0  0  2,
  0  1  5,   0  3  2,   0  3  1,
```

```
PLAT969_ALERT_5_G The 'Henn et al.' R-Factor-gap value ...........      4.668 Note
              Predicted wR2: Based on SigI**2  2.63 or SHELX Weight 11.47
PLAT978_ALERT_2_G Number C-C Bonds with Positive Residual Density.          8 Info

   0 ALERT level A = Most likely a serious problem - resolve or explain
   0 ALERT level B = A potentially serious problem, consider carefully
   6 ALERT level C = Check. Ensure it is not caused by an omission or oversight
  16 ALERT level G = General information/check it is not something unexpected

   0 ALERT type 1 CIF construction/syntax error, inconsistent or missing data
   7 ALERT type 2 Indicator that the structure model may be wrong or deficient
  10 ALERT type 3 Indicator that the structure quality may be low
   3 ALERT type 4 Improvement, methodology, query or suggestion
   2 ALERT type 5 Informative message, check
```

It is advisable to attempt to resolve as many as possible of the alerts in all categories. Often the minor alerts point to easily fixed oversights, errors and omissions in your CIF or refinement strategy, so attention to these fine details can be worthwhile. It is up to the individual to critically assess their own results and, if necessary, seek expert advice.

Datablock exp_2691_autored - ellipsoid plot

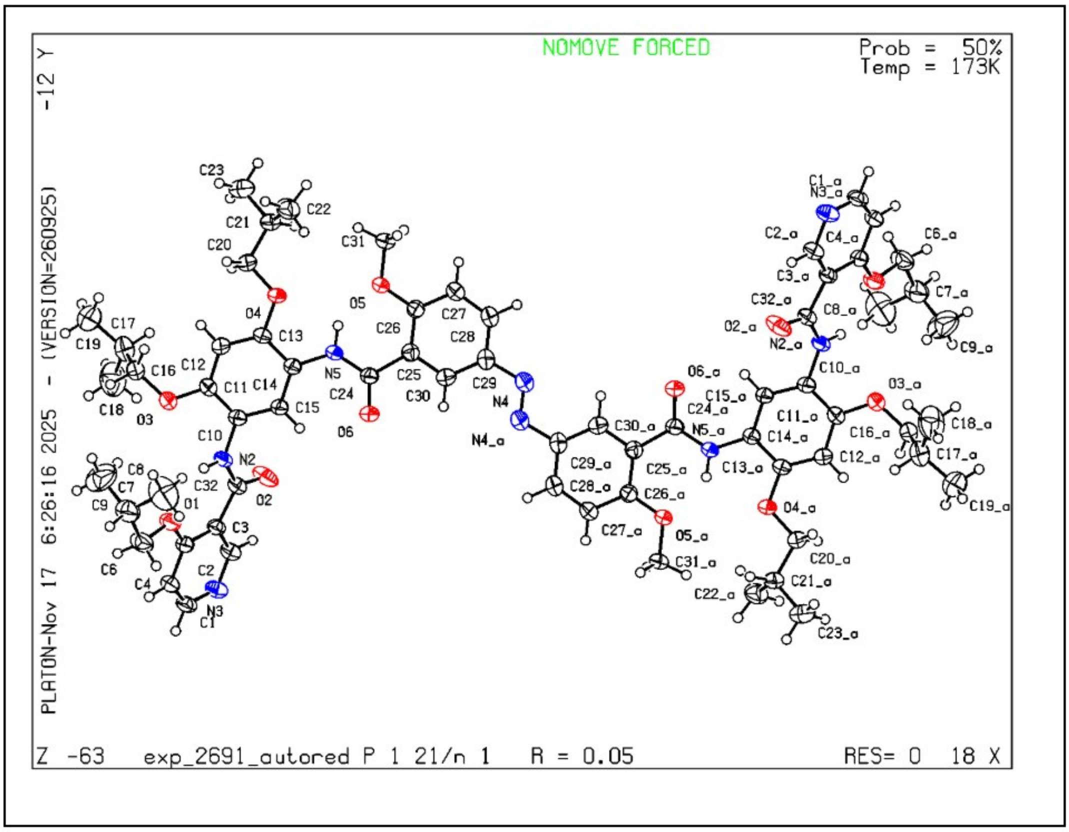

CIF for compound **3**

checkCIF/PLATON report

Structure factors have been supplied for datablock(s) 230414_zwj_128

THIS REPORT IS FOR GUIDANCE ONLY. IF USED AS PART OF A REVIEW PROCEDURE FOR PUBLICATION, IT SHOULD NOT REPLACE THE EXPERTISE OF AN EXPERIENCED CRYSTALLOGRAPHIC REFEREE.

No syntax errors found. CIF dictionary Interpreting this report

Datablock: 230414_zwj_128

Bond precision:	C-C = 0.0034 A		Wavelength=1.34139
Cell:	a=12.311(2)	b=13.452(2)	c=14.199(2)
	alpha=94.632(5)	beta=113.655(5)	gamma=111.398(5)
Temperature:	170 K		

	Calculated	Reported
Volume	1933.0(5)	1933.0(6)
Space group	P -1	P -1
Hall group	-P 1	-P 1
Moiety formula	C72 H90 N10 O12 Se2 [+ solvent]	C72 H90 N10 O12 Se2
Sum formula	C72 H90 N10 O12 Se2 [+ solvent]	C72 H90 N10 O12 Se2
Mr	1445.46	1445.45
Dx,g cm-3	1.242	1.242
Z	1	1
Mu (mm-1)	1.114	1.114
F000	756.0	756.0
F000’	755.39	
h,k,lmax	16,17,18	15,17,18
Nref	8902	8620
Tmin,Tmax	0.887,0.956	0.659,0.752
Tmin’	0.800	

Correction method= # Reported T Limits: Tmin=0.659 Tmax=0.752
AbsCorr = MULTI-SCAN

Data completeness= 0.968 Theta(max)= 60.747

```
R(reflections)= 0.0486( 7347)                          wR2(reflections)=
                                                       0.1381( 8620)
S = 1.069                  Npar= 481
```

```
The following ALERTS were generated. Each ALERT has the format
       test-name_ALERT_alert-type_alert-level.
Click on the hyperlinks for more details of the test.
```

Alert level A

```
PLAT430_ALERT_2_A Short Inter D...A Contact  Se1      ..N1       .       2.87 Ang.
                                                3-x,3-y,1-z  =      2_886 Check
```

Alert level B

```
PLAT430_ALERT_2_B Short Inter D...A Contact  N1       ..N1       .       2.84 Ang.
                                                3-x,3-y,1-z  =      2_886 Check
```

Alert level C

```
PLAT029_ALERT_3_C _diffrn_measured_fraction_theta_full value Low .      0.968 Why?
PLAT094_ALERT_2_C Ratio of Maximum / Minimum Residual Density ....       2.62 Report
PLAT213_ALERT_2_C Atom O2A             has ADP max/min Ratio .....        3.7 prolat
PLAT220_ALERT_2_C NonSolvent    Resd 1  C   Ueq(max)/Ueq(min) Range       3.7 Ratio
PLAT222_ALERT_3_C NonSolvent Resd 1  H   Uiso(max)/Uiso(min) Range        4.5 Ratio
PLAT242_ALERT_2_C Low    'MainMol' Ueq as Compared to Neighbors of        C34 Check
PLAT250_ALERT_2_C Large U3/U1 Ratio for Average U(i,j) Tensor ....        2.2 Note
PLAT911_ALERT_3_C Missing FCF Refl Between Thmin & STh/L=    0.600        226 Report
```

Alert level G

```
ABSMU01_ALERT_1_G  Calculation of _exptl_absorpt_correction_mu
                not performed for this radiation type.
PLAT002_ALERT_2_G Number of Distance or Angle Restraints on AtSite         10 Note
PLAT003_ALERT_2_G Number of Uiso or Uij Restrained non-H Atoms ...         10 Report
PLAT007_ALERT_5_G Number of Unrefined Donor-H Atoms .............           2 Report
PLAT154_ALERT_1_G The s.u.'s on the Cell Angles are Equal ..(Note)      0.005 Degree
PLAT176_ALERT_4_G The CIF-Embedded .res File Contains SADI Records          7 Report
PLAT178_ALERT_4_G The CIF-Embedded .res File Contains SIMU Records          2 Report
PLAT188_ALERT_3_G A Non-default SIMU Restraint Value has been used     0.0200 Report
PLAT188_ALERT_3_G A Non-default SIMU Restraint Value has been used     0.0050 Report
PLAT191_ALERT_3_G A Non-default SADI Restraint Value has been used     0.0400 Report
PLAT191_ALERT_3_G A Non-default SADI Restraint Value has been used     0.0400 Report
PLAT191_ALERT_3_G A Non-default SADI Restraint Value has been used     0.0400 Report
PLAT301_ALERT_3_G Main Residue  Disorder .............(Resd  1  )          8% Note
PLAT410_ALERT_2_G Short Intra H...H Contact  H1       ..H7BD     .       1.99 Ang.
                                                      x,y,z  =      1_555 Check
PLAT414_ALERT_2_G Short Intra D-H..H-X       H3       ..H9AC     .       2.13 Ang.
                                                      x,y,z  =      1_555 Check
PLAT605_ALERT_4_G Largest Solvent Accessible VOID in the Structure        192 A**3
PLAT720_ALERT_4_G Number of Unusual/Non-Standard Labels .........           7 Note
PLAT860_ALERT_3_G Number of Least-Squares Restraints ............         103 Note
PLAT910_ALERT_3_G Missing # of FCF Reflection(s) Below Theta(Min).          1 Note
PLAT912_ALERT_4_G Missing # of FCF Reflections Above STh/L=  0.600         53 Note
```

The short Se···N (2.87 Å) contact is significantly shorter than the sum of the van der Waals radii of Se and N (Σ*r*_vdW ≈ 3.45 Å), which is a typical geometric signature of chalcogen bonding (ChB). The observed distance falls well within the range of known Se···N chalcogen bonds reported in the literature, consistent with the σ-hole-driven donor–acceptor interaction between the selenium center and the nitrogen lone pair[1]. The short N···N contact (2.84 Å) is slightly below the sum of van der Waals radii (Σ*r*_vdW ≈ 3.10 Å) and arises from weak dipole–dipole or lone-pair–π-type electrostatic interactions that contribute to the overall crystal packing stabilization. Such close N···N contacts are frequently observed in crystal structures of nitrogen-containing heterocycles and do not indicate any refinement issues[2]. Both contacts are geometrically reasonable and consistent with the supramolecular assembly driven by chalcogen bonding. Therefore, these alerts are structurally justified and do not reflect any errors in the crystallographic model.

References

[1] C. Z. Liu, C. Zhang, C. G. Li, H. B. Chen, W. Yang, Z.Y. Li, Z. Y. Hu, L. Xu, B. Zhai, Z. T. Li, *Chem. Eur. J.*, **2024**, *30*, e202401150.

[2] A. Olejniczak, A. Katrusiak, M. Podsiadło, A. Katrusiak, *Cryst. Growth Des.*, **2019**, *19*, 1832-1838.

```
PLAT933_ALERT_2_G Number of HKL-OMIT Records in Embedded .res File         27 Note
PLAT941_ALERT_3_G Average HKL Measurement Multiplicity ...........        4.1 Low
PLAT978_ALERT_2_G Number C-C Bonds with Positive Residual Density.         12 Info
```

```
   1 ALERT level A = Most likely a serious problem - resolve or explain
   1 ALERT level B = A potentially serious problem, consider carefully
   8 ALERT level C = Check. Ensure it is not caused by an omission or oversight
  23 ALERT level G = General information/check it is not something unexpected

   2 ALERT type 1 CIF construction/syntax error, inconsistent or missing data
  13 ALERT type 2 Indicator that the structure model may be wrong or deficient
  12 ALERT type 3 Indicator that the structure quality may be low
   5 ALERT type 4 Improvement, methodology, query or suggestion
   1 ALERT type 5 Informative message, check
```

It is advisable to attempt to resolve as many as possible of the alerts in all categories. Often the minor alerts point to easily fixed oversights, errors and omissions in your CIF or refinement strategy, so attention to these fine details can be worthwhile. In order to resolve some of the more serious problems it may be necessary to carry out additional measurements or structure refinements. However, the purpose of your study may justify the reported deviations and the more serious of these should normally be commented upon in the discussion or experimental section of a paper or in the "special_details" fields of the CIF. checkCIF was carefully designed to identify outliers and unusual parameters, but every test has its limitations and alerts that are not important in a particular case may appear. Conversely, the absence of alerts does not guarantee there are no aspects of the results needing attention. It is up to the individual to critically assess their own results and, if necessary, seek expert advice.

Publication of your CIF in IUCr journals

A basic structural check has been run on your CIF. These basic checks will be run on all CIFs submitted for publication in IUCr journals (*Acta Crystallographica*, *Journal of Applied Crystallography*, *Journal of Synchrotron Radiation*); however, if you intend to submit to *Acta Crystallographica Section C* or *E* or *IUCrData*, you should make sure that full publication checks are run on the final version of your CIF prior to submission.

Publication of your CIF in other journals

Please refer to the *Notes for Authors* of the relevant journal for any special instructions relating to CIF submission.

Validation response form

Please find below a validation response form (VRF) that can be filled in and pasted into your CIF.

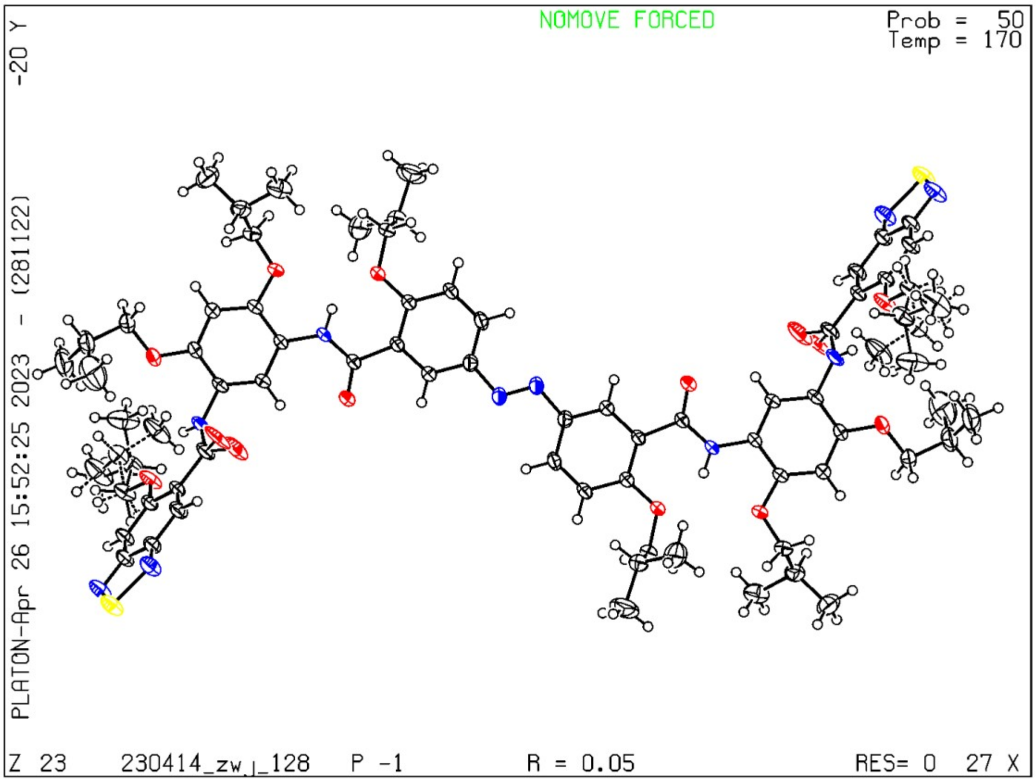

NOMOVE FORCED
Prob = 50
Temp = 170
-20 Y
PLATON-Apr 26 15:52:25 2023 - (281122)
Z 23
230414_zwj_128
P -1
R = 0.05
RES= 0 27 X
